# Global Marine Fishery Stock Productivity Under Climate Change

**DOI:** 10.1111/gcb.70784

**Published:** 2026-03-09

**Authors:** Shuyang Ma, Geir Huse, Kotaro Ono, Maud Alix, Yongjun Tian, Paul J. B. Hart, Olav Sigurd Kjesbu

**Affiliations:** ^1^ Institute of Marine Research Bergen Norway; ^2^ Ocean University of China Qingdao China; ^3^ University of Leicester Leicester UK

**Keywords:** environmental effect, FAO areas, fish population dynamics, global warming, risk assessment, surplus production

## Abstract

Marine capture fisheries play crucial roles in global aquatic protein supply and livelihoods of millions of people. Anthropogenic climate change comes as an overlying threat, potentially necessitating substantial adjustments of harvest control rules or rebuilding plans, especially for species (stocks) that are naturally adapted to restricted environmental fluctuations. Stock productivity, defined as surplus production provided by per unit of stock biomass, offers an informative yet underutilized metric for assessing these impacts. With the help of global fishery‐related databases and earth system models, stock productivity estimates were related to key biophysical drivers by state‐of‐the‐art statistical methods. The ultimate goal thereby is to clarify how climate change has affected and will continue to affect this harvest potential. Results show that the hindcasted global stock productivity (710 stocks) exhibited pronounced stock‐specific and regional heterogeneity, with signs of an overall decline (1980–2022). Variations in sea temperature and chlorophyll concentration significantly affected the productivity of about half of the assessed stocks (1993–2020). The subsequent productivity projections indicated relatively moderate reductions in the global mean productivity proxy (2021–2100), though these projections were characterized by uncertainty and with different data availability depending on the regions. However, the important finding of a general balanced prevalence of stock ‘winners’ and ‘losers’ lessened this regional quantification problem. As inferred, by the end of the century, global productivity (also applied to fishery landings) is projected to decline by 3.0% (−6.3% to +0.4%) under a ‘business‐as‐usual’ scenario and 1.0% (−1.6% to −0.3%) under a ‘sustainability’ scenario. Thus, our research indicates relatively moderate effects of climate change on the global fisheries productivity, though with the above‐mentioned existence of clear winners and losers. This finding contrasts with previous investigations that depict remarkable declines in future fishery landings.

## Graphical abstract

In this uniquely extensive global analysis on the productivity of commercial fishery stocks, a common factor was the existence of pronounced heterogeneity, as documented for 710 stocks in the undertaken hindcast over the last decades. Importantly, both winners and losers appear under on‐going climate change, and then seemingly are equally present across waters, that is, at *ca*. 1:1. Consequently, the noticed shortage of stock inventories in some regions did not methodologically impede scaling up to global trends; the subsequent projections for 657 stocks indicated relatively moderate reductions in the global mean productivity proxy, a result in contrast with commonly conveyed messages.

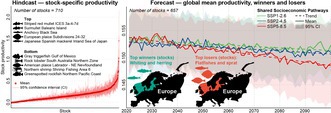

## 1 Introduction

Marine organisms are evidently facing multiple challenges under anthropogenic climate change (Cheung et al. 2013; Doney et al. 2012; Litzow et al. 2024; Ramírez et al. 2017; Tittensor et al. 2021). The corresponding capture fisheries, which explicitly utilise these natural ecosystem resources, contribute substantially to global food security (Costello et al. 2020), exemplified by the 79.7 million tonnes which were landed in 2022 (FAO 2024). Consequently, an expressed interest and underlying concern of this sector is how climate‐induced population dynamics will translate into fisheries management and thereby quota settings (Barange et al. 2014; Cao et al. 2023; Sumaila et al. 2011), a target highlighted by the Sustainable Development Goal 14 ‘Life Below Water’ (UN DESA 2024). Mechanistically addressing these implications requires a comprehensive approach, as recognized by many research consortia, developing and running advanced earth‐system and/or ecosystem models scaling up from lower to higher trophic levels (Blanchard and Novaglio 2024; Eyring et al. 2016; Heneghan et al. 2021). Another analytic approach is to focus more directly on how the stock per se has already responded to ongoing climate change to indicate what its status will be in the future depending upon climate scenarios (Cheung et al. 2021; Hollowed et al. 2013; Lam et al. 2020; Ma, Huse, Ono, Nash, et al. 2024). This rationale is derived from that many stocks are closely monitored so producing reasonably robust and lengthy time series (RAM Legacy Stock Assessment Database 2023). The obvious drawback is the relatively lower emphasis on the underlying, complex mechanistic processes. Thus, building on findings from the other types of models just mentioned stands out as important when undertaking these complementary studies targeting the role of environmental drivers on fishery population dynamics.

Within this branch of fishery oceanography, stock productivity is an essential concept, with direct relevance for tactical stock monitoring as well as recovery and rebuilding plans (Hammer et al. 2010; Hilborn and Walters 2013; Worm et al. 2009). Although the definition of stock productivity is rather straightforward, the actual quantification might be challenging, not only limited by methodological progress but also depending on the data quality and quantity at hand. Theoretically, the three vital parameters—natural mortality, recruitment and body growth—should then be considered (Pitcher and Hart 1982). However, pinpointing these underlying and often evasive components of life history, such as natural mortality, is notoriously difficult (Maunder et al. 2023; Punt et al. 2021). Instead of attempting to evaluate these three parameters, surplus production models (SPMs)—working at the integrated trait level—have been advocated as a way to profile stock productivity dynamics (Pitcher and Hart 1982). By integrating the general effects of natural mortality, recruitment, and body growth into a single production function, SPMs facilitate insights into the temporal evolution of the stock biomass targeted by fishing (Cousido‐Rocha et al. 2022). Thus, the observed surplus production is given as the annual change in stock biomass—defined as body growth plus the biomass of new recruits minus the losses attributed to natural mortality—together with the corresponding biomass removed by fishing (Cousido‐Rocha et al. 2022). SPMs are therefore believed to provide a feasible way to quantify stock productivity (Free et al. 2019), where the additional standardization of surplus production relative to stock biomass allows for a more consistent assessment of the stock's health and potential (Ma, Huse, Ono, Nash, et al. 2024). An additional argument in favour of SPMs is that the relatively low data requirements facilitate its application to marine fishery stocks worldwide, especially for areas with low monitoring efforts. However, as always, administrative restrictions on the free availability of data might occur, hindering the fuller insight in parts of the world's ocean. These shortcomings necessarily translate to the quality of the resulting annual global productivity figure, which thereby should be considered as a proxy, but, logically, not so for any informed messages at the single stock level. A solution to examples of missing data is thereby to focus on rates of productivity change instead of the absolute figures as such, supplemented by aiming at the broader principles for climate‐induced alterations in this rate dynamics.

Based on these considerations, we ran SPMs to reconstruct productivity of more than 700 marine commercial stocks in the world's oceans, and advanced statistical tools were applied to examine the productivity dynamics under global climate change. Archived and new information from climatology, oceanography, biophysics and population dynamics were assembled to present the magnitude of and associated variability in stock productivity over time. These most extensive databases were analysed to unravel how climate change has and most likely will affect these productivity measures. More concretely, we considered stock productivity changes both in the past decades (hindcasts) and by the end of the 21st century (forecasts). With this in place, our main objectives were (1) to reveal stock‐ and regional‐specific response directions, (2) evaluate potential ‘winners’ and ‘losers’ (Griffiths et al. 2017; Sailley et al. 2025), and (3) point out management priorities to promote conservation efforts on where they are most needed.

## 2 Materials and Methods

### 2.1 Data Sources

Fishery (assessment) stock time series—including catch, landings, total biomass, total abundance, spawning stock biomass, catch per unit effort (CPUE, i.e., fishery‐dependent data) and survey abundance (i.e., fishery‐independent data)—were sourced from the RAM Legacy Stock Assessment Database (https://www.ramlegacy.org/) (RAM Legacy Stock Assessment Database 2023). This database presently consists of altogether 1436 stocks. The further stock selection was based on the following four criteria: (1) one index time series of catch (catch or landings) and at least one index time series of abundance (out of the five kinds given above) should be in place, both of which are mandatory input data when estimating stock productivity, (2) the catch index time series should span at least 15 years to support model robustness, (3) stocks should be identified at the species level to collect essential life‐history traits (biological parameters), and (4) the analysis should consistently be restricted to the marine realm. Applying these criteria—dealt with in more details below—resulted in an aggregated dataset comprising of 752 stocks (298 species).

The spatial distributions of the selected stocks were obtained from the Global Record of Stocks and Fisheries (GRSF) database (https://i‐marine.d4science.org/web/grsf/home), which gives coordinates forming polygons. Matching between the GRSF and RAM Legacy Stock Assessment Database was facilitated by the alpha‐numerical universally unique identifier (GRSF_uuid) assigned to each stock. The centroids were calculated with the R package ‘terra’ (Hijmans 2022), providing the inhabited area for each of a total of 682 stocks (275 species).

Biophysical variables, including temperature (T), salinity (S), sea surface height (SSH), mixed layer depth (thickness) (MLD), chlorophyll *a* (hereafter chlorophyll, or CHL), dissolved oxygen (DO), net primary production (NPP), and pH, were collected from the EU Copernicus Marine Service Information (CMEMS). In more detail, the first four variables were compiled from the Global Ocean Ensemble Physics Reanalysis (https://data.marine.copernicus.eu/product/GLOBAL_MULTIYEAR_PHY_ENS_001_031/services), whereas the latter four were from the Global Ocean Biogeochemistry Hindcast (https://data.marine.copernicus.eu/product/GLOBAL_MULTIYEAR_BGC_001_029/services). These data, available monthly from January 1993 to December 2020, come with a spatial resolution of 0.25°, grouped into 75 depth layers. Except for SSH and MLD, which both referred to a single depth layer, the values of other variables were averaged across depth layers shallower than 50 m. After this routine, all biophysical variable data were averaged for each stock over its inhabited area (see above) and then aligned with the corresponding stock time series. The analytic effort yielded valid biophysical variable values for a total of 673 stocks (272 species).

### 2.2 Analyses

**FIGURE 1:**
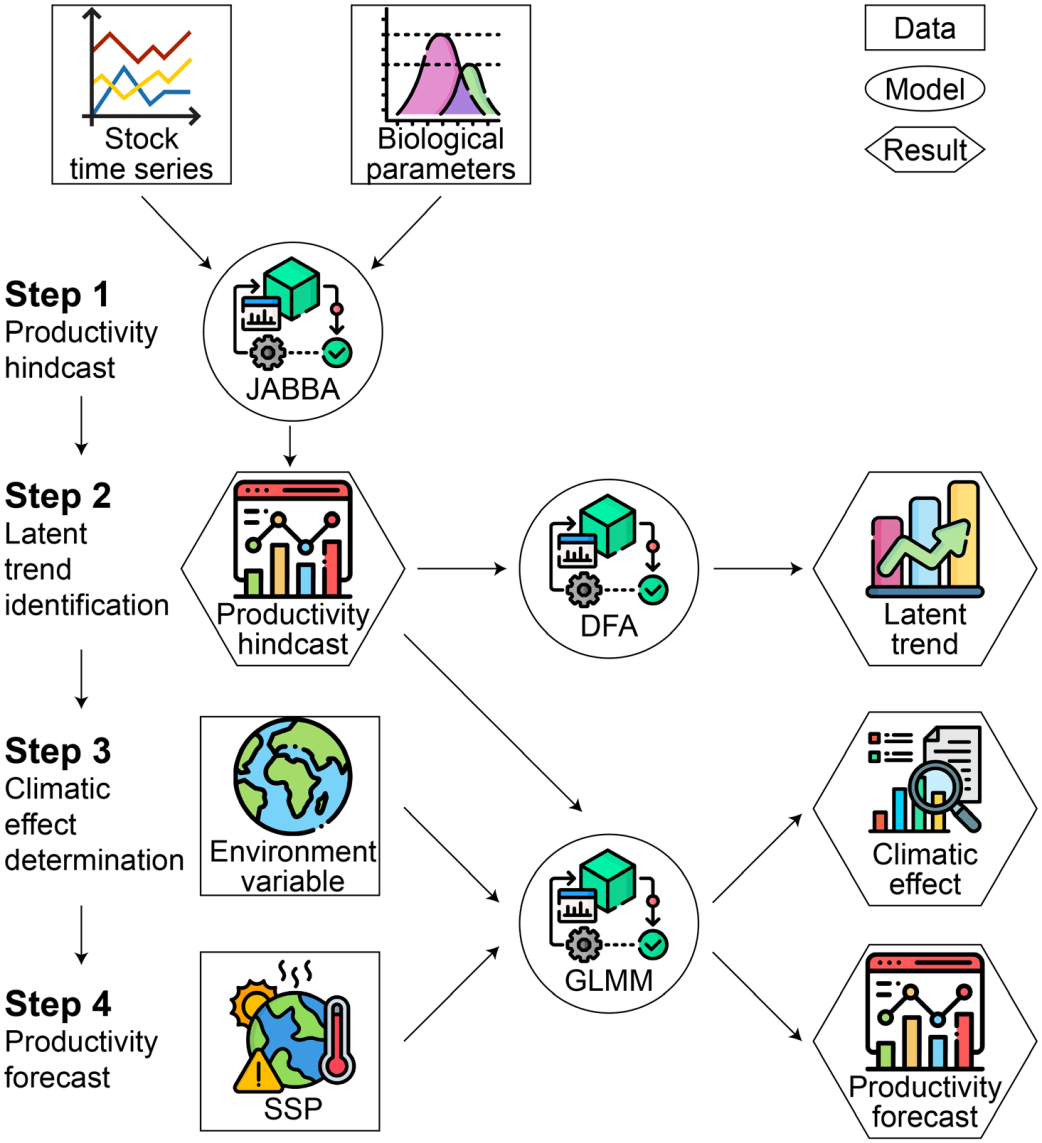
Analytical framework. DFA, Dynamic Factor Analysis; GLMM, Generalized Linear Mixed Model; JABBA, Just Another Bayesian Biomass Assessment; SSP, Shared Socioeconomic Pathway.

A four‐step analytical framework was developed, including productivity hindcast, latent trend identification, climatic effect determination and productivity forecast (Figure 1).

#### 2.2.1 Productivity Hindcast

Surplus production models (SPMs), including the Schaefer (1954), Fox (1970) and Pella and Tomlinson (1969) variants, were fitted using Just Another Bayesian Biomass Assessment (JABBA) (Winker et al. 2018), utilizing the R package ‘JABBA’ (Winker et al. 2023). Stock productivity was defined as ‘observed’ surplus production divided by the total stock biomass (TSB), where the observed surplus production was calculated as the biomass difference between 2 years plus catch. Hence, this term reflects the biomass that can be provided by a unit of TSB, integrating body growth, mortality, and recruitment, and providing a comprehensive proxy for the productive potential of stocks (Ma, Huse, Ono, Nash, et al. 2024). It is worth noting that although the productivity proxy could be directly calculated for stocks with available total biomass and catch data, the SPMs were still fitted for several reasons: (1) only a subset of stocks had total biomass data, while data‐poor stocks had only CPUE or survey‐based abundance; hence, SPMs enabled full utilization of this valuable dataset; (2) for stocks with total stock biomass, the biomass time series were generally shorter than the catch data (as stock assessments typically began later than catch statistics); hence, SPMs allowed us to extend the analysis to earlier periods; (3) because various stock assessment methods were used across different stocks to estimate total biomass, SPMs provided a unified analytical framework and ensured consistency in the estimation of productivity. As defined above, the input data for JABBA had to consist of a catch index and at least one abundance index. Catch data were prioritized over landings when both were available, due to its implicit incorporation of discards. All available abundance indices were incorporated into JABBA.

Critical biological parameters for fitting SPMs included the intrinsic rate of population increase (*r*), carrying capacity (*K*) and initial biomass depletion at the start of the available catch index time series (*ᴪ*). All priors were specified as having a log‐normal distribution (Winker et al. 2018). Priors for *r* of stocks were extracted from FishBase (https://www.fishbase.se/search.php) (Froese and Pauly 2024) and SeaLifeBase (https://www.sealifebase.se/search.php) (Palomares and Pauly 2024), with prior means and 95% confidence intervals being provided. For stocks lacking such information, priors for *r* was translated from resilience categories that were available in FishBase and SeaLifeBase, following established literature (Table 1) (Froese et al. 2020). In these cases, the lower and upper limits (forming the 95% confidence intervals) were first obtained, subsequently, other prior parameters, such as mean, standard deviation and coefficient of variation, were calculated. Priors for *K* of stocks were calculated using empirical formulae based on priors for *r* and catch index, applying a 3‐year moving average to mitigate the influence of extreme values (Table 1) (Froese et al. 2017). The biomass level at the end of the time series, which was related to the calculation of the prior parameter for *K*, was estimated by comparing the average *B*/*B*_MSY_ index (with MSY being the maximum sustainable yield) in the last 3 years to 1.0, with a value > 1.0 indicating a high biomass, while a value < 1.0 was associated with low biomass. For stocks without *B*/*B*_MSY_ information, abundance indices were used instead. Specifically, the ratio between the mean of the last 3 years and the historical maximum was calculated. This ratio was then compared to 0.5: values greater than 0.5 indicated a high biomass level, whereas values less than 0.5 indicated a low biomass level. For stocks with multiple abundance indices yielding contradictory results, a majority‐rule approach was adopted, whereby the minority followed the majority. Priors for *ᴪ* were translated from qualitative stock size information (Table 1) (Froese et al. 2020), following an order of *B*/*B*_MSY_ based, abundance‐based and catch‐based approaches. Specifically, the mean *B*/*B*_MSY_ of the initial 3 years was firstly assessed and the qualitative stock size was categorized as: ‘Very small’ if *B*/*B*_MSY_ ≤ 0.25, ‘Small’ if 0.25 < *B*/*B*_MSY_ ≤ 0.75, ‘About half’ if 0.75 < *B*/*B*_MSY_ ≤ 1.25, ‘More than half’ if 1.25 < *B*/*B*_MSY_ ≤ 1.75 and ‘Close to unexploited’ if *B*/*B*_MSY_ ≥ 1.75. For stocks without available *B*/*B*_MSY_ or *B*/*B*_MSY_ could not match with the catch time series (as stock assessments typically postdate catch records), the ratio of the mean of abundance index values in the initial 3 years to the historical maximum abundance index value (*A*/*A*_MAX_) was evaluated, and then the qualitative stock size was judged as: ‘Very small’ if *A*/*A*_MAX_ ≤ 0.20, ‘Small’ if 0.20 < *A*/*A*_MAX_ ≤ 0.40, ‘About half’ if 0.40 < *A*/*A*_MAX_ ≤ 0.60, ‘More than half’ if 0.60 < *A*/*A*_MAX_ ≤ 0.80 and ‘Close to unexploited’ if *A*/*A*_MAX_ ≥ 0.80. Finally, for stocks without any *A*/*A*_MAX_ that could match the starting points of catch time series, the ratio of the mean catch in the initial 3 years to the historical maximum catch (*C*/*C*_MAX_) was calculated and the qualitive stock size set as: ‘Very small’ if *C*/*C*_MAX_ ≥ 0.80, ‘Small’ if 0.60 < *C*/*C*_MAX_ ≤ 0.80, ‘About half’ if 0.40 < *C*/*C*_MAX_ ≤ 0.60, ‘More than half’ if 0.20 < *C*/*C*_MAX_ ≤ 0.40 and ‘Close to unexploited’ if *C*/*C*_MAX_ ≤ 0.20. Though catch‐based methods used in evaluating abundance have been criticized as catch is related to not only abundance but also economics, management and other multiple factors (Branch et al. 2011; Daan et al. 2011; Pauly et al. 2013), it represents the best available information we can get access to. Priors for these three parameters were compiled for 728 stocks (288 species).

**TABLE 1:** Setups of priors for biological parameters. The prior for intrinsic rate of population increase (*r*) was translated from resilience categories in FishBase or SeaLifeBase, whereas the prior for carrying capacity (*K*) was calculated based on catch and priors for *r*. The prior for initial biomass depletion at the start of the available catch indices time series (*ᴪ*) was translated from qualitative stock size information.

Biological parameter	Criterion	Category	Prior parameter: lower limit	Prior parameter: upper limit
*r*	Resilience	Very low	0.015	0.10
		Low	0.05	0.50
		Medium	0.20	0.80
		High	0.60	1.50
*K*	Biomass level at the end of the time series	High	2Max(Catch)/*r*_upper‐limit_	12Max(Catch)/*r*_lower‐limit_
		Low	Max(Catch)/*r*_upper‐limit_	4Max(Catch)/*r*_lower‐limit_
*ᴪ*	Qualitative stock size information	Very small	0.01	0.20
		Small	0.15	0.40
		About half	0.35	0.65
		More than half	0.50	0.85
		Close to unexploited	0.75	1.00

The model settings involved defining observation and process error variances, as well as assigning fitting procedures. In the absence of standard error or confidence intervals for the abundance indices, the minimum fixed observation error variance was set to 0.1 and the additional observation error variance was estimated. As no specific prior knowledge is available, the process error variance was assigned a prior that was an inverse‐gamma distribution with both scaling parameters equal to 0.001, to approximate a uniform distribution on the log scale (Winker et al. 2018). It should be noted that so‐called flat prior may still be informative and may lead to biased posterior parameter estimates (Lemoine 2019). The models were fitted using three Markov Chain Monte Carlo (MCMC) chains, each with 200,000 iterations, a burn‐in period of 100,000 iterations and a thinning rate of 50 iterations. The best SPM was selected based on the Deviance Information Criterion (DIC), with lower DIC values indicating better model fit (Spiegelhalter et al. 2002).

Model diagnostics involved verifying the convergence of MCMC chains and the unimodality of posterior parameter distributions. Convergence was evaluated by comparing the improved Gelman–Rubin Statistics (*Rhat*) to 1.05, with values less than this threshold indicating successful parameter estimation convergence (Vehtari et al. 2021). Unimodality was based on Hartigan's Dip Test, where a *p*‐value greater than 0.05 signified unimodality (Hartigan and Hartigan 1985). Ultimately, successful model fits were achieved for a total of 710 stocks (285 species). In addition, to explore whether the model fitting results were either largely dependent on the prior distributions of parameters (*r*, *K* and *ᴪ*) leading to prior‐driven results or relying on the data resulting in data‐driven results, the posterior‐to‐prior mean and variance ratio (PPMR and PPVR) of the three parameters were investigated. If PPMR and PPVR were close to 1, posterior distributions largely followed prior distributions and thereby merely reflected prior‐driven results. Contrastingly, if their differences to 1 exceeded a certain threshold, the data were considered informative in updating posterior from prior distributions and providing data‐driven results. The threshold identification was based on simulation experiments (Figure S1), with details provided in the Supplementary texts.

To address uncertainty in stock productivity hindcast, 1000 sets of estimated parameters were randomly sampled from their posterior distributions (from 6000 sets saved) and thereafter used to construct 1000 SPMs. Consequently, 1000 productivity candidate time series were generated for each stock. In the following model work, these time series were utilized for assessing the uncertainty originated from the hindcast.

Linear regression was employed to identify long‐term patterns in stock productivity, utilizing the in‐built R package ‘stats’ (R Core Team 2022). As the productivity time series varied in length across stocks, the analytical period was defined from 1981 to 2022, with special focuses on the more recent patterns. A significant trend in stock productivity was determined if the 95% confidence interval of the regression slope did not contain zero.

#### 2.2.2 Latent Trend Identification

The primary, underlying patterns (latent trends) in stock productivity were detected by Dynamic Factor Analysis (DFA) with the R package ‘MARSS’ (Holmes et al. 2024). DFA reduces the dimensionality of multivariate time series (here stock productivity) by estimating a set of latent trends and corresponding loadings, representing the linear effects of each trend on the time series (Zuur et al. 2003). This statistical tool consists of two models, one process model and one observation model. The process model describes any latent trend as random walks:

$$x_{i,t+1}=x_{i,t}+w_{i,t}$$

where *x*_*i,t+1*_ is the value of the *i*‐th latent trend at year *t + 1*, *x*_*i,t*_ is the *i*‐th trend value at year *t*, and *w*_*i,t*_ is the deviation (white noise) at year *t* and modelled as a multivariate normal distribution (MVN): *w*_*t*_~MVN(0, *Q*) across trends. The *Q* is fixed as an identity matrix for identifiability, also meaning that the latent trends are independent of each other. Next, the observation model links these latent trends to a response variable:

$$y_{j,t}=\sum_{i=1}^{n}Z_{j,i}x_{i,t}+e_{j,t}$$

where *y*_*j,t*_ currently is the productivity of stock *j* at year *t* and modelled as continuous and normally distributed, *Z*_*j,i*_ is the loading of trend *i* on productivity time series of stock *j*, and *e*_*j,t*_ is the observation error of stock *j* at year *t* and modelled as a MVN: *e*_*t*_~MVN(0, *R*) across stocks. The *R* is an estimated variance–covariance matrix allowed equal or unequal variance, and covariance or no covariance among stocks.

For each FAO major fishing area (Area), six DFAs were fitted to stock productivity. These models allowed for one or two latent trends and three types of variance–covariance matrices: same variances and no covariance, different variances and no covariance, and same variances and same covariance. Hence, to ensure model convergence, the most complex structure—different variances and different covariances—was currently left out. To make the results interpretable, the maximum number of trends was restricted to two, labelled as Trend 1 and Trend 2. In a pilot analysis, the three‐trend DFA set‐ups were tested on Areas 27 (Northeast Atlantic; with the most stocks studied, *N* = 146) and 77 (Eastern Central Pacific; towards the least stocks studied, *N* = 28). These models showed similar trends to the two‐trend DFAs, indicating the robustness of the detected trends. Model selection was conducted by comparing the log‐likelihood (logLik) and the sample‐size‐corrected Akaike Information Criterion (AICc) (Cavanaugh 1997).

#### 2.2.3 Climatic Effect Determination

Aiming at determining the effects of biophysical variables on stock productivity, the Generalized Linear Mixed Model (GLMM) was consulted using the R package ‘glmmTMB’ (Brooks et al. 2024). Three types of GLMM structures were considered: (1) fixed slopes and intercepts assuming that climatic effects were the same across stocks, (2) fixed slopes and random intercepts assuming consistent climatic effects but different baselines across stocks, and (3) random slopes and intercepts assuming that climatic effects may vary in clearly dissimilar ways across stocks. Any collinearity issues between biophysical variables were addressed by Pearson correlation analysis with the R package ‘psych’ (Revelle 2024). This test was performed on stock‐related annual means, and the resulting correlation coefficients were aggregated to determine the overall distribution pattern. The degree of collinearity was evaluated by comparing the distribution of correlation coefficients against significant thresholds. The thresholds were set at 0.374 and 0.478, corresponding to two‐sided Student's *t*‐test *p*‐values of 0.05 and 0.01, respectively, with 26 (28–2) degrees of freedom. The T, CHL and MLD were selected as independent biophysical variables and used in the following analyses. This analytical design resulted in a total of eight models:

**Table:** 

1	$P_{i}=\alpha +\beta_{T}T+\beta_{\mathrm{CHL}}\mathrm{CHL}+\beta_{\mathrm{MLD}}\mathrm{MLD}+\varepsilon$	$\varepsilon \sim N\left(0,\sigma_{\mathrm{obs}}^{2}\right)$. $\alpha_{i}\sim N\left(0,\sigma_{\alpha }^{2}\right)$. $\beta_{T,i}\sim N\left(0,\sigma_{T}^{2}\right)$. $\beta_{\mathrm{CHL},i}\sim N\left(0,\sigma_{\mathrm{CHL}}^{2}\right)$. $\beta_{\mathrm{MLD},i}\sim N\left(0,\sigma_{\mathrm{GSP}}^{2}\right)$
2	$P_{i}=\alpha +\beta_{T}T+\beta_{\mathrm{CHL}}\mathrm{CHL}+\beta_{\mathrm{MLD}}\mathrm{MLD}+\alpha_{i}+\varepsilon$	
3	$P_{i}=\alpha +\beta_{T}T+\beta_{\mathrm{CHL}}\mathrm{CHL}+\beta_{\mathrm{MLD}}\mathrm{MLD}+\alpha_{i}+\beta_{T,i}T+\varepsilon$	
4	$P_{i}=\alpha +\beta_{T}T+\beta_{\mathrm{CHL}}\mathrm{CHL}+\beta_{\mathrm{MLD}}\mathrm{MLD}+\alpha_{i}+\beta_{\mathrm{CHL},i}\mathrm{CHL}+\varepsilon$	
5	$P_{i}=\alpha +\beta_{T}T+\beta_{\mathrm{CHL}}\mathrm{CHL}+\beta_{\mathrm{MLD}}\mathrm{MLD}+\alpha_{i}+\beta_{\mathrm{MLD},i}\mathrm{MLD}+\varepsilon$	
6	$P_{i}=\alpha +\beta_{T}T+\beta_{\mathrm{CHL}}\mathrm{CHL}+\beta_{\mathrm{MLD}}\mathrm{MLD}+\alpha_{i}+\beta_{T,i}T+\beta_{\mathrm{CHL},i}\mathrm{CHL}+\varepsilon$	
7	$P_{i}=\alpha +\beta_{T}T+\beta_{\mathrm{CHL}}\mathrm{CHL}+\beta_{\mathrm{MLD}}\mathrm{MLD}+\alpha_{i}+\beta_{T,i}T+\beta_{\mathrm{MLD},i}\mathrm{MLD}+\varepsilon$	
8	$P_{i}=\alpha +\beta_{T}T+\beta_{\mathrm{CHL}}\mathrm{CHL}+\beta_{\mathrm{MLD}}\mathrm{MLD}+\alpha_{i}+\beta_{\mathrm{CHL},i}\mathrm{CHL}+\beta_{\mathrm{MLD},i}\mathrm{MLD}+\varepsilon$	

where *P*_*i*_ is productivity time series of stock *i*, *α* is intercept, *β*_*variable*_ is fixed slope and *ε* is error term. For the extension of random effects, *α*_*i*_ is random intercept and *β*_*variable,i*_ is random slope. Due to model convergence issues, species were set as random factor instead of stocks, and the dispersion model accounting for heteroscedasticity was excluded. A total of 265 species, comprising 652 stocks, were included in this analysis. For a systematic comparison of biophysical effects on stocks, only linear effects were considered in the modelling framework, further supported by the observation that such simpler approaches do as well (or even better in some cases) than more complicated approaches in forecasting the status of fishery populations (Ward et al. 2024). All models were fitted using a Student's *t*‐distribution with an identity link function. Model selection was based on AIC (Akaike 1987) and BIC (Bayesian Information Criterion) (Neath and Cavanaugh 2012).

To account for uncertainty in the estimation of stock productivity, the 1000 productivity candidate time series (Section 2.2.1) were used to fit the GLMMs (eight types of GLMM, in total 8000 models). Distributions of information criteria, including the AIC and BIC, were obtained, and the delta‐AIC and delta‐BIC were calculated to compare these GLMMs. Distribution of regression slope means were recorded to investigate the climatic effect, and a significant explanatory variable was defined when the 95% confidence interval—the Bayesian confidence interval indicating the uncertainty propagated from the Bayesian framework of SPMs—of the regression slope did not contain zero. It should be noted that the uncertainty in parameter estimation for each GLMM was not incorporated, as most of the uncertainties arise from the estimation of productivity proxies, and adding them would only broaden the confidence intervals without affecting the mean estimates themselves.

#### 2.2.4 Productivity Forecast

The future development in T, MLD and CHL were derived from three Earth System Models (ESMs): NorESM2‐LM (Seland et al. 2020), MPI‐ESM1‐2‐LR (Gutjahr et al. 2019) and IPSL‐CM6A‐LR (Boucher et al. 2020), successively forced by the three Shared Socioeconomic Pathways (SSPs) SSP1‐2.6, SSP2‐4.5 and SSP5‐8.5 (Riahi et al. 2017). These monthly projection data were collected from the Earth System Grid Federation (ESGF, https://esgf‐data.dkrz.de/search/cmip6‐dkrz/), from January 2021 to December 2100. We currently resampled the initial uneven grids to present uniform grids with a resolution of 1°. Consistent with the above hindcasts, the stock‐specific data were averaged according to the inhabited area in question to project the resultant stock productivity. Using the three ESMs, this methodology respectively gave access to valid forecasts for 614, 608 and 657 stocks (257, 256 and 265 species), complementing thereby the corresponding hindcast datasets.

Based on the best GLMMs (1000 models built with productivity candidate time series), 1000 projections were made for each stock from 2021 to 2100 using the future biophysical data from the three ESMs under the three SSPs. Linear regression was employed to identify trends in stock productivity forecasts, utilizing the in‐built R package ‘stats’ (R Core Team 2022). Global and area‐specific means and 95% confidence intervals were calculated under different ESMs and SSPs to portray the future global and regional productivity dynamics, with a particular focus on comparing the current situation (2020s) to the end of the century (2090s).

## 3 Results

### 3.1 Biophysical Hindcasts

**FIGURE 2:**
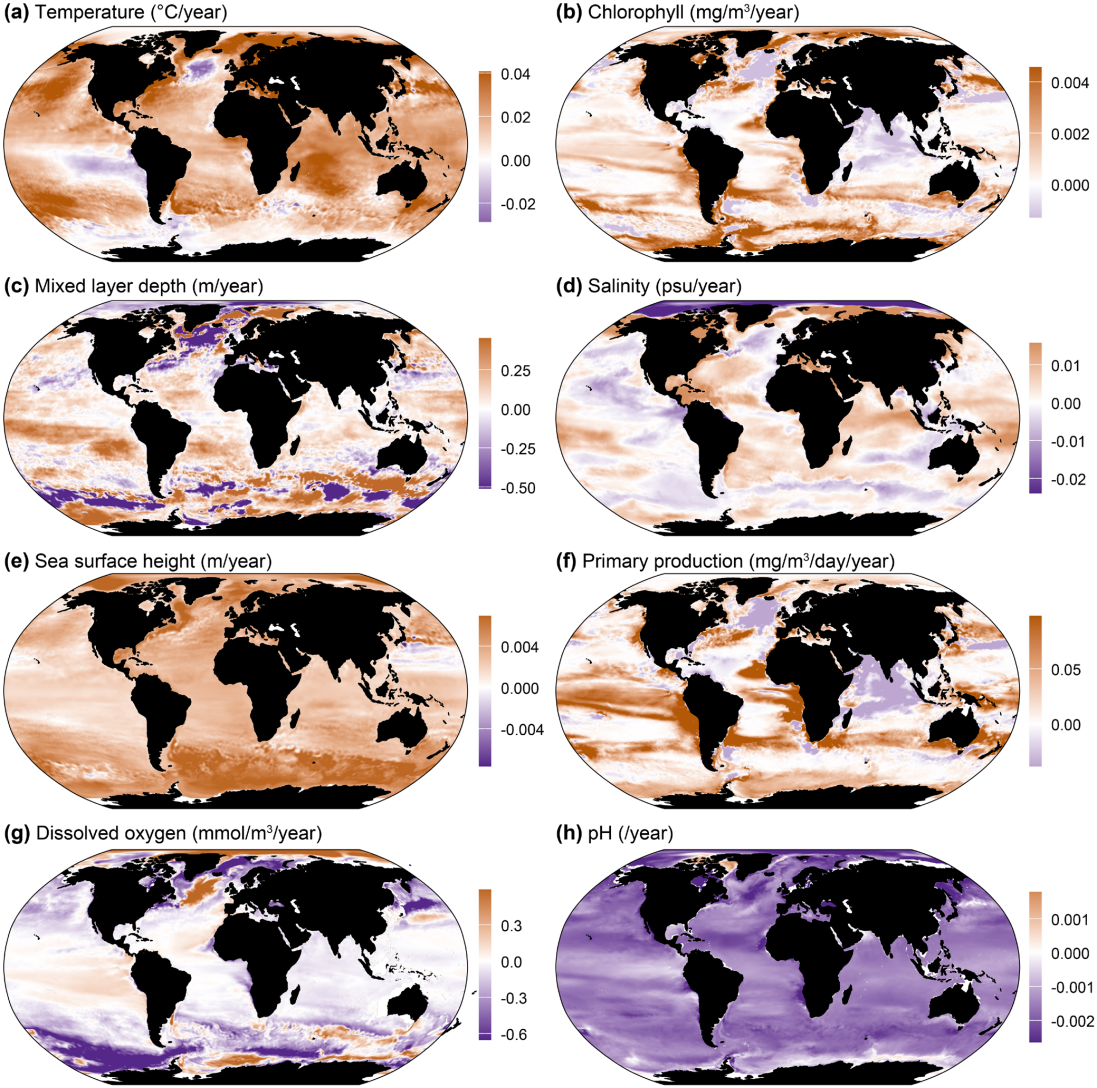
Background information on changes in global marine biophysical variables (1993–2020). The variable in question and its unit is given in the panel heading (a–h). The associated palette indicates the annual change rate. Map lines delineate study areas and do not necessarily depict accepted national boundaries.

This background synthesis illustrated the noted, large‐scale changes in eight biophysical variables over the last three decades in the marine realm (Figure 2). As widely recognized, global warming is the dominating causal factor behind these on‐going marked variations. This higher ocean temperature (Figure 2a) is accompanied by a series of other biophysical changes, but with some geographical regions clearly more affected than others (Figure 2b–h). For instance, in the Arctic, which is characterized by the strongest warming, a phenomenon known as Arctic Ocean Amplification (Shu et al. 2022) also comes with lowered salinity (Figure 2d) and increased sea surface height (Figure 2e), whereas the tropics have experienced increased salinity due to enhanced evaporation and/or altered precipitation patterns (Durack et al. 2016) (Figure 2d). Both chlorophyll concentrations and primary production indicate a rise in photosynthetic activity, though apparently not so in parts of the North Atlantic and North Indian Ocean (Figure 2b,f). As a result of the warming‐induced reductions in oxygen solubility and deep‐water ventilation (Schmidtko et al. 2017), the amount of dissolved oxygen has generally declined, particularly in the Antarctic, but with an opposite pattern in the Arctic (Figure 2g). The fall in pH seems to be omnipresent, due to increased dissolved carbon dioxide forming carbonic acid (Figure 2h). In parallel, the mixed layer depth (thickness) has enlarged, particularly in Southern Hemisphere waters (Figure 2c), affecting local nutrient mixing and light penetration.

### 3.2 Aggregated Stock Productivity Hindcasts

**FIGURE 3:**
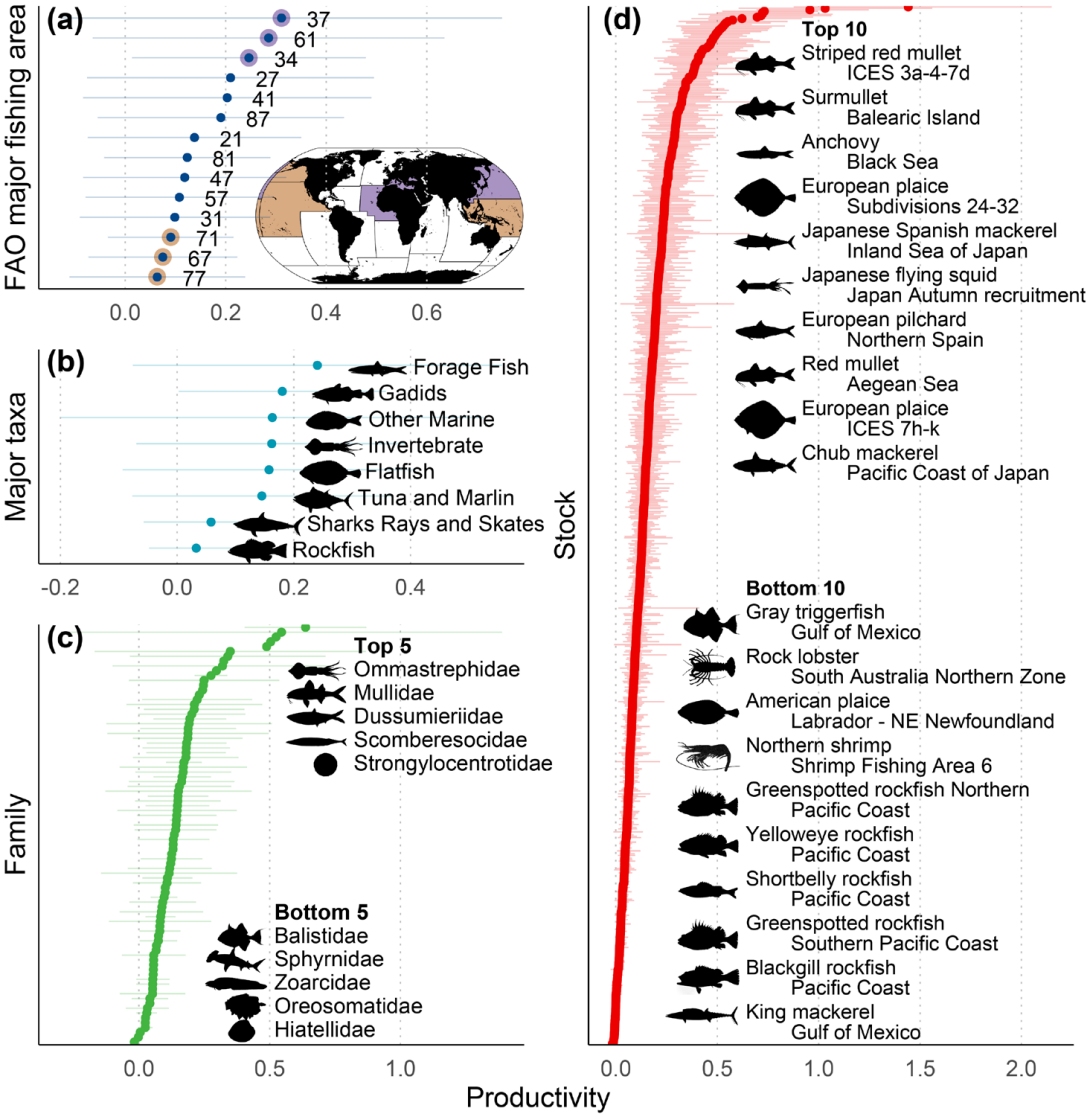
Stock productivity hindcasts by FAO major fishing area and biological taxon. The length of the studied time series varied by stock, from 15 up to 146 years (Materials and Methods, Table S2), with the output (mean and 95% confidence interval, calculated from 1000 productivity candidate time series) being filtered by (a) FAO major fishing area (*N* = 14), (b) major taxa (*N* = 8), (c) family (*N* = 87), and (d) stock (*N* = 710) labelled as in the fishery stock assessment (cf. stock ID, https://www.ramlegacy.org/). The *y*‐axis is sorted by increasing productivity. Details are provided in Tables S1 and S2. Map lines delineate study areas and do not necessarily depict accepted national boundaries.

**FIGURE 4:**
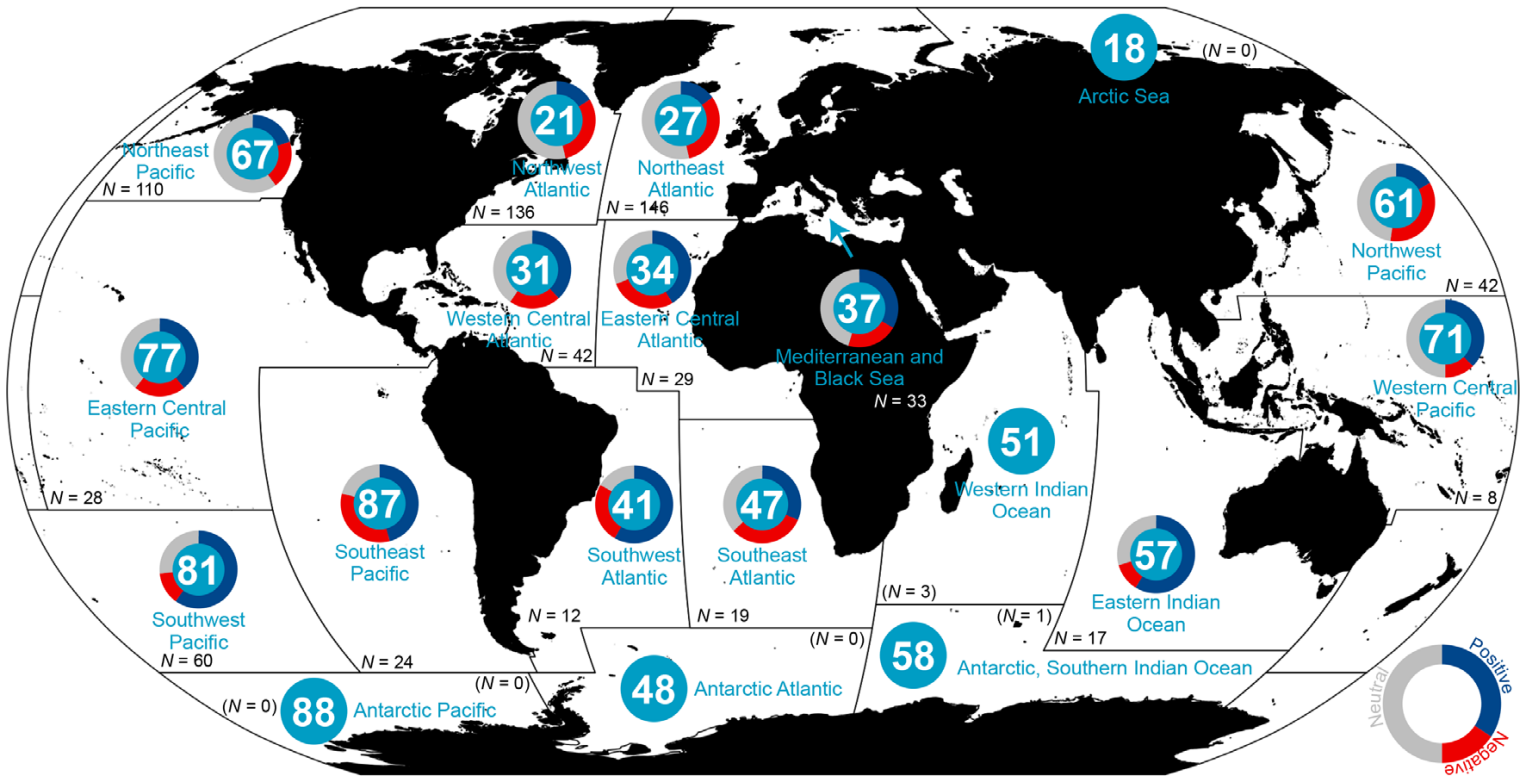
Relative proportions of hindcasted stock productivity linear patterns—negative, neutral or positive—by FAO major fishing area (Area). The significance of the temporal trend is evaluated by testing if the 95% confidence interval of the slope contains zero. *N* is the number of stocks studied in each Area. When *N* appears in parenthesis, the stock data were too limited to run the associated aggregated Area‐specific analysis. Key background and output information on every single stock underlying this article are listed in Table S2. Map lines delineate study areas and do not necessarily depict accepted national boundaries.

The JABBA models gave reliable fits, shown as clearly revised posterior distributions of key parameters comparing to prior distributions, indicating data‐driven results instead of prior‐driven ones (Supplementary texts, Figure S2). The comprehensive series on stock productivity displayed high variability within but, in relative terms, much less so across FAO major fishing areas (Areas) (Figure 3a, Table S1a). The Mediterranean and Black Sea (Area 37), Northwest Pacific (Area 61) and Eastern Central Atlantic (Area 34) were identified to be in the upper productivity range, whereas Eastern Central Pacific (Area 77), Northeast Pacific (Area 67), and Western Central Pacific (Area 71) were in the lower range (Figure 3a) (for Area location, see Figure 4). For Arctic Seas (Area 18), Antarctic Atlantic (Area 48), Western Indian Ocean (Area 51), Southern Indian Ocean (Area 58) and Antarctic Pacific (Area 88) too few stock data inventories (Supplementary texts) were presently accessible for inclusion in this broader, across‐oceans productivity comparison; that is, the near‐poles data sets were mainly missing. The Northern Hemisphere was generally associated with higher data richness than the Southern Hemisphere (Table S2).

Moving to major taxa of aquatic organisms (Figure 3b, Table S1b), forage fish (small pelagics, such as sardines, anchovies and herrings), gadids (cod fishes) and invertebrates (such as squids) occupied top positions regarding productivity. The ‘Other marine’ was generally characterized with high productivity showing considerable variation (broad confidence interval), whereas elasmobranchs (sharks, rays and skates) and viviparous bonefishes (presently limited to rockfishes) were among those with the lowest productivity. The life‐history traits of the former are characterized by relatively fast body growth, high fecundity and short generation time (i.e., *r* selection), while for the latter with low body growth, low fecundity and long generation time (i.e., *K* selection). The same *r*/*K*‐related patterns arose at the family level (Figure 3c, Table S1c), identifying saltwater clams (Hiatellidae) as low‐productivity members. For individual stocks, this *r*/*K* picture reemerged (Figure 3d, Table S2), where, for instance, anchovy, sardine (pilchard), chub mackerel and squid stocks were ranked among the ‘Top 10’, while crustacean and rockfish stocks were among the ‘Bottom 10’. Generally, for the 710 stocks analysed, about 97% (*N* = 692) presented a mean productivity of < 0.50, with 83% (*N* = 587) < 0.25 (Figure 3d, Table S2).

### 3.3 Dynamics of Stock Productivity Hindcasts

**FIGURE 5:**
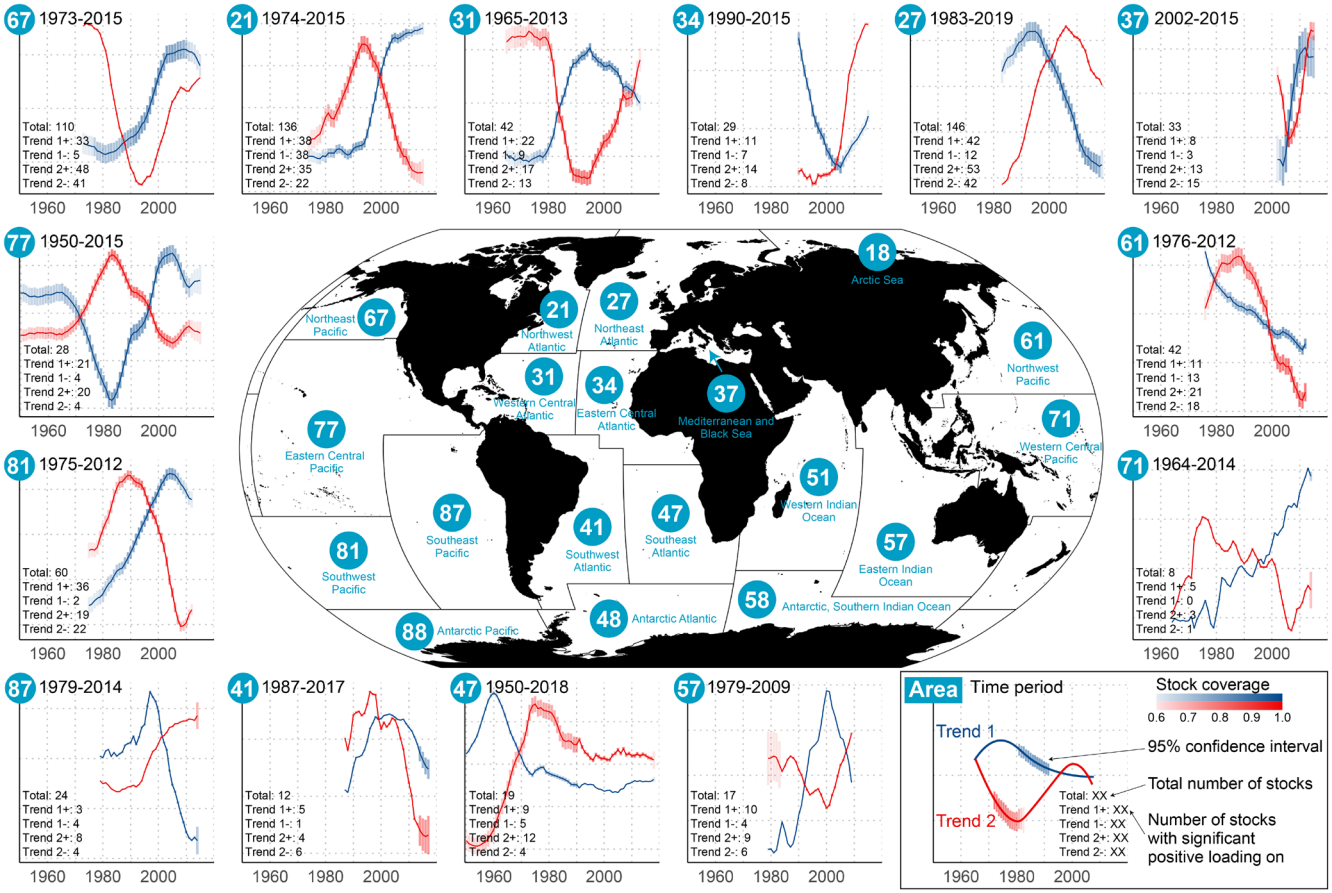
Trends in hindcasted stock productivity by FAO major fishing area (Area). A coloured line indicates a trend, with shading representing a 95% confidence interval. Stock coverage (Figure S3)—defined as the ratio of the number of stocks with available data to the total number of stocks within a given Area and year—is inversely reflected by the degree of transparency. The corresponding panel labels show Area code (see map), time window (based on stock coverage), time node (vertical, dashed 10‐year lines), total number of stocks, and the number of stocks with significant loadings on each trend. A significant loading means that the 95% confidence interval of the loadings did not contain any zero (Figure S4), with ‘+’ and ‘−’ symbols indicating positive and negative loadings, respectively. Note that *x*‐ and *y*‐axis names are hidden for visualization. Map lines delineate study areas and do not necessarily depict accepted national boundaries.

Over the last four decades (1981–2022), approximately half (*N* = 329) of the total stocks examined worldwide (*N* = 710) did not exhibit any evident, linear pattern in productivity (the slope's 95% confidence interval contained zero), whereas the remaining stocks showed a balanced presence of either positive (*N* = 199) or negative linear patterns (*N* = 182) (Figure 4, Supplementary texts). When running DFAs, a two‐trend (Trend 1 and Trend 2) model generally performed best (Table S3), detecting both general and region‐specific features (Figures 5, S3 and S4, Supplementary texts). For clarity, the loadings associated with each trend indicate the degree to which the productivity patterns of individual stocks correspond to the identified trends, that is, a positive loading signifies that the stock's productivity was aligned with the trend, whereas a negative loading indicates an inverse pattern. Overall, the stock productivity estimates—in some cases going as far back as to the 1950s—tended to decrease in the Northern Hemisphere (i.e., positive loadings on a decreasing trend) while they increased in the Southern Hemisphere (i.e., positive loadings on an increasing trend) (Figure 5, Supplementary texts).

The North Atlantic and North Pacific were represented with the highest number of stocks (Figure S5). Since the 1990s, Trend 1 in the Northwest Atlantic (Area 21) exhibited a sharp increase indicating either increased or decreased productivity in 28% of the stocks. Concurrently Trend 1 in the Northeast Atlantic (Area 27) performed differently by showing a decrease, indicating productivity decrease and increase in 29% and 8% of stocks, respectively. In the North Pacific, multidecadal‐scale variability was a dominant feature, as both Trend 2 in the Northeast and Northwest Pacific (Area 67 and 61) showed concave or convex patterns and represented 81% and 93% of stocks, with about equally distributed positive and negative loadings. In the sub‐tropical and tropical waters (Western and Eastern Central Atlantic, Western and Eastern Central Pacific), all these trends were basically characterized by decadal‐ to multidecadal‐scale variability. Moving southwards, Trend 1 in the Southwest Pacific (Area 81) indicated an increased productivity in the 1980s–2000s for 60% of the stocks. The same applied to the Southeast Pacific (Area 87) and Western Central Pacific (Area 71), but with few stocks included in the analysis and hence low representativeness. In the south Atlantic waters, the multidecadal‐scale variability contributed to the general patterns as well, though with few stocks included. For further details, see Supplementary texts.

### 3.4 Climate Versus Stock Productivity Hindcasts

Collinearity analysis among biophysical variables revealed that temperature (T), chlorophyll (CHL), and mixed layer depth (MLD) were relatively independent of each other, with respectively 71% (461), 94% (613) and 97% (632) of total stocks (652 stocks) showing no significant correlation with each other in that respect (Figure S6). Out of the eight GLMMs, the one that included the species' level effects of T and CHL (both fixed and random effects), as well as the general level effect of MLD (fixed effect), achieved the best performance (Figure S7). Over the last three decades slightly more than half of the studied species, that is, 139 out of 265 (354 out of 652 stocks) were significantly affected by climate change, as specified below.

**FIGURE 6:**
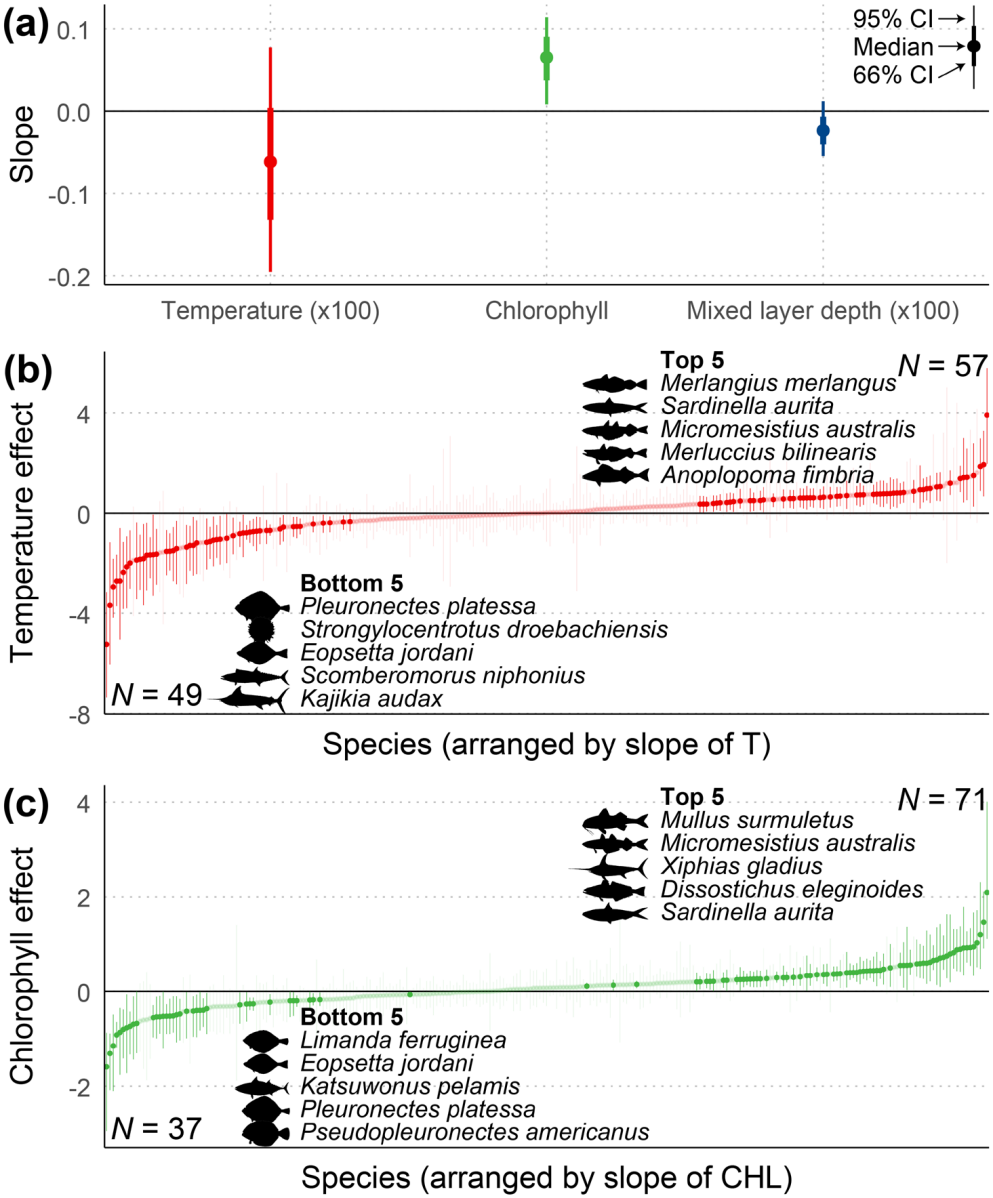
Effects of biophysical variables on hindcasted stock productivity (1993–2020). In (a), estimated slopes for temperature, chlorophyll, and mixed layer depth versus stock productivity, in the first and last instance scaled by ×100 for visualization. Thick and thin bars represent 66% and 95% confidence intervals (CIs), respectively. In (b) and (c), the species were respectively sorted by increasing temperature and chlorophyll slopes, with transparency inversely indicating the significance, defined as 95% CI (the Bayesian CI, parameters from 1000 GLMMs, indicating the uncertainty originated from productivity hindcast) without zero.

No one‐for‐all biophysical effect on stock productivity was detected, seen by targeting the recent period of unprecedented climate change (Figure 6a, Table S2). This deduction was supported by temperature being considered as the most informative variable, but which did not elicit any coherent impact across species. Despite that, > 40% (106 out of 265) of the studied species were evidently affected by temperature change (Figure 6b), as defined by zero being outside the 95% confidence interval of the regression slope for temperature vs. stock productivity. Although more species showed a significant increase in productivity with warming compared to those showing a decrease (57 vs. 49), the total number of stocks in these two response categories was almost equal (129 vs. 133). Anyhow, there was still a large number of species that did not respond significantly to warming. Generally, the temperature influence operated along latitudinal (80°N–60°S, *p* = 0.026) rather than longitudinal clines (180°W–180°E, *p* = 0.883) (Figure S8a). Furthermore, among those species that suffered, the ‘Bottom 5’ contained two flatfishes—European plaice ( *Pleuronectes platessa* ) and petrale sole (*Eopsetta jordani)—together* with green sea urchin (*Strongycentrotus droebachiensis*), Japanese Spanish mackerel ( *Scomberomorus niphonius* ), and striped marlin ( *Kajikia audax* ). For those that benefitted, the ‘Top 5’ were whiting ( *Merlangius merlangus* ), round sardinella ( *Sardinella aurita* ), southern blue whiting ( *Micromesistius australis* ), silver hake ( *Merluccius bilinearis* ), and sablefish ( *Anoplopoma fimbria* ).

In contrast to the variable temperature‐mediated productivity response, chlorophyll was predominantly linked to a positive effect (Figure 6a, Table S3). Specially, 71 species (171 stocks) exhibited positive slopes, and 37 species (105 stocks) showed negative slopes (Figure 6c). Interestingly, the spatial pattern of the relationships mirrored that for temperature and productivity. The slope of CHL increased nearly linearly (*p* = 0.0002) from Arctic to Antarctic waters (Figure S8b). For the ‘Top 5’ stocks (Figure 6c), round sardinella and southern blue whiting again appeared as above, while European plaice and petrale sole were once more ranked among the ‘Bottom 5’. In more detail, the species clearly benefited from increased chlorophyll also included striped red mullet ( *Mullus surmuletus* ), swordfish ( *Xiphias gladius* ) and Patagonian toothfish ( *Dissostichus eleginoides* ). At the other end of the response spectrum, in addition to the two flatfishes, were yellowtail flounder ( *Limanda ferruginea* ), skipjack tuna ( *Katsuwonus pelamis* ) and winter flounder ( *Pseudopleuronectes americanus* ).

Generally, the slope of mixed layer depth vs. productivity was weakly negative, associated with a reasonably narrow 95% confidence interval (Figure 6a). Due to model convergence, no random effect was presently considered.

### 3.5 Stock Productivity Forecasts

Similar to the previous hindcast, most stocks in this forecast did not show any significant directional effect in productivity (Supplementary texts, Figure S9). Chiefly, towards the end of this century the best GLMM projection—including temperature, chlorophyll, and mixed layer depth data from the three ESMs (IPSL‐CM6A‐LR, MPI‐ESM1‐2‐LR, and NorESM2‐LM)—led to harmonized patterns. The Atlantic featured the highest proportion of stocks with reduced productivity, particularly in the Northwest (Area 21) and Northeast (Area 27), where > 30% of the stocks would likely become less productive, that is, display a negative effect. The selected suite of emission scenarios—SSP1‐2.6, SSP2‐4.5 and SSP5‐8.5—provided comparable results in terms of indicated directions (Figure S9).

**FIGURE 7:**
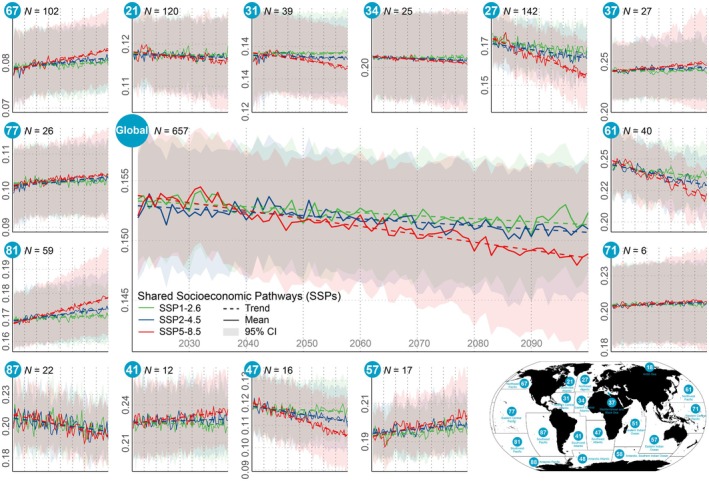
Stock productivity forecasts (2021–2100) by FAO major fishing area (Area) under three Shared Socioeconomic Pathways (SSPs) with data from IPSL‐CM6A‐LR. Coloured lines show the projected mean stock productivity (solid) and directional effect (dashed) by SSP (*p* < 0.05, two‐sided Student's *t*‐test), grouped globally or split by Area. The shading reflects the 95% confidence interval of the projected mean values from 1000 GLMMs. The total number of stocks analysed (*N*) is specified. The map insert shows the geographical location of each Area. The vertical, dotted lines are separated by 10 years, cf. *x*‐axis annotations in the global panel. Note that this panel shows proxy values due to data shortage in Area 18, 48, 51, 55 and 88. For projections by data from the other two Earth System Model (ESMs), see Figures S10 and S11. Map lines delineate study areas and do not necessarily depict accepted national boundaries.

Even so, based on the current study material, SSP5‐8.5 stood out as clearly most negative for the future global stock productivity (Figure 7, Supplementary texts, Table S4). Compared to the 2020s, this ‘business‐as‐usual’ scenario indicated a change in the mean global productivity proxy of about −0.005 (−0.010 to +0.001) by the 2090s, equivalent to −3.0% (−6.3% to +0.4%) for this proxy as such. Setting the corresponding marine exploited fish biomass to 3300 Mt. (Bianchi et al. 2021), this change equates to a reduction of 16.5 Mt. in fish landings. At the other end of the current scenarios, that is, the one for ‘sustainability’ (SSP1‐2.6), the respective figures were −0.002 (−0.003 to −0.001) and −1.0% (−1.6% to −0.3%). Within‐Area projections showed significant divergence after the middle of the century, though overall for the 14 studied Areas finding an on‐going decrease under SSP5‐8.5 but clearly less so under SSP1‐2.6 and SSP2‐4.5 (Figure 7, Table S5). However, it should be highlighted that the broad confidence intervals generally indicate substantial uncertainty and limited explanatory power, meaning that a weak Area‐aggregated change should not be interpreted as evidence for any lack of ecological response.

Regionally, the Southeast Atlantic (Area 47) was projected to experience the most significant decline in productivity up to the end of this century, around −10.5% (−16.7% to −4.6%) under SSP5‐8.5. This from‐the‐bottom (upward) ranking was followed by the Northwest Pacific (Area 61) and Northeast Atlantic (Area 27), which in turn indicated declines of −9.9% (−14.0% to −5.5%) and −9.8% (−16.1% to −3.2%). The Northwest Pacific (Area 61) contributes the largest landings to the world's capture fisheries (FAO 2024), whereas the Northeast Atlantic (Area 27) contains the greatest number of stocks included in this study, thereby ensuring representativeness. The Northeast Pacific (Area 67), also data‐rich, showed an opposite pattern; a likely higher productivity of about +5.4% (−2.6% to +13.4%) under SSP5‐8.5. The Eastern Indian Ocean (Area 57) and Southwest Pacific (Area 81) would possibly be the other two areas expected to benefit the most under this scenario, indicating productivity changes of+5.4% (−0.4% to +11.2%) and +5.4% (+0.7% to +10.7%), respectively. Returning to the foreseen impacts of SSP1‐2.6, the suggested productivity changes tended to be relatively negligible, ranging from a decrease of −3.5% (−5.4% to −1.6%) (Area 87) to an increase of 1.7% (−0.3% to +3.6%) (Area 67).

### 3.6 Future Climate ‘Winners’ and ‘Losers’

**FIGURE 8:**
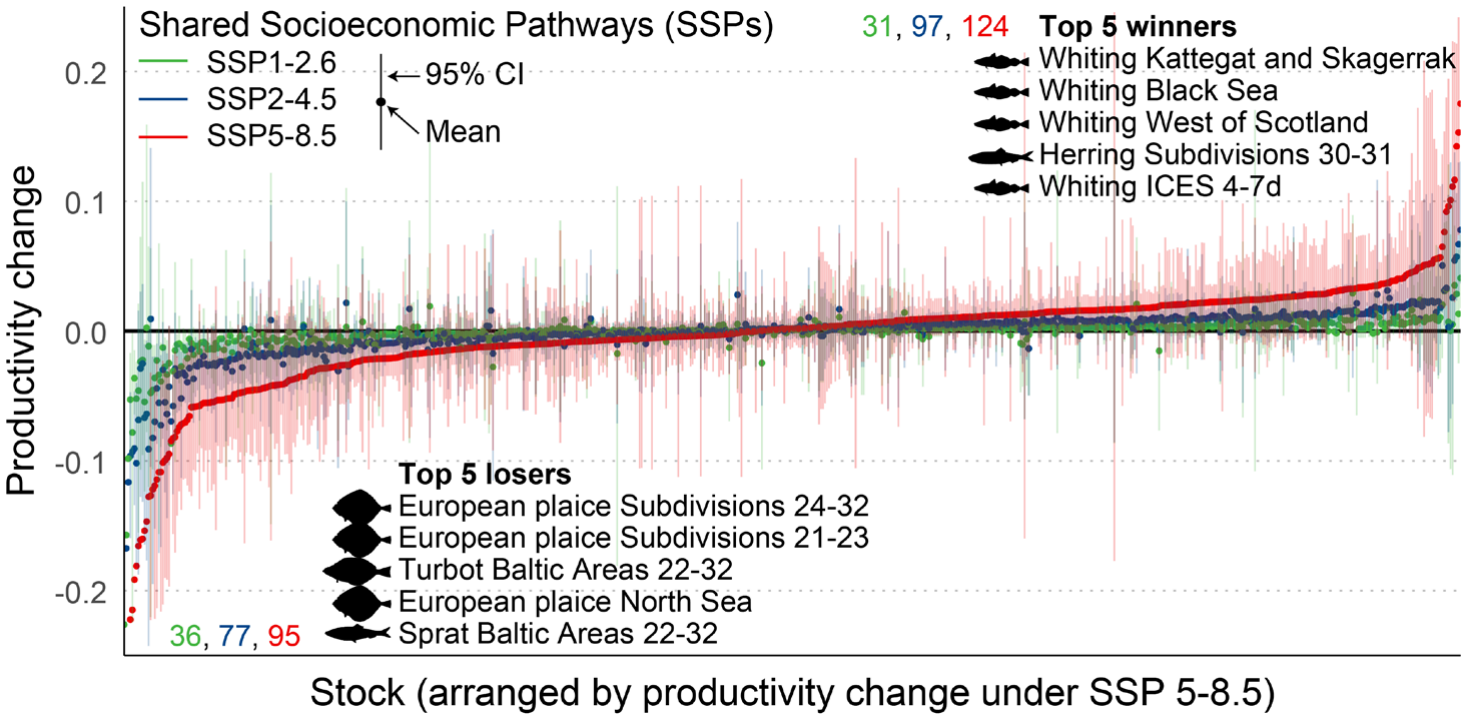
Forecasted stock winners and losers in the 2090s under three Shared Socioeconomic Pathways (SSPs). The results are presented in the context of the current situation (the 2020s). Each plotted point indicates the mean productivity change for a given stock projected with data from IPSL‐CM6A‐LR. The SSP‐resolved, stock‐specific mean productivity change is supplemented with 95% confidence interval (vertical line, projected mean values from 1000 GLMMs). The Top 5 losers and Top 5 winners under SSP5‐8.5 are listed by their assessment stock ID. The adjacent series of numbers show the total number of stocks belonging to either the winner or loser category—that is, the 95% confidence interval of projected productivity change not containing zero—split by SSP1‐2.6, SSP2‐4.5 and SSP5‐8.5. The projected productivity changes under emission scenario SSP5‐8.5 along the *y*‐axis are sorted by increasing values. The applied definition of winner and loser were according to standard practice (see Introduction); the former and latter refer to a stock projected to exhibit increased and decreased productivity, respectively. For projections with data from other Earth System Model (ESMs), see Figure S12.

The model iterations across the three SSPs for 657 stocks (265 species) projected significantly amplified stock‐specific productivity contrasts by the end of the century compared to the mid‐century (Figures 8 and S12, Table S2). Nevertheless, the number of winners and losers remained at a balanced level of about 1:1. Several stocks of whiting ( *M. merlangus* ) emerged as major winners, benefiting from both their preference for warmer temperatures and their habitats warming up. Contrastingly, two flatfishes ( *P. platessa* and *Scophthalmus maximus* ) and a sprat ( *Sprattus sprattus* ) stock in the Baltic Sea were identified as clear losers, primarily suffering from locally rising temperatures. Yet, no obvious large‐scale pattern was detected in the spatial distribution of winners and losers (Figure S13).

## 4 Discussion

This investigation was built on both hindcasts and forecasts, addressing altogether the productivity of 710 marine fishery stocks, which is by far the most comprehensive analytic effort in this regard, to our knowledge. Still, some waters (Areas) were not covered due to insufficient data. A novel, general finding was, however, the balanced presence of winners and losers. We would thereby contend that not only the presented local or regional climate‐induced productivity rates should be considered informative but also those at the global scale. Accordingly, our findings should be pertinent in discussions on the future situation for marine harvestable resources, generally and specifically.

### 4.1 Stock Productivity Hindcasts

Rather than running climate‐related simulations backwards to achieve longer productivity time series to feed into model constructs, we systematically consulted observational data to carry out the hindcasts. Thus, we presumed that the de facto detected variability rather than the length of the time series as such should be among the key analytic premises. Here it is particularly so because the studied stocks, even those with relatively short time series, have all experienced climate change to an unprecedented degree in modern times (IPCC 2023). Hence, these insights formed a solid base for the subsequent forecasts.

Stock productivity data aggregated by type of aquatic organism revealed relatively marginal differences among FAO major fishing areas (Areas); the within‐Area variability was much more pronounced, likely due to the inclusion of multiple biological taxa and the large geographical size of an Area. Trait‐based productivity patterns were found in *r*‐ vs. *K*‐selected species, although this clarification is more of a continuum than a dichotomy (Reznick et al. 2002). The *r*‐selected stocks generally demonstrated higher productivity but also higher sensitivity to climate change (Arkhipkin et al. 2015; Pikitch et al. 2014). In contrast, the *K*‐selected stocks were characterized by lower productivity and thereby inherently more vulnerable to overexploitation (Hilborn et al. 2021; Pacoureau et al. 2021). Consequently, in view of climate change, it therefore seems reasonable to continue improving the reliability of productivity predictions and projections as well as effective adaptive management strategies along this sensitivity‐vulnerability axis.

When comparing more directly our results to earlier, related ones, a study on the maximum sustainable yield (MSY) across 235 stocks seems particularly relevant, estimating a decline of 4.1% in the global total MSY between 1930 and 2010 (Free et al. 2019). It was noticed that the most significant decline started after the late 1980s/early 1990s, overlapping with the typical time series mid‐point in our stock hindcast analysis. In contrast to these MSY‐based identifications of 9 and 19 stocks respectively showing positive and negative responses to ocean warming, our outline is represented by 129 and 133 stocks. Such a substantial numerical increase may be attributed to the current shorter time window, analytically stimulated by the inherent strong magnitude of warming, and not least, the more extensive stock dataset consulted. Another relevant hindcast study examined 262 stocks, but then with recruitment capacity as an indicator of productivity uncovering, as we did, large variability among stocks and regions (Britten et al. 2016). Essentially, their meta‐analysis identified negative patterns in recruitment capacity in the North Atlantic, contrasting with the more neutral patterns emerging in the North Pacific. These findings parallel the falling stock productivity patterns presently observed in the Northwest (Area 21) and Northeast Atlantic (Area 27), and the decadal‐scale variability alongside evenly distributed stock loadings in the Northwest (Area 61) and Northeast Pacific (Area 67).

### 4.2 Climatic Effects on Stock Productivity Dynamics

Among the eight biophysical variables selected, only temperature, chlorophyll and mixed layer depth were used in the final exploration of climatic effects on stock productivity dynamics. This choice was suggested by the noticeable high collinearity between these three and the other five, that is, the subsequently omitted ones. Thus, the selected variables incorporate different aspects of the changing ocean. In addition, in the current setting, they are not only statistically independent drivers but also irreplaceable proxies for biological‐physical coupling processes. Each represents a distinct and well‐established control on biological dynamics: temperature governs metabolic and physiological rates (Clarke and Johnston 1999), chlorophyll reflects the realized phytoplankton biomass and thus food availability for higher‐trophic‐level marine species (Falkowski and Kiefer 1985), and mixed layer depth regulates light and nutrient supply (Diehl et al. 2002). Substituting one with another colinear variable would therefore change the underlying mechanism being represented.

Although substantial efforts have already been made to understand the effects of climate change on fishery‐related measures, including but not limited to recruitment (Britten et al. 2016), biomass (Cheung et al. 2022; van Dorst et al. 2019), catch (Barange et al. 2014; Brander 2007; Free et al. 2019), and productivity dynamics, as explored here, coherent conclusions are hard to obtain. Certainly, the key reasons for these varied messages range from the stock‐specific nature of responses to the breadth and methodology of the analysis. Even during the recent period of unprecedented climate change (Burrows et al. 2011), the effects of ocean warming showed considerable variability among stocks. Beyond the above‐mentioned inherently in‐built variability in sensitivity to warming, species interactions play a critical role in shaping stock productivity and population dynamics (Dupont et al. 2024; Durant et al. 2023). Due to insufficient data availability and model limitations, such interactions were difficult to incorporate into this large‐scale comprehensive analysis. However, shifts in trophic relationships—whether due to altered prey–predator dynamics or species invasions under novel climatic regimes—should be given full attention in fine‐scale investigations. As for chlorophyll, it is closely linked to net primary production (NPP), serving as a key proxy for phytoplankton biomass and oceanic productivity. Worth noting is that NPP‐based, energy‐flow formulations have extensively been used to predict fishery production (Brander 2007; Moore et al. 2018; Stock et al. 2017). This long route of scaling‐up might be problematic because of the relatively high initial uncertainty involved, currently seen by the wide 95% confidence interval at the species level for the slope of chlorophyll versus stock productivity. Also, the influence of NPP (and chlorophyll) can be complex to track, as it depends on whether the stock (population) dynamics is driven by bottom‐up or top‐down trophic forcing (Frank et al. 2007). In the former case a positive relationship with productivity is to be expected, whereas in the latter case possibly a negative one. There may also be time‐varying alterations between bottom‐up and top‐down forcing resulting in no clear, overall relationship. In regard to the weak negative effect of mixed layer depth, this result principally aligns with the Critical Depth Hypothesis or the more recent Dilution‐Recoupling Hypothesis (Behrenfeld 2010): an enlarged mixed layer reduces light availability, phytoplankton production and the concentration of early life stages and planktivorous prey, whereas a contracted mixed layer indicates strengthened stratification that restricts nutrient supply from deep waters (Hou et al. 2022).

### 4.3 Stock Productivity Forecasts

Climate‐induced fishery projections have so far emphasised large‐scale redistributions of the global catch potential (Cheung et al. 2010), variations in stock biomass (Cheung et al. 2022; Tittensor et al. 2021) as well as fishery revenues (Lam et al. 2016). High‐latitude regions are generally said to do better due to the well‐known, on‐going poleward range expansion of a series of species, especially for the North Hemisphere, together with the increased primary production (Cheung et al. 2022). In contrast, a reduced status in that regard is projected for tropical areas (Lam et al. 2020). In our study, such latitudinal patterns appeared to be vague statistically. Also, the implications of the undertaken productivity projections were less severe than generally claimed: examples of projected decreases but also increases were noticed regionally. Indeed, we detected a negative directional effect in the global mean productivity proxy and its associated rate, but these figures were numerically small (only a few percent), depending upon the climate scenario. However, it should be noted that although the Area‐aggregated mean productivities suggest weak responses for climate change, the broad confidence intervals indicate substantial uncertainty. Importantly, this apparent stability to climate change does not imply an absence of ecological responses. Instead, species‐specific random slopes revealed strong and often opposing responses among taxa. These contrasting species‐level patterns partially cancel out when aggregated, resulting in weak effects at the Area‐level. Such compensatory dynamics suggest that the system is undergoing restructuring through shifts in species composition, rather than remaining ecologically stable. Consequently, aggregated patterns may mask substantial underlying biological change.

Related to such forward‐looking perspectives, a current ecosystem model‐based study concluded that changes in the exploitable fish biomass by the end of this century would possibly range from −46.1% to +97.8% under SSP5‐8.5 (−22.9% to +37.8% under SSP1‐2.6) across FAO major fishing areas (Blanchard and Novaglio 2024). In comparison, our stock‐productivity‐based projections are much more conservative, indicating a range of −10.5% to +5.4% under SSP5‐8.5 (−3.5% to +1.7% under SSP1‐2.6) (the respective means with data from IPSL‐CM6A‐LR). The reason for this marked difference in the conclusions made seems to do with model complexity, as mentioned above. It should be noted that SSP5‐8.5 highly likely overstates the future need for fossil fuels and thereby the amount of greenhouse gases released into the atmosphere (Burgess et al. 2023; Hausfather and Peters 2020). In addition, by addressing stock productivity, we are targeting an inherent trait showing clear differences among stocks but being reasonably stable within a given stock. Hence, our global forecast conveys an alternative message to risk assessments provided by end‐to‐end ecosystem models that have depicted consistent and remarkable declines (Blanchard and Novaglio 2024).

Identifying winners and losers—in the present context referring to those stocks expected to respectively benefit and suffer from climate change (Ma, Huse, Ono, Nash, et al. 2024)—would help fishery managers prioritizing targets for prospects and concerns (Griffiths et al. 2017; Lam et al. 2016). Whiting stocks in the Northeast Atlantic were assessed as top winners, consistent with the results of a similar, more regional study using different fishery and biophysical data sources (Ma, Huse, Ono, et al. 2024). Some flatfishes, including several stocks of European plaice ( *P. platessa* ), were listed as top losers in the forecast in view of the species being considered to be negatively affected by warming in the hindcast. This perception was supported by a pioneering study reporting a severe decline in its recruitment capacity (Britten et al. 2016). Although our results suggest a near equal balance between winners and losers, with no clear spatial aggregation patterns, there remains a concern that, in certain regions, commercially or ecologically important stocks may be among the projected losers. Such outcomes could potentially significantly affect regional fisheries production and local livelihoods. A notable example is the European plaice stock in the Baltic Sea, primarily targeted by Danish seine vessels (Ern et al. 2025), which ranks among the top losers in the projections. This illustration once more highlights that protection and management strategies should be tailored to regional‐specific and stock‐specific conditions.

### 4.4 Limitations and Implications

Throughout this study we have emphasized that our additional research effort to present an overview of how climate change affects the global productivity of marine stocks is incomplete due to data gaps in certain regions of the world's oceans. Also, ideally, some of the studied Areas should have been represented even better by including more stocks. For instance, this remark applies to the Northwest Pacific (Area 61)—a region characterized by substantial capture production (FAO 2024)—due to the unavailability of data from some local waters. Addressing these data limitations will require enhanced cooperation to promote data openness and transparency.

Considering uncertainty is a critical aspect of both hindcast and forecast studies. Despite our best efforts to incorporate as many sources of uncertainty as possible, there is still uncertainty left from different aspects that cannot be included into the modelling framework. One important source is the uncertainty associated with using stock assessment outputs as model inputs (Brooks and Deroba 2015). Accessed products such as total stock biomass, abundance, and spawning stock biomass were used for the productivity hindcasts. However, due to limitations in the RAM Legacy Database, uncertainty associated with these indices was not available and therefore could not be incorporated. Although the use of multiple indices, including those from surveys, helped mitigate potential biases that may arise from reliance on a single index, systematic errors, such as the optimistic estimation of biomass for overfished stocks (Edgar et al. 2024), could still result in biased hindcasts of productivity and subsequently affect the climate‐related analyses. Therefore, close collaboration with stock assessors to better understand data quality, along with conducting corresponding sensitivity analyses, is recommended to increase the robustness and confidence of the results.

In addition to uncertainty arising from the data, the model outputs should be interpreted with caution, as they also carry inherent uncertainties. While previous studies often presented seemingly precise results, our results showed that substantial uncertainty remains, even with large datasets. This uncertainty is predominantly associated with estimating species‐specific responses to biophysical drivers, rather than with the climate change scenarios themselves in the context of forecasts. By highlighting this issue, a more cautious and transparent assessment of biological responses and future projections is conveyed, emphasizing that apparent community (or ecosystem)‐level stability can mask complex underlying species‐level dynamics. Furthermore, although several regions show negative mean changes in stock productivity forecast, the uncertainties retain substantial probability for positive changes. Consequently, even where the net trend appears negative, there remains uncertainty regarding the direction of change. Such uncertainty highlights the challenges of attributing observed changes in stock productivity to a limited suite of biophysical drivers. More technically, our findings emphasize the importance of considering the full confidence intervals rather than relying solely on mean values, as doing so provides a more nuanced understanding of potential stock productivity responses to climate change.

A side issue is the largely unknown role of acclimatization and/or adaptation under climate change. Due to the limited quantitative information available in the literature on these physiological topics, such considerations were left out, including distribution shifts (Perry et al. 2005; Poloczanska et al. 2013). Logically, the gain or loss of habitats will influence the stock's capability to adapt over time, but marine reserves (Roberts et al. 2017) or natural refuges (Karnauskas and Cohen 2012) may increase resilience to warming. Such change in resilience could disrupt the numerous climate‐biology relationships we have tested and built, and potentially in some cases bias the projections of the empirical models. With the increasing availability of stock monitoring and climatology data, it is becoming more common to determine (using observational data) or estimate (by simulations) stock distributional shifts. The logical next step will be to incorporate these insights into one‐year‐step iterations, along with the integration of abundance modules.

With the imminent goal of rebuilding global fisheries (Cheung et al. 2022; Worm et al. 2009), effective fisheries management is particularly important, as regarding the presently shown projected declines in stock productivity, though these negative trends were less severe than often previously reported. Despite this, our study revealed marked drops in productivity in some of the FAO major fishing areas, where also some of the most important commercial stocks are found. Hence, the understanding of stock‐ and species‐specific climate‐induced productivity dynamics are of paramount interest for climate‐adaptive fishery management, where one mitigating strategy might be to increase the utilization of the winners and conserve the losers better.

## 5 Conclusion

Facilitated by global fishery‐related databases and earth system models, we estimated stock productivities and related them by statistical methods to key environmental variables. This routine was implemented to get a firmer grasp on how the climate has already affected fish productivity dynamics and then use this knowledge to forecast the most likely productivity performances under different emission scenarios.

Commencing with the hindcast, we estimate productivity patterns for 710 commercially important fisheries stocks in the world's ocean. This vast effort revealed pronounced stock‐specific and regional differences in productivity. Half of the assessed stocks exhibited significant responses to changes in temperature or chlorophyll (or both). The subsequent productivity projections indicated, however, relatively moderate reductions in the proxy for global productivity. Under the ‘business‐as‐usual’ scenario (SSP5‐8.5) this figure dropped by 0.005 (3.0%) towards the end of this century compared to 0.002 (1.0%) under the ‘sustainability’ scenario (SSP1‐2.6). Thus, no widespread, clear‐cut negative effect of climate change on global fisheries as such was found, though it was noticed that some stocks are predicted to do exceedingly poorly (so becoming top losers). The numbers of climate winners and losers remained at a balanced level of about 1:1, essentially explaining the outcome of this unexpected forecast.

The breadth of this analysis outlining the observed and projected productivity of about 700 harvested marine stocks is unmatched in previous research. By paying special attention to measures of stock productivity, the findings should be pertinent in on‐going discussions about the probable situation for these resources in the decades to come. Our research indicates relatively moderate effects of climate change on global fisheries productivity, though with the existence of clear winners and losers. This conclusion contrasts with previous investigations that depict remarkable declines in future fishery landings, though the overall ambition by humans to strive for a low emission scenario remains a significant goal.

## Author Contributions

Shuyang Ma: conceptualization, formal analysis, methodology, software, visualization, writing – original draft, writing – review and editing. **Geir Huse:** funding acquisition, writing – review and editing. **Kotaro Ono:** resources, writing – review and editing. **Maud Alix:** visualization, writing – review and editing. **Yongjun Tian:** writing – review and editing. **Paul J. B. Hart:** writing – review and editing. **Olav Sigurd Kjesbu:** conceptualization, funding acquisition, project administration, supervision, writing – original draft, writing – review and editing.

## Conflicts of Interest

The authors declare no conflicts of interest.

## Supporting information


**Figure S1:** Threshold selection simulation experiment under different coefficient of variations (CVs) and posterior‐to‐prior mean and variance ratios (PPs) levels. The red and blue lines denote the prior and posterior distributions, respectively. Panels with grey backgrounds present the thresholds selected under the corresponding CV and PP levels. Additional details are provided in the Supplementary texts.
**Figure S2:** Scatter plots of posterior‐to prior mean ratio (PPMR) and posterior‐to‐prior variance ratio (PPVR) in log scale for key parameters in surplus production models (r, K and ᴪ). Red points indicate stocks with potentially not informative posteriors, which largely follow the priors, with the discrimination threshold provided in the Supplementary texts and Figure S1.
**Figure S3:** Overview of the length of the various hindcasted stock productivity time series. Solid line shows the stock coverage–defined as the ratio of number of stocks with available data to the total number of stocks–by year. The blue background displays the period when the proportion of available stock productivity data was greater than 60% (dashed line), used as a criterion in the Dynamic Factor Analysis. The label specifies the FAO major fishing area, the corresponding number of stocks, and the addressed time period (in bold). Map lines delineate study areas and do not necessarily depict accepted national boundaries.
**Figure S4:** Loadings on latent trends of hindcasted stock productivity by FAO major fishing area (Area). Point and horizontal line show mean and 95% confidence interval by stock. Within each Area, the stocks were ordered according to their loadings on Trend 1. Labels give Area code, number of stocks in each Area, and numbers of stocks with significant loadings on each trend. A significant loading was defined when the 95% confidence interval of the loadings did not contain zero. The ‘+’ and ‘−’ indicate positive and negative loadings, respectively. Note that labels on y‐axis were hidden for visualization. Map lines delineate study areas and do not necessarily depict accepted national boundaries.
**Figure S5:** Catch proportion of selected stocks to total catch in each FAO major fishing area (area). Strip colours from light green to dark green indicate the representativeness of each Area. Black tells the Area is not included in the following analysis due to too few stocks and little representativeness (see Supplementary texts).
**Figure S6:** Collinearity in biophysical variables across stocks. Shaded area shows distributions of Pearson correlation coefficient. Point marks the median, whereas the vertical lines represent the 66% and 95% ranges of the correlation coefficients, respectively. Red and pink lines indicate the correlation threshold at a significance level of 0.01 and 0.05 (two‐sided Student's t‐test with effective degrees of freedom equal to N−2, 26), respectively. Numbers indicate the number of stocks (in total 652 stocks) for which biophysical variables were not significantly correlated (p > 0.05). CHL: chlorophyll; DO: dissolved oxygen; MLD: mixed layer depth (thickness); NPP: net primary production; S: salinity; SSH: sea surface height; and T: temperature.
**Figure S7:** General Linear Mixed Model (GLMM) comparisons. A total of eight models were fitted 1000 times, and the Akaike Information Criterion (AIC) and Bayesian Information Criterion (BIC) distribution extracted. The difference of AIC and BIC of the targeting model to the best model (the model with the lowest AIC and BIC) that is, ΔAIC and ΔBIC were calculated in each round and shown here. Points and lines show median and 66% and 95% confidence intervals.
**Figure S8:** Hindcasted stock (species)‐specific effects of temperature (T, shown in panel a) and chlorophyll (CHL, shown in panel b). In maps, the point shows the centroid of the stock distribution (same centroids in both panels), with colour reflecting the respective median slope of T and CHL from the 1000 best GLMMs. In scatter plots, the solid line is the smoothed longitudinal and latitudinal trends (by LOcally Estimated Scatterplot Smoothing, LOESS), whereas the shading represents the corresponding 95% confidence interval. The slope of T is multiplied by 100 for visualization. For model convergence, species were used as the random effect term, hence stocks located in different regions but belonging to the same species share the same slope. Map lines delineate study areas and do not necessarily depict accepted national boundaries.
**Figure S9:** Proportion of the various directional effects (negative, neutral or positive) of forecasted stock productivity (2021–2100) by FAO major fishing area (Area). Bars in each panel indicate the respective proportion given by the three Earth System Models (ESMs) and Shared Socioeconomic Pathways (SSPs). A significant trend is defined when the 95% confidence interval of the slope did not contain zero. N gives the number of stocks (shared by different ESMs) assessed in each Area. Map lines delineate study areas and do not necessarily depict accepted national boundaries.
**Figure S10:** Stock productivity forecasts (2021–2100) by FAO major fishing area under three Shared Socioeconomic Pathways (SSPs) with data from MPI‐ESM1‐2‐LR. Coloured lines show the projected mean stock productivity (solid) and directional effect (dashed) by SSP (p < 0.05, two‐sided Student's t‐test), grouped globally or split by FAO major fishing area (Area). The shading reflects 95% confidence interval of the projected mean values from 1000 GLMMs. The total number of stocks analysed is specified (N). The map insert shows the geographical position of each Area. The vertical, dotted lines are separated by 10 years, cf. x‐axis annotations in the ‘global panel’. Map lines delineate study areas and do not necessarily depict accepted national boundaries.
**Figure S11:** Stock productivity forecasts (2021–2100) by FAO major fishing area under three Shared Socioeconomic Pathways (SSPs) with data from NorESM2‐LM. Coloured lines show the projected mean stock productivity (solid) and directional effect (dashed) by SSP (p < 0.05, two‐sided Student's t‐test), grouped globally or split by FAO major fishing area (Area). The shading reflects 95% confidence interval of the projected mean values from 1000 GLMMs. The total number of stocks analysed is specified (N). The map insert shows the geographical position of each Area. The vertical, dotted lines are separated by 10 years, cf. x‐axis annotations in the ‘global panel’. Map lines delineate study areas and do not necessarily depict accepted national boundaries.
**Figure S12:** Forecasted stock winners and losers in the 2090s under three Shared Socioeconomic Pathways (SSPs). The results are presented in view of the current situation (the 2020s). Each plotted point indicates the mean productivity change for a given stock projected with data from MPI‐ESM1‐2‐LR (a) and NorESM2‐LM (b). The SSP‐resolved, stock‐specific mean productivity change is supplemented with 95% confidence interval (horizontal line, projected mean values from 1000 GLMMs). The Top 5 losers and Top 5 winners under SSP5‐8.5 are listed by their stock ID. The adjacent series of numbers show the total number of stocks belonging to either the winner or loser category—that is, the 95% confidence interval of projected productivity change not containing zero—split by SSP1‐2.6, SSP2‐4.5 and SSP5‐8.5. The projected productivity changes under emission scenario SSP5‐8.5 along the y‐axis are sorted by increasing values but hidden for visualization. The applied definition of winner and loser were according to standard practice (see Introduction); the former and latter refer to a stock projected to exhibit increased and decreased productivity, respectively.
**Figure S13:** Distributions of winners and losers. The colour indicates forecasted stock productivity change in the 2090s with reference to the 2020s, projected under three Shared Socioeconomic Pathways (SSPs) SSP1‐2.6 (a), SSP2‐4.5 (b) and SSP5‐8.5 (c). Map lines delineate study areas and do not necessarily depict accepted national boundaries.
**Table S1:** Productivity means and 95% confidence intervals of FAO major fishing area (a), major taxa (b) and family (c).
**Table S3:** Dynamic Factor Analysis (DFA) model comparisons. AICc, sample‐size‐corrected Akaike Information Criterion; logLik, log‐likelihood. Bold figures show the best DFA model for each FAO major fishing area (area).
**Table S4:** Projected global productivity change in the 2090s compared to the 2020s. ESM, Earth System Model; SSP, Shared Socioeconomic Pathways. Productivity change and percentage were calculated with mean productivity in the 2090s as the focus and in the 2020s as the reference.
**Table S5:** Projected FAO major fishing area (Area) productivity change in the 2090s compared to the 2020s. ESM, Earth System Model; SSP, Shared Socioeconomic Pathways. Productivity change and percentage were calculated with mean productivity in the 2090s as the focus and in the 2020s as the reference.

**Table S2:** Stock‐specific details about basic information, surplus production model, productivity hindcast, climate change effects and productivity forecast. Forecast results were based on data from IPSL‐CM6A‐LR.

## Data Availability

Biological data underpinning stock productivity hindcast can be assessed from the RAM Legacy Stock Assessment Database at https://www.ramlegacy.org/, FishBase at https://www.fishbase.se/search.php and SeaLifeBase at https://www.sealifebase.se/search.php. Stock geographical distributions are available from the Global Record of Stocks and Fisheries (GRSF) database at https://i‐marine.d4science.org/web/grsf/home. Biophysical hindcasts can be downloaded from the Global Ocean Ensemble Physics Reanalysis at https://data.marine.copernicus.eu/product/GLOBAL_MULTIYEAR_PHY_ENS_001_031/services and the Global Ocean Biogeochemistry Hindcast at https://data.marine.copernicus.eu/product/GLOBAL_MULTIYEAR_BGC_001_029/services. Biophysical forecasts can be assessed from the Earth System Grid Federation at https://esgf‐data.dkrz.de/search/cmip6‐dkrz/. All analyses are conducted in R with the applied scripts available on GitHub at https://github.com/shuyangma1992/Global_productivity and https://doi.org/10.5281/zenodo.18739838.
