# Supplementary material for: Global Marine Fishery Stock Productivity Under Climate Change
Source: Glob Chang Biol. 2026 Mar 9;32(3):e70784. doi: 10.1111/gcb.70784 (PMC12970580; doi:10.1111/gcb.70784)
Supplement: Supplementary file 1 — Figure S1: Threshold selection simulation experiment under different coefficient of variations (CVs) and posterior‐to‐prior mean and variance ratios (PPs) levels. The red and blue lines denote the prior and posterior distributions, respectively. Panels with grey backgrounds present the thresholds selected under the corresponding CV and PP levels. Additional details are provided in the Supplementary texts. Figure S2: Scatter plots of posterior‐to prior mean ratio (PPMR) and posterior‐to‐prior variance ratio (PPVR) in log scale for key parameters in surplus production models (r, K and ᴪ). Red points indicate stocks with potentially not informative posteriors, which largely follow the priors, with the discrimination threshold provided in the Supplementary texts and Figure S1. Figure S3: Overview of the length of the various hindcasted stock productivity time series. Solid line shows the stock coverage–defined as the ratio of number of stocks with available data to the total number of stocks–by year. The blue background displays the period when the proportion of available stock productivity data was greater than 60% (dashed line), used as a criterion in the Dynamic Factor Analysis. The label specifies the FAO major fishing area, the corresponding number of stocks, and the addressed time period (in bold). Map lines delineate study areas and do not necessarily depict accepted national boundaries. Figure S4: Loadings on latent trends of hindcasted stock productivity by FAO major fishing area (Area). Point and horizontal line show mean and 95% confidence interval by stock. Within each Area, the stocks were ordered according to their loadings on Trend 1. Labels give Area code, number of stocks in each Area, and numbers of stocks with significant loadings on each trend. A significant loading was defined when the 95% confidence interval of the loadings did not contain zero. The ‘+’ and ‘−’ indicate positive and negative loadings, respectively. Note that labels on y‐axis were h [file GCB-32-e70784-s001.docx]

Supplementary Materials for

**Global marine fishery stock productivity under climate change**

Shuyang Ma et al.

*Corresponding author. Email: [olav.kjesbu@hi.no](mailto:olav.kjesbu@hi.no)

**This PDF file includes:**

Supplementary texts

Figures S1 to S13

Tables S1 to S5

**Supplementary Texts**

**Simulation experiments for threshold identification**

To determine whether the Bayesian surplus production model fitting results were prior-driven (i.e. largely influenced by the priors) or data-driven (i.e., primarily informed by the data), we examined the posterior-to-prior mean and variance ratio (PPMR and PPVR) of *r*, *K* and *ᴪ*. Our hypothesis was as follows: if the posterior distributions of parameters closely followed their prior distributions, shown as PPMR and PPVR being close to 1, it indicated the data were uninformative and the results largely depended on the prior distributions. Oppositely, if the posterior distributions of parameters deviated from their prior distributions, shown as PPMR and PPVR keeping a certain distance to 1, i.e. the threshold, it implied that the data were informative and the results mainly depended on the data. Therefore, selecting an appropriate threshold to quantify how large a deviation from 1 is required to distinguish prior and posterior distributions was critical.

Threshold selection was guided by visualization of the prior and posterior distributions. Preliminary analysis indicated that the differences between prior and posterior distributions depended not only on PPMR and PPVR but also on the dispersion of priors, measured by the coefficient of variation (CV). Specifically, for priors with a small CV, even small changes in PPMR and PPVR could produce clearly distinguishable prior and posterior distributions, whereas priors with a large CV required larger changes in PPMR and PPVR to observe a noticeable difference. To account for these effects, we conducted a simulation experiment to identify CV-specific thresholds for PPMR and PPVR.

In the simulation, we first generated prior distributions. The mean of the prior distribution was set to 100 (original scale):

$${Mean}_{prior}=100$$

Preliminary exploration indicated that the choice of prior mean had negligible effects on the results. The standard deviation (log-scale, SD) was calculated as:

$${SD}_{prior}=\sqrt{ln({{CV}_{prior}}^{2}+1)}$$

where CV_prior_ was the coefficient of variation of prior distribution, which was a simulation input parameter (more details shown below).

Next, we calculated the posterior distribution mean and SD based on pre-specified PPMR and PPVR. The mean of the posterior distribution (original scale) was calculated straightforwardly as:

$${Mean}_{posterior}={Mean}_{prior}\times PPMR=100\times(1-PP)$$

where PPMR was the posterior-to-prior mean ratio, represented as 1-PP, where the PP is the change of PPMR to 1 and was a simulation input parameter (more details shown below). The posterior standard deviation was calculated as:

$${SD}_{posterior}=\sqrt{PPVR}\times\frac{{SD}_{prior}}{\ln\left( {Mean}_{prior} \right)}\times\ln\left( {Mean}_{posterior} \right)=\sqrt{1-PP}\times\frac{{SD}_{prior}}{\ln\left( {Mean}_{prior} \right)}\times\ln\left( {Mean}_{posterior} \right)$$

Where PPVR was the posterior-to-prior variance ratio and was again represented as 1-PP, indicating that we assumed equal magnitude of change for PPMR and PPVR in the simulation.

To reflect the real data, we used 10 CV levels: 0.10, 0.25, 0.50, 0.75, 1.00, 1.25, 1.50, 2.00, 3.00 and 4.00. Besides, we considered nine levels of PPMR and PPVR changes (PP): 0.01, 0.02, 0.03, 0.04, 0.05, 0.075, 0.10, 0.15, and 0.20. For each combination of CV and PP, prior and posterior distributions were simulated, and thresholds were determined based on visualization of the overlap between the distributions.

**Area-specific detailed dynamics of stock productivity hindcasts and forecasts**

The following sections detail the dynamics of stock productivity hindcasts and forecasts across each FAO major fishing area (Area), with reference to the number of stocks analysed, data representativeness, linear trends, directional effects and shared dynamic patterns. These results are supported by Figures 4, 5, 7, S3, S4, S5 and S9, and Table S5.

In the hindcast, Trends 1 and 2 refer to outputs from the Dynamic Factor Analysis (DFA) used to identify the common temporal patterns. The loadings associated with each trend indicate the degree to which the productivity patterns of individual stocks correspond to the identified trends, i.e., a positive loading signifies that the stock's productivity was aligned with the trend, whereas a negative loading indicates an inverse pattern. In the forecast, directional effects represent the linear trends in the projected stock productivity, based on simulations from three Earth System Models (ESMs) under three Shared Socioeconomic Pathways (SSPs). In the following heading, representativeness in each Area is evaluated based on the catch proportion of selected stocks to the total catch: no data; 0-25%, poor; 25%-50%, weak; 50-75%, good;, 75%-100%, strong. To ease readability, Area is now and then replaced with ‘region’ or ‘regionally’. All applied and subsequently estimated information on the 710 stocks presently considered in a global perspective are listed and detailed in Table S2.

*Arctic Sea, Area 18*

This Area was not a part of the current study (zero stock inventories).

*Northwest Atlantic, Area 21*

A total of 136 stocks were included in the hindcast analysis, representing approximately 50% to 75% of the total catch in this region (Figure S4), thus providing a good representation of the regional conditions. More than half (54%) of stocks did not demonstrate any discernible linear trends in their hindcasted productivity, while 16% showed positive trends and 30% negative trends (Figure 4). The period selected for the DFA was 1974-2015, during which data was available for over 60% of the stocks (Figure S3). Trend 1 captured 56% of the stocks overall, with 28% showing positive loadings and 28% showing negative loadings (Figure S4). This trend featured a long-term decline, including a sharp increase during the 1990s (Figure 5), suggesting that the majority of stocks in this region experienced declining productivity. Trend 2 captured 42% of the stocks, with more stock holding positive loadings (Figure S4). It exhibited a concave trajectory with a peak in the 1990s (Figure 5), indicating a pattern of multidecadal-scale variability.

For the forecast analysis, a total of 94 stocks produced successful model projections under all three ESMs for the period 2021-2100 (Figure S9). Differences in directional effects among the ESMs were minimal, indicating a high level of consistency across ESMs (Figure S9). Similarly, directional effects were consistent across different SSPs, with 9% of stocks projected to experience increasing productivity and 20% showing decreasing productivity (Figure S9). When aggregating productivity forecasts across stocks with data from IPSL-CM6A-LR—a total of 120 stocks—the results revealed declining trends under all SSPs (Figure 7). The most pronounced decline occurred under SSP5-8.5, with a projected decrease exceeding 0.002 units by the end of the century (Table S5).

*Northeast Atlantic, Area 27*

A total of 146 stocks were included in the hindcast analysis—the highest number among all regions in this study—accounting for more than 75% of the total catch in this region (Figure S5). This status provides a strong representation of the regional conditions. Regarding the linear trends in hindcasted productivity, more than half (53%) of stocks did not demonstrate any discernible trends, while 15% and 32% of stocks showed positive and negative trends, respectively (Figure 4). The period selected for the DFA was 1983-2019, during which data were available for over 60% of the stocks (Figure S3). Trend 1 captured 37% of the stocks overall, with 29% showing positive loadings and 8% displaying negative loadings (Figure S4). This trend featured a long-term decline, with a pronounced decrease from 1990 to 2010 (Figure 5), suggesting that about one third of stocks in this region experienced declining productivity. Trend 2 accounted for 65% of the stocks, with 36% showing positive loadings and 29% negative loadings (Figure S4). In this case a convex trajectory was noticed, peaking in the late 2000s to early 2010s (Figure 5), indicative of a multidecadal-scale variability pattern.

For the forecast analysis, a total of 139 stocks yielded successful model projections under all three ESMs for the period 2021-2100. Differences in directional effects among the ESMs were minimal, indicating a high level of consistency across ESMs (Figure S9). In addition, the directional effects were consistent under different SSPs, with 15% of stocks projected to exhibit increasing and 22% decreasing productivity (Figure S9). When aggregating productivity forecasts across stocks with data from IPSL-CM6A-LR—a total of 142 stocks—the results revealed declining trends under all SSP scenarios (Figure 7). The steepest decline was observed under SSP5-8.5, with a projected decrease exceeding 0.015 units by the end of the century (Table S5).

*Western Central Atlantic, Area 31*

A total of 42 stocks were included in the hindcast analysis, representing approximately 25% to 50% of the total catch in this region (Figure S5), and thus providing a weak representation of the regional conditions. About half (40%) of the stocks did not demonstrate any discernible linear trends in hindcasted productivity, while 38% and 21% of stocks showed positive and negative linear trends, respectively (Figure 4). The period selected for the DFA was 1965-2013, during which data were available for more than 60% of the stocks (Figure S3). Trend 1 captured 74% of the stocks, with 52% exhibiting positive loadings and 22% negative loadings (Figure S4). This trend was stable in the 1970s, increased sharply in the 1980s, and subsequently decreased from the early 1990s to the late 2000s (Figure 5). Trend 2 captured 71% of the stocks, with about 40% displaying positive loadings (Figure S4). An decreasing pattern occurred in the 1980s, remained relatively stable throughout the 1990s, and increased gradually in the 2000s. Together, these two trends suggest that decadal- and multidecadal-scale variability were the dominant pattern of stock productivity dynamics in this region.

For the forecast analysis, a total of 34 stocks yielded successful model projections under all three ESMs for the period 2021-2100. Differences in directional effects among the ESMs were generally small, but the negative effects under SSP1-2.6 were notably larger for the MPI-ESM1-2-LR (Figure S9). When aggregating productivity forecasts across stocks with data from IPSL-CM6A-LR—a total of 39 stocks—the results revealed weak declining trends under SSP2-4.5 and SSP5-8.5 (Figure 7). The most pronounced decline occurred under SSP5-8.5, with a projected decrease of approximately 0.003 units by the end of the century (Table S5).

*Eastern Central Atlantic, Area 34*

A total of 29 stocks were included in the hindcast analysis, representing approximately 50% of the total catch in this region (Figure S4), thereby providing a relatively good representation of the regional conditions. Approximately one third (31%) of the stocks did not demonstrate discernible linear trends in their hindcasted productivity, while 41% and 28% of stocks showed positive and negative trends, respectively (Figure 4). The period selected for the DFA was 1990-2015, during which data were available for over 60% of the stocks (Figure S3). Trend 1 captured 62% of the stocks overall, with 38% showing positive loadings and 24% negative loadings (Figure S4). This trend had a trough around the mid-2000s (Figure 5), suggesting that about half of the stocks experienced minimum productivity during that period. Trend 2 captured 76% of the stocks, with more stocks having positive loadings than those having negative loadings (Figure S4). A sharp increase happened for Trend 2 in the beginning of the mid-2000s (Figure 5). Together, these trends indicate that decadal-scale variability was the dominant dynamic pattern in this region.

For the forecast analysis, a total of 24 stocks yielded successful model projections under all three ESMs for the period 2021-2100. Differences among the ESMs were minimal, indicating a high level of consistency across ESMs (Figure S9). Directional effects across SSPs were also consistent, with 5% of stocks projected to exhibit increasing productivity and 12% showing decreasing trends (Figure S9). When aggregating productivity forecasts across stocks with data from IPSL-CM6A-LR—a total of 25 stocks—the results revealed a slightly increasing trend under SSP1-2.6, while decreasing trends under SSP2-4.5 and SSP5-8.5 (Figure 7). The most pronounced decline occurred under SSP5-8.5, with a projected decrease of approximately 0.006 units by the end of the century (Table S5).

*Mediterranean and Black Sea, Area 37*

A total of 33 stocks were included in the hindcast analysis, representing approximately 25% to 50% of the total catch in this region (Figure S5), providing a weak representation of the regional conditions. Half of the stocks (45%) did not demonstrate any discernible trends in their hindcasted productivity, while 33% and 21% of stocks showed positive and negative trends, respectively (Figure 4). The period selected for the DFA was 2002-2015, the shortest in the study. During this time window, data were available for over 60% of the stocks (Figure S3). Trend 1 captured 33% of the stocks overall, with 24% showing positive loadings and 9% showing negative loadings (Figure S4). This trend showed an increase in the 2000s (Figure 3). Trend 2 captured 85% of the stocks, with an approximately equal distribution of positive and negative loadings (Figure S4). A trough was seen for Trend 2 in the mid-2000s (Figure 5).

For the forecast analysis, a total of 27 stocks yielded successful model projections under all three ESMs for the period 2021-2100. Differences in directional effects among the ESMs were minimal, indicating a high level of consistency across ESMs (Figure S8). Similarly, directional effects under different SSPs were consistent, with 12% of stocks projected to exhibit increasing productivity and 15% decreasing productivity (Figure S9). When aggregating productivity forecasts across stocks with data from IPSL-CM6A-LR—a total of 27 stocks—the results indicated increasing trends under all SSPs (Figure 7). The most pronounced increase was observed under SSP5-8.5, with a projected rise exceeding 0.007 units by the end of the century (Table S5).

*Southwest Atlantic, Area 41*

A total of 12 stocks were included in the hindcast analysis, representing approximately 25% to 50% of the total catch in this region (Figure S5), and thus providing a weak representation of the regional conditions. More than half (58%) of stocks demonstrated increasing trends in hindcasted productivity, while 25% showed decreasing trends and 17% no trend (Figure 4). The period selected for the DFA was 1987-2018, during which data were available for over 60% of the stocks (Figure S3). Trend 1 captured 67% of the stocks overall, with 50% showing positive loadings and 17% negative loadings (Figure S4). This trend was characterized by high variability in the 1990s followed by a substantial decline beginning in the mid-2000s (Figure 5). Trend 2 captured 33% of the stocks, with 25% and 8% of stocks displaying positive and negative loadings, respectively (Figure S4). This trend showed a general increase until the early 2010s (Figure 5).

For the forecast analysis, all 12 stocks yielded successful model projections under all three ESMs for the period 2021-2100. Differences in directional effects among the ESMs were minimal, indicating a high level of consistency across ESMs (Figure S9). The proportion of negative effects under SSP5-8.5 were relatively lower compared to the other two scenarios. When aggregating productivity forecasts across stocks with data from IPSL-CM6A-LR, the results revealed scenario-specific trends: a declining trend under SSP1-2.6 and increasing trends under SSP2-4.5 and SSP5-8.5 (Figure 7). The projected increase under SSP5-8.5 can be above 0.007 units (Table S5).

*Southeast Atlantic, Area 47*

A total of 19 stocks were included in the hindcast analysis, representing approximately 25% to 50% of the total catch in this region (Figure S5), thus providing a weak representation of the regional conditions. The proportions of stocks demonstrating positive, negative, and no discernible trends in hindcasted productivity were 32%, 32%, and 37%, respectively (Figure 4). The period selected for the DFA was 1950-2018, during which data were available for over 60% of the stocks (Figure S3). Both Trend 1 and Trend 2 captured above 70% of the stocks, with a greater number of stocks displaying positive loadings compared to those with negative loadings (Figure S4). Trend 1 peaked around 1960, followed by a long-term decline interspersed with fluctuations. In contrast, Trend 2 showed a marked increase during the 1960s and remained relatively stable thereafter (Figure 5).

For the forecast analysis, a total of 16 stocks yielded successful model projections under all three ESMs for the period 2021-2100. Differences in directional effects among the ESMs were considerable, with the proportion of negative directional effects being lower based on MPI-ESM1-2-LR compared to the other two ESMs (Figure S9). The projections did not show consistent patterns across SSPs. Aggregated productivity forecasts indicated that all the scenarios supported significantly decreasing trends, where the higher-emission scenario was associated with more pronounced declines in productivity (Figure 7), leading to above 0.012 units decrease (Table S5).

*Antarctic Atlantic, Area 48*

This Area was not a part of the current study (zero stock inventories).

*Northwest Pacific, Area 61*

A total of 42 stocks were included in the hindcast analysis, representing less than 25% of the total catch in this region (Figure S5), providing a poor representation of regional the conditions. Half of the stocks (48%) did not demonstrate any discernible trends in their hindcasted productivity, while 17% and 36% showed positive and negative trends, respectively (Figure 4). The period selected for the DFA was 1976-2012, during which data were available for over 60% of the stocks (Figure S3). Trend 1 captured 57% of the stocks, with 26% showing positive loadings and 31% negative loadings (Figure S4). This trend was characterized by a long-term decreasing pattern (Figure 5). Trend 2 captured 93% of the stocks, with an approximately equal distribution of positive and negative loadings (Figure S4). A peak was seen for this trend in the late 1980s, followed by a sharp decrease to a trough in the late 2000s, and then an increase to another peak around the early 2010s (Figure 5). Together, these trends suggest that multidecadal-scale variability dominated the productivity dynamics pattern in this region.

For the forecast analysis, a total of 41 stocks yielded successful model projections under all three ESMs for the period 2021-2100. Differences in directional effects among the ESMs were minimal, indicating a high level of consistency across ESMs (Figure S9). In addition, directional effects under different SSPs were largely consistent, with 5% of stocks projected to exhibit increasing productivity and 25% showing decreasing productivity (Figure S9). When aggregating productivity forecasts across stocks with data from IPSL-CM6A-LR—a total of 41 stocks—the results revealed declining trends under all SSPs (Figure 7). The most pronounced decline was observed under SSP5-8.5, with a projected decrease exceeding 0.020 units by the end of the century (Table S5).

*Northeast Pacific, Area 67*

A total of 110 stocks were included in the hindcast analysis, the third largest number in this study, representing more than 75% of the total catch in this region (Figure S5). This provides a strong representation of the regional conditions. More than half (60%) of the stocks did not demonstrate any discernible trends in their hindcasted productivity, while 20% and 20% showed positive and negative trends, respectively (Figure 4). The period selected for the DFA was 1973-2015, during which data were available for over 60% of the stocks (Figure S3). Trends 1 and 2 captured 35% and 81% of the stocks overall, respectively, with more positive loadings on Trend 1 and an approximately equal distribution of positive and negative loadings on Trend 2 (Figure S4). Trend 1 showed a long-term increasing pattern throughout the 1980s and 1990s, whereas Trend 2 had a trough during the 1990s (Figure 5).

For the forecast analysis, a total of 92 stocks yielded successful model projections under all three ESMs for the period 2021-2100. Differences in directional effects among the ESMs were relatively small, with data from the NorESM2-LM resulting in less positive proportions and more negative proportions under SSP1-2.6 and SSP2-4.5, suggesting a moderate level of consistency across ESMs (Figure S9). The directional effects under different SSPs were comparable but somewhat inconsistent, with a greater number of stocks exhibiting positive effects than negative ones (Figure S9). When aggregating productivity forecasts across stocks with data from IPSL-CM6A-LR—a total of 102 stocks—the results revealed increasing trends under all SSPs (Figure 7). The most pronounced increase occurred under SSP5-8.5, with a projected change of around 0.004 units by the end of the century (Table S5).

*Western Central Pacific, Area 71*

Only 8 stocks were included in the hindcast analysis, representing less than 25% of the total catch in this region (Figure S5), thereby providing a poor representation of the regional conditions. 38% of the stocks demonstrated positive trends in their hindcasted productivity, while 13% of stocks negative trends (Figure 4). The period selected for the DFA was 1964-2014, during which data were available for over 60% of the stocks (Figure S3). Trend 1 captured 63% of the stocks overall, with all stocks showing positive loadings (Figure S4). This trend featured a long-term increasing pattern (Figure 5). Trend 2 captured 50% of the stocks, with more stocks having positive loadings than negative loadings (Figure S4). An increase was seen in the 1960s, then it decreased slowly from 1970 to 2000, with a sharp decrease in the 2000s (Figure 5).

For the forecast analysis, only 6 stocks yielded successful model projections under all three ESMs for the period 2021-2100. Differences in directional effects among the ESMs were minimal, indicating a high level of consistency across ESMs (Figure S9). In addition, the directional effects under different SSPs were consistent, with around 20% of stocks projected to exhibit increasing productivity and 20% decreasing productivity, while the remainder exhibited no significant directional effect (Figure S9). When aggregating productivity forecasts across stocks with data from IPSL-CM6A-LR—a total of 6 stocks—the results indicated increasing trends under all SSPs (Figure 7). The most pronounced increase was observed under SSP5-8.5, with a projected rise more than 0.003 units by the end of the century (Table S5).

*Eastern Central Pacific, Area 77*

A total of 28 stocks were included in the hindcast analysis, representing approximately 25% to 50% of the total catch in this region (Figure S5), thereby providing a weak representation of the regional conditions. Out of these examined stocks, 39% showed positive trends in their hindcasted productivity, 21% negative trends, and the remaining 39% no discernible trend (Figure 4). The period selected for the DFA was 1950-2015, during which data were available for over 60% of the stocks (Figure S3). Trend 1 captured 89% of the stocks, with 75% exhibiting positive loadings and 14% negative loadings (Figure S4). This trend declined during the 1970s, increased from the mid-1980s to the mid-2000s, and then declined again (Figure 5). Trend 2 captured 86% of the stocks, with 71% and 15% showing positive and negative loadings, respectively (Figure S4). A peak was seen for this trend during the 1980s, that is, at the same time as the trough for Trend 1 (Figure 5). Together, these trends suggest that productivity dynamics in this region were primarily characterized by multidecadal-scale variability.

For the forecast analysis, a total of 25 stocks yielded successful model projections under all three ESMs for the period 2021-2100. While directional effects varied slightly among ESMs and SSPs, more stocks were projected to experience positive than negative effects (Figure S9). When aggregating productivity forecasts across stocks with data from IPSL-CM6A-LR —a total of 26 stocks—the results indicated increasing trends under all SSPs (Figure 7). The most pronounced increase was observed under SSP5-8.5, with a projected rise of about 0.003 units by the end of the century (Table S5).

*Southwest Pacific, Area 81*

A total of 60 stocks were included in the hindcast analysis, representing approximately 50% of the total catch in this region (Figure S5), thereby providing a good representation of regional conditions. Out of these stocks, 60% demonstrated positive trends in their hindcasted productivity, while 13% and 27% negative and no trends, respectively (Figure 4). The period selected for the DFA was 1975-2012, during which data were available for over 60% of the stocks (Figure S3). Trend 1 captured 63% of the stocks, with 60% displaying positive loadings and 3% negative loadings (Figure S4). This trend featured a long-term increase peaking in the mid-2000s (Figure 5), suggesting that the majority of stocks in this region experienced rising productivity. Trend 2 captured 68% of the stocks, with an approximately equal distribution of positive and negative loadings (Figure S4), noticing a peak in the late 1980s to the early 1990s and then a decline toward the late 2000s (Figure 5), indicating a pattern of multidecadal-scale variability.

For the forecast analysis, a total of 59 stocks yielded successful model projections under all three ESMs for the period 2021-2100. Directional effects were generally consistent across ESMs and SSPs, except for a relatively high proportion of negative effects under SSP1-2.6 based on MPI-ESM1-2-LR (Figure S9). Overall, more stocks were projected to experience positive than negative effects, with proportions of around 25% and 10%, respectively (Figure S9). When aggregating productivity forecasts across stocks with data from IPSL-CM6A-LR—a total of 59 stocks—the results revealed increasing trends under all SSPs (Figure 7). The most pronounced increase was projected under SSP5-8.5, with an estimated rise of approximately 0.010 units by the end of the century (Table S5).

*Southeast Pacific, Area 87*

A total of 24 stocks were included in the hindcast analysis, representing approximately 50% to 75% of the total catch in this region (Figure S5), thereby providing a good representation of the regional conditions. The proportions of trends in hindcasted productivity were relatively balanced, with 46% of stocks demonstrating positive trends, 33% negative trends, and 21% no discernible trend (Figure 4). The period selected for the DFA was 1979-2014, during which data were available for over 60% of the stocks (Figure S3). Trend 1 accounted for only 29% of the stocks and showed an roughly even distribution between positive and negative loadings (Figure S4). This trend displayed moderate interannual variability through 2000, followed by a pronounced decline in the 2000s (Figure 5). Trend 2 encompassed 50% of the stocks and exhibited a greater proportion of positive loadings (33%) compared to negative loadings (17%) (Figure S4). This trend was characterized by a relatively stable trajectory initially, transitioning into a marked increase beginning in the early 1990s (Figure 5), suggesting emerging positive productivity signals in a subset of the stocks.

For the forecast analysis, 22 stocks yielded successful model projections under all three ESMs for the period 2021-2100. Substantial variability was observed in the directional effects across different ESMs and SSPs, underscoring model and scenario sensitivity in this region (Figure S9). When aggregating productivity forecasts across stocks with data from IPSL-CM6A-LR, the forecasts exhibited considerable interannual fluctuations. Statistically significant declining trends were detected under all the scenarios, pointing to potential long-term reductions in stock productivity (Figure 7). The most pronounced decrease was projected under SSP5-8.5, with an estimated decline of 0.013 units by the end of the century (Table S5).

*Antarctic Pacific, Area 88*

This Area was not a part of the current study (zero stock inventories).

*Western Indian Ocean, Area 51*

Only 3 stocks were included in the analysis, cancelling thereby out the Area-specific analysis as such.

*Eastern Indian Ocean, Area 57*

A total of 17 stocks were included in the hindcast analysis, representing less than 25% of the total catch in this region (Figure S5), thereby providing a poor representation of the regional conditions. The majority of stocks (59%) demonstrate positive trends in hindcasted productivity, while 12% and 29% showed positive and no trends, respectively (Figure 4). The period selected for the DFA was 1979-2009, during which data were available for over 60% of the stocks (Figure S3). Trend 1 captured 82% of the stocks overall, with 59% showing positive loadings and 23% negative loadings (Figure S4). This trend remained relatively stable prior to 1990, followed by a marked increase in the subsequent years (Figure 5). Trend 2 encompassed 88% of the stocks and was characterized by more positive loadings (53%) than negative loadings (35%) (Figure S4). Trend 2 displayed a rapid decline until around 2000, followed by a sharp increase, indicating a reversal in productivity dynamics (Figure 5).

For the forecast analysis, all 17 stocks yielded successful model projections under all three ESMs for the period 2021-2100. Directional effects varied slightly across ESMs and SSPs, but a higher proportion of stocks exhibited positive effects compared to negative ones (Figure S9). When aggregating productivity forecasts across stocks with data from IPSL-CM6A-LR, increasing trends were observed under all SSPs (Figure 7). The most pronounced increase occurred under SSP5-8.5, with a projected gain exceeding 0.010 units by the end of the century (Table S5).

*Antarctic, Southern Indian Ocean, Area 58*

Only 1 stock was included in the analysis, cancelling thereby out the Area-specific analysis as such.

**Supplementary Figures**


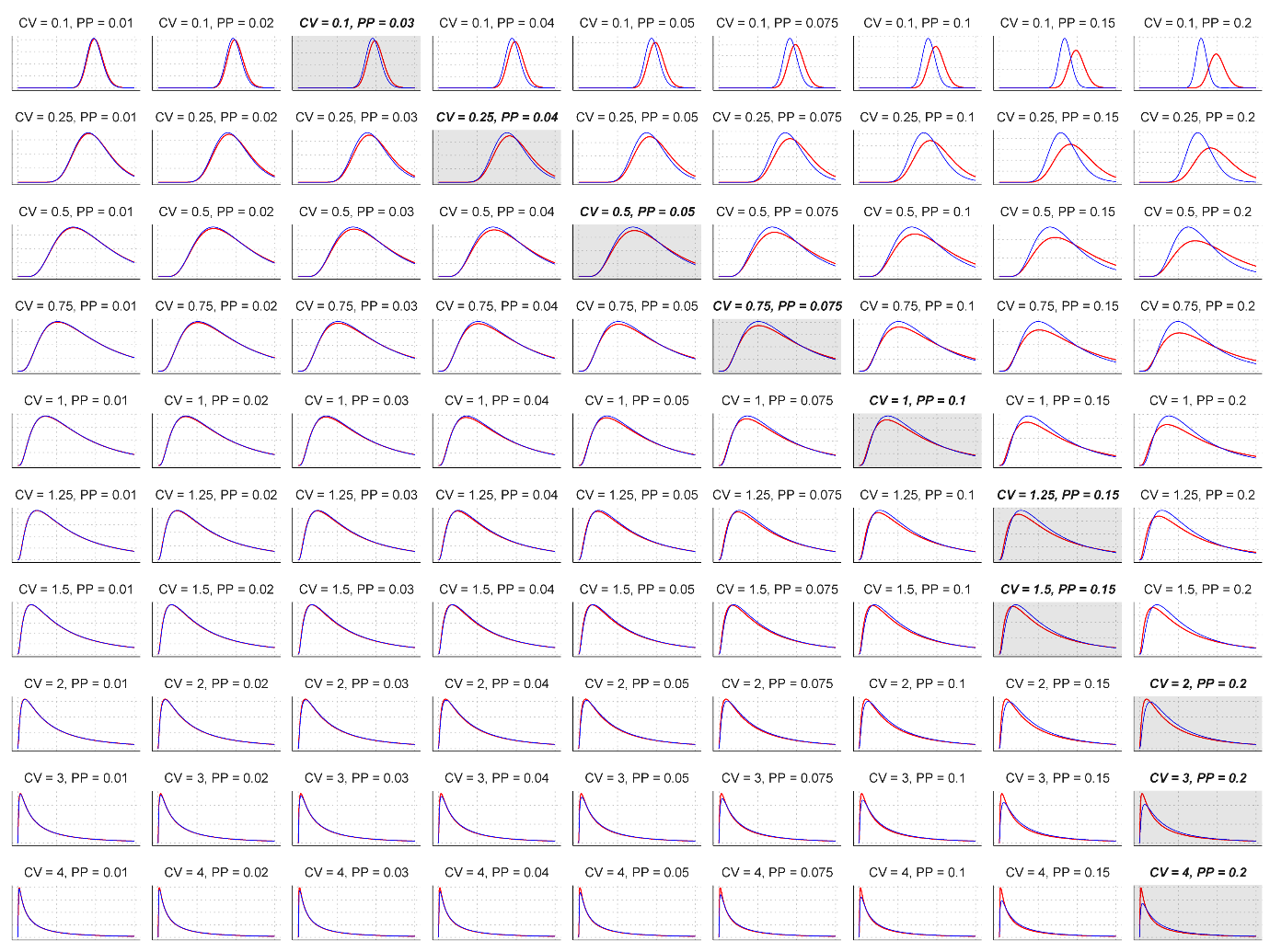


**FIGURE S1 |** Threshold selection simulation experiment under different coefficient of variations (CVs) and posterior-to-prior mean and variance ratios (PPs) levels. The red and blue lines denote the prior and posterior distributions, respectively. Panels with grey backgrounds present the thresholds selected under the corresponding CV and PP levels. Additional details are provided in the Supplementary texts.


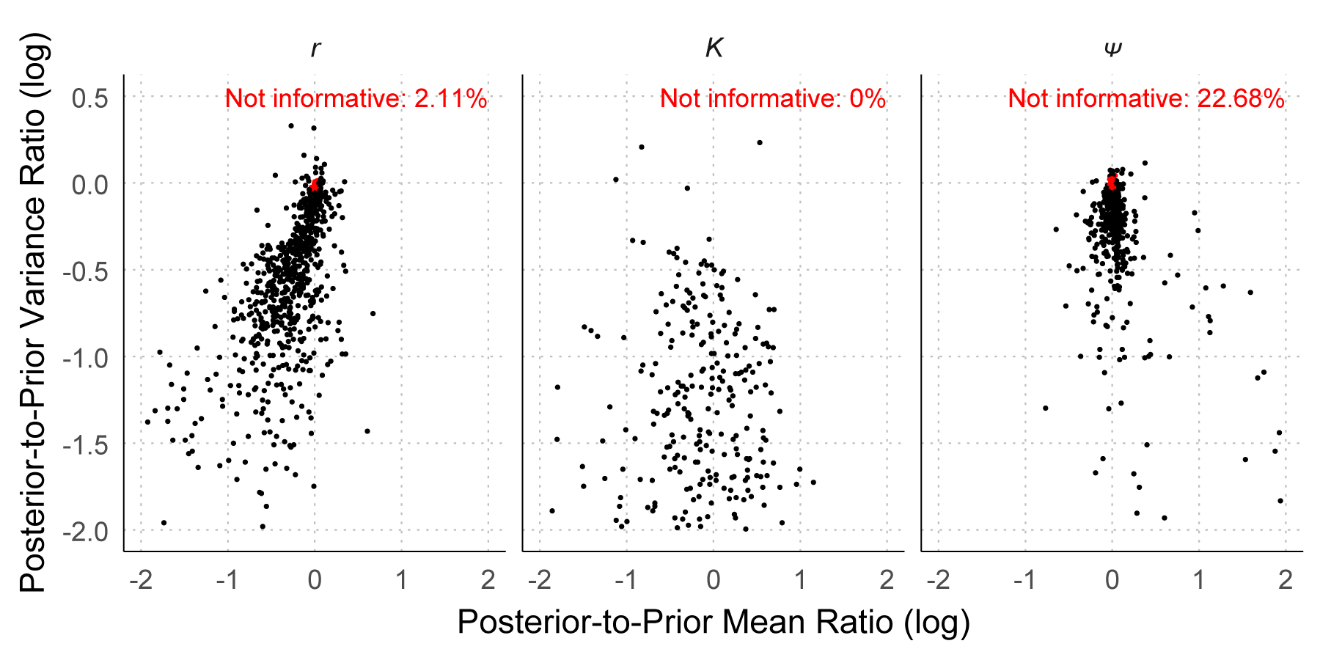


**FIGURE S2 |** Scatter plots of posterior-to prior mean ratio (PPMR) and posterior-to-prior variance ratio (PPVR) in log scale for key parameters in surplus production models (*r*, *K*, and *ᴪ*). Red points indicate stocks with potentially not informative posteriors, which largely follow the priors, with the discrimination threshold provided in the Supplementary texts and Figure S1.


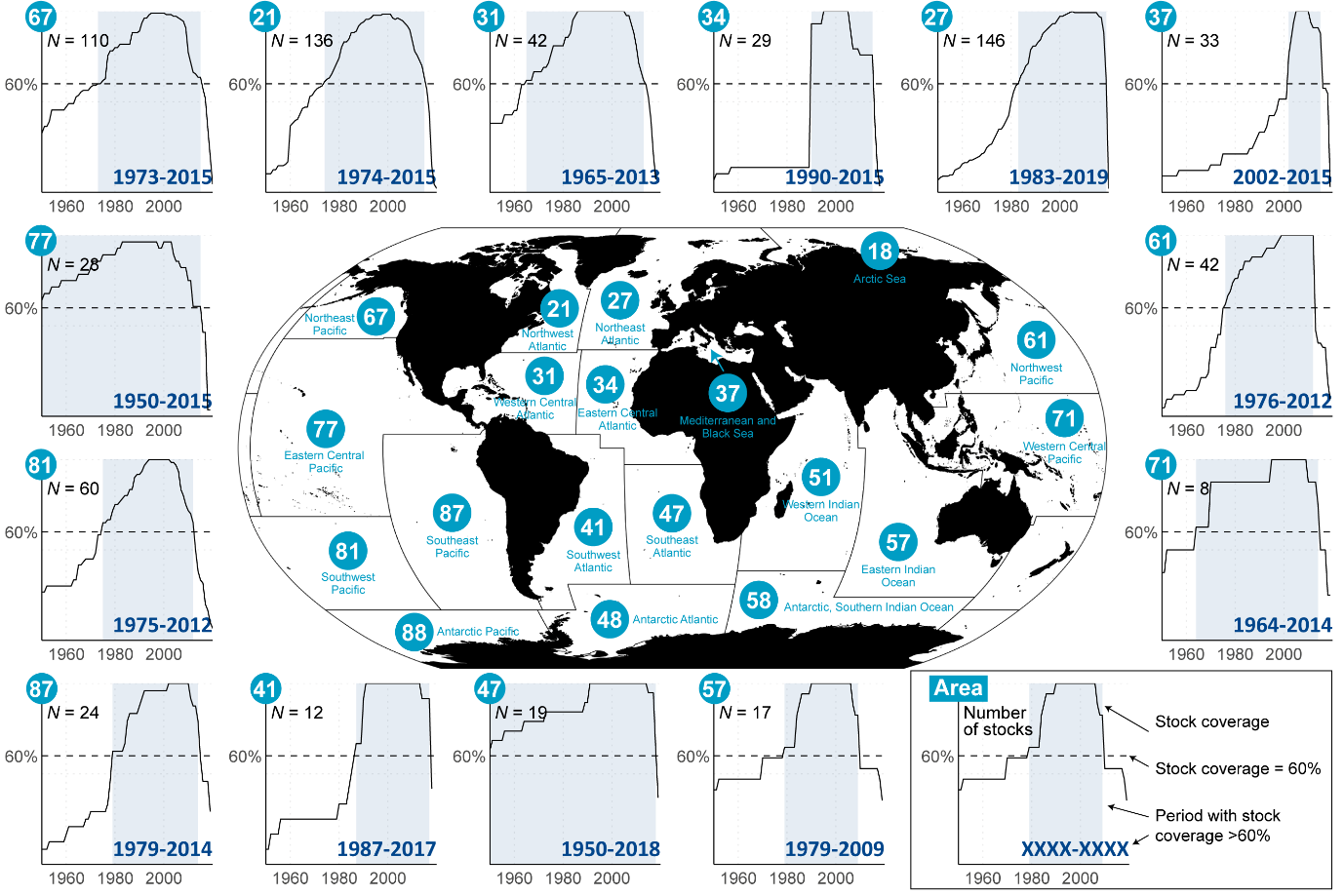


**FIGURE S3 |** Overview of the length of the various hindcasted stock productivity time series. Solid line shows the stock coverage–defined as the ratio of number of stocks with available data to the total number of stocks–by year. The blue background displays the period when the proportion of available stock productivity data was greater than 60% (dashed line), used as a criterion in the Dynamic Factor Analysis. The label specifies the FAO major fishing area, the corresponding number of stocks, and the addressed time period (in bold). Map lines delineate study areas and do not necessarily depict accepted national boundaries.


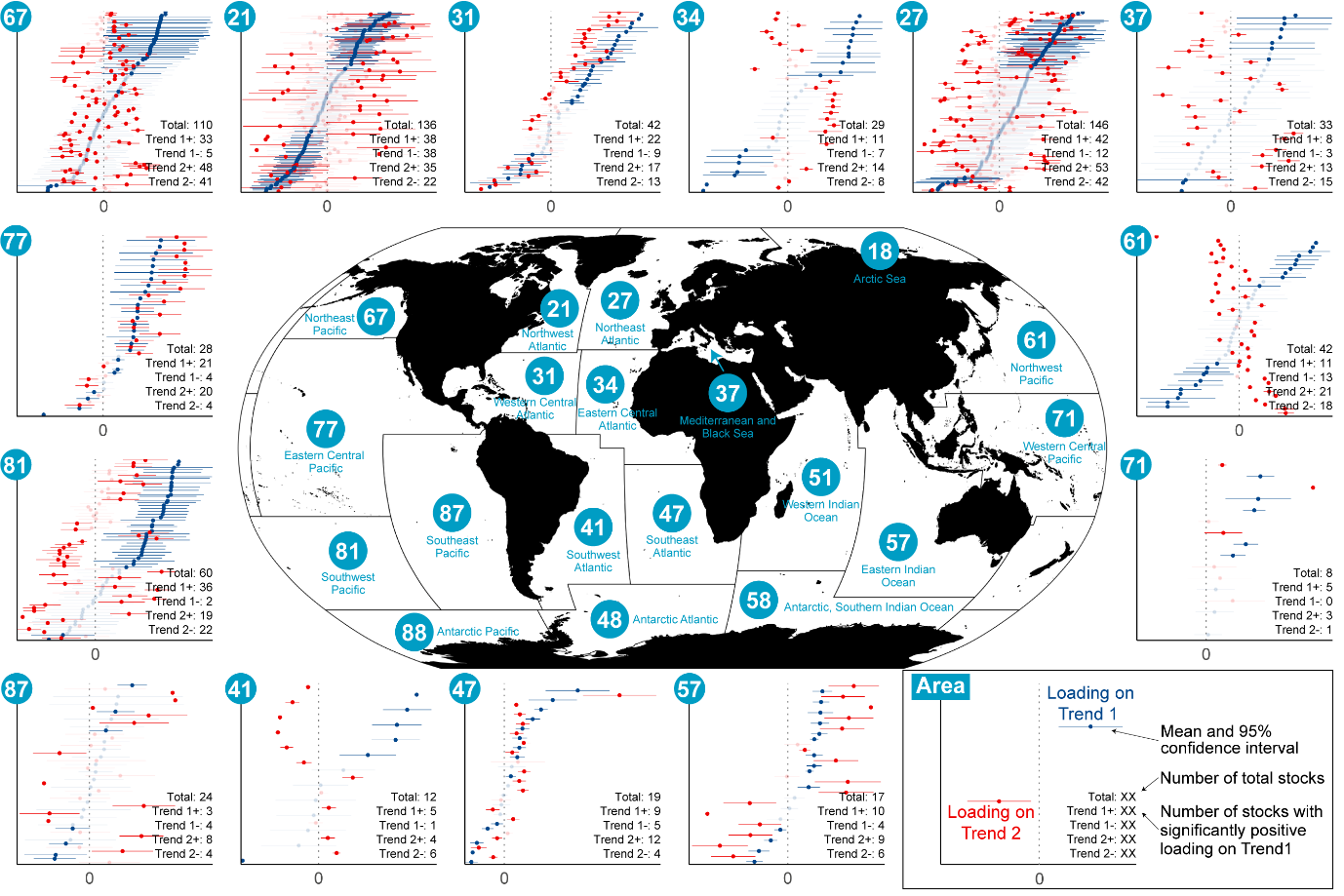


**FIGURE S4 |** Loadings on latent trends of hindcasted stock productivity by FAO major fishing area (Area). Point and horizontal line show mean and 95% confidence interval by stock. Within each Area, the stocks were ordered according to their loadings on Trend 1. Labels give Area code, number of stocks in each Area, and numbers of stocks with significant loadings on each trend. A significant loading was defined when the 95% confidence interval of the loadings did not contain zero. The ‘+’ and ‘-’ indicate positive and negative loadings, respectively. Note that labels on y-axis were hidden for visualization. Map lines delineate study areas and do not necessarily depict accepted national boundaries.


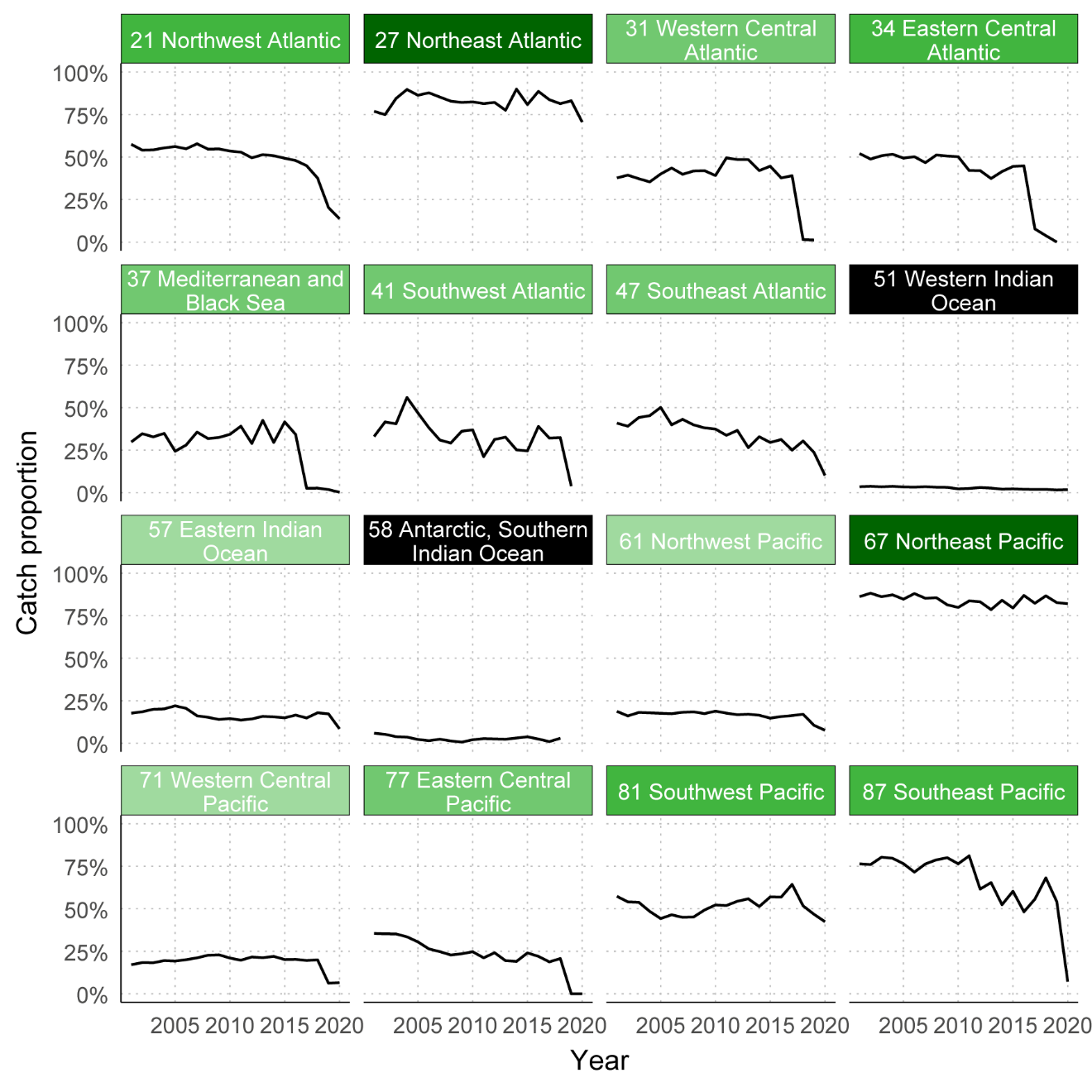


**FIGURE S5** **|** Catch proportion of selected stocks to total catch in each FAO major fishing area (Area). Strip colours from light green to dark green indicate the representativeness of each Area. Black tells the Area is not included in the following analysis due to too few stocks and little representativeness (see Supplementary texts).


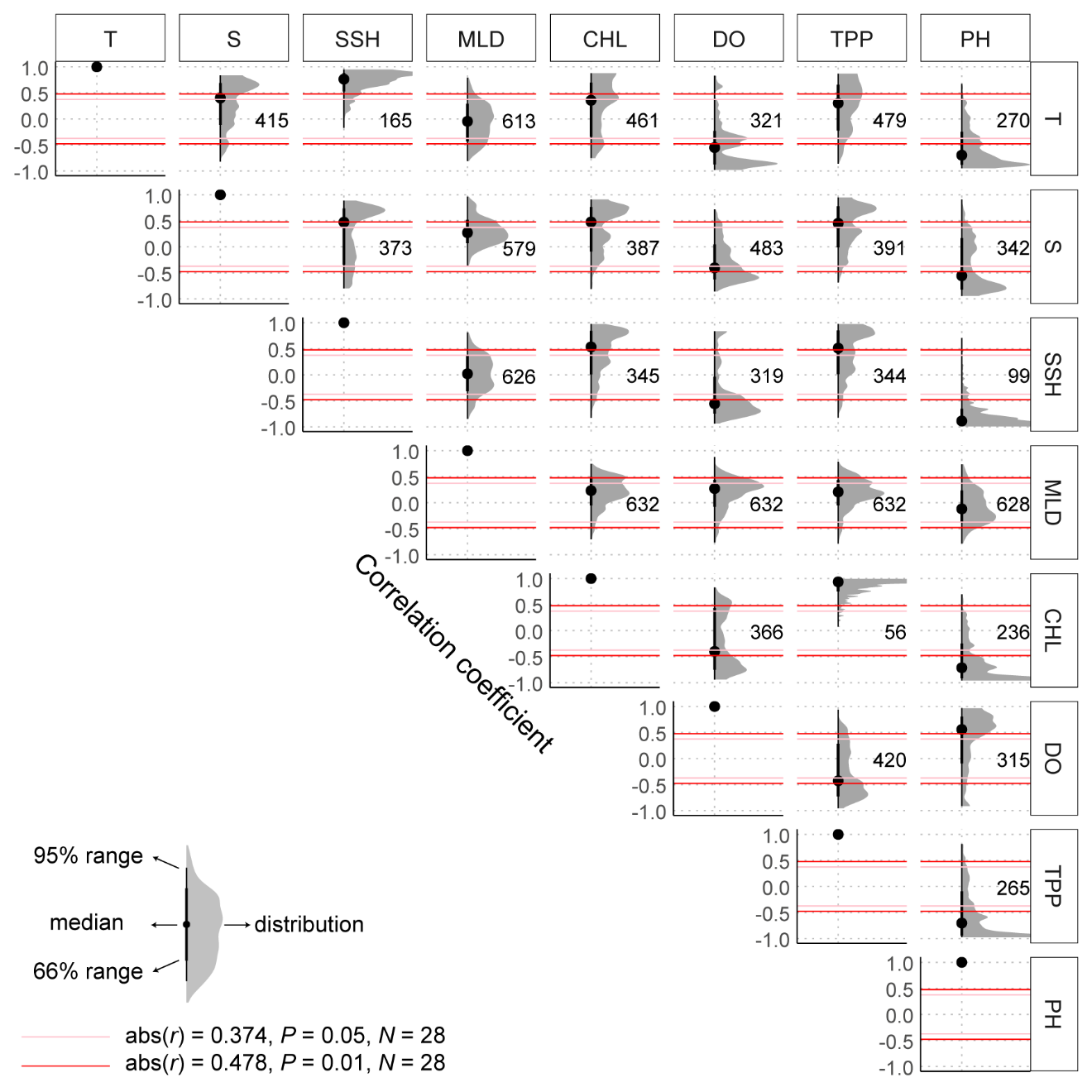


**FIGURE S6 |** Collinearity in biophysical variables across stocks. Shaded area shows distributions of Pearson correlation coefficient. Point marks the median, whereas the vertical lines represent the 66% and 95% ranges of the correlation coefficients, respectively. Red and pink lines indicate the correlation threshold at a significance level of 0.01 and 0.05 (two-sided Student’s t-test with effective degrees of freedom equal to *N*-2, 26), respectively. Numbers indicate the number of stocks (in total 652 stocks) for which biophysical variables were not significantly correlated (*p* > 0.05). T: temperature; S: salinity; SSH: sea surface height; MLD: mixed layer depth (thickness); CHL: chlorophyll; DO: dissolved oxygen; and NPP: net primary production.


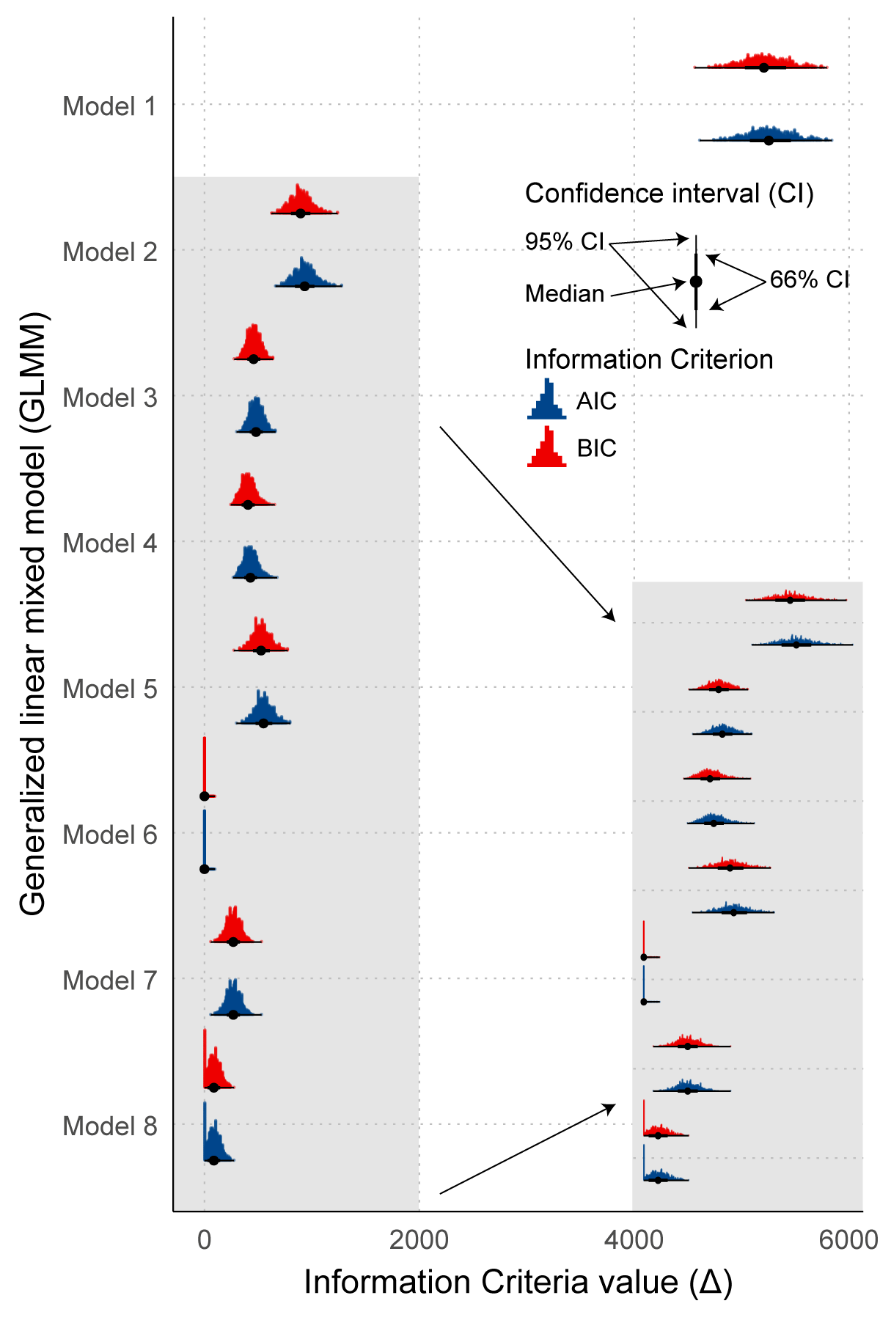


**FIGURE S7 |** General Linear Mixed Model (GLMM) comparisons. A total of eight models were fitted 1,000 times, and the Akaike Information Criterion (AIC) and Bayesian Information Criterion (BIC) distribution extracted. The difference of AIC and BIC of the targeting model to the best model (the model with the lowest AIC and BIC), i.e. ΔAIC and ΔBIC were calculated in each round and shown here. Points and lines show median and 66% and 95% confidence intervals.


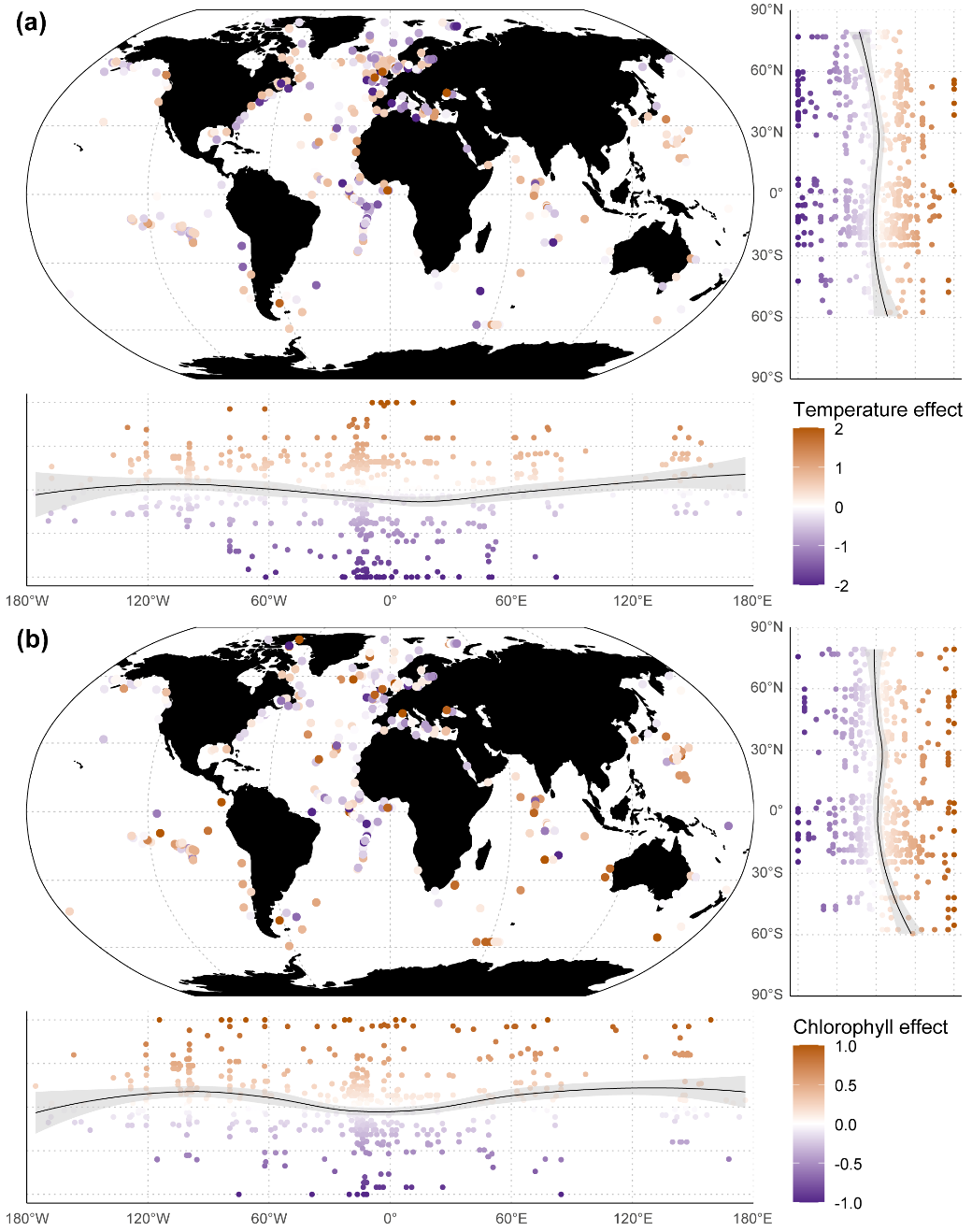


**FIGURE S8 |** Hindcasted stock (species)-specific effects of temperature (T, shown in panel a) and chlorophyll (CHL, shown in panel b). In maps, the point shows the centroid of the stock distribution (same centroids in both panels), with colour reflecting the respective median slope of T and CHL from the 1,000 best GLMMs. In scatter plots, the solid line is the smoothed longitudinal and latitudinal trends (by LOcally Estimated Scatterplot Smoothing, LOESS), whereas the shading represents the corresponding 95% confidence interval. The slope of T is multiplied by 100 for visualization. For model convergence, species were used as the random effect term, hence stocks located in different regions but belonging to the same species share the same slope. Map lines delineate study areas and do not necessarily depict accepted national boundaries.


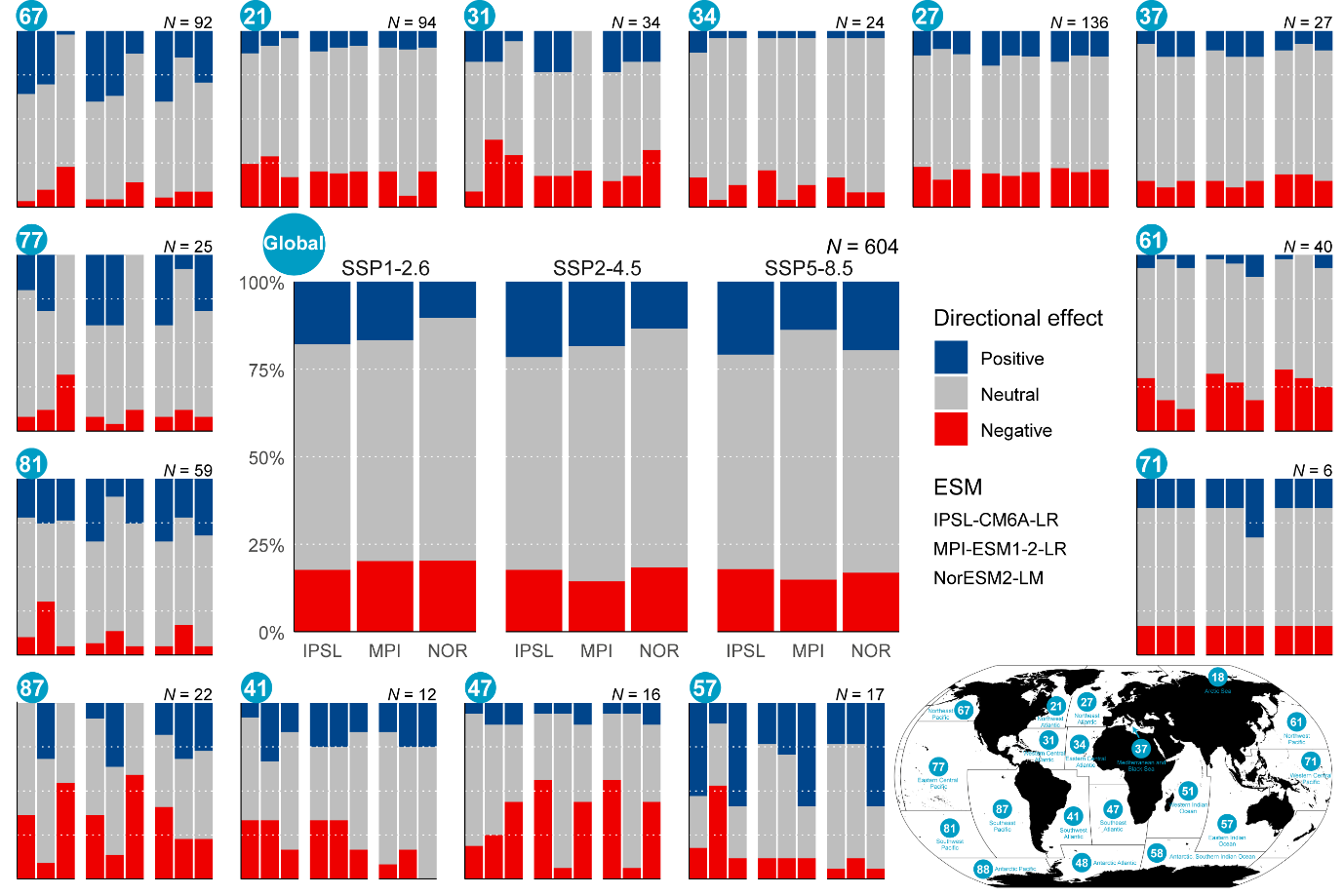


**FIGURE S9 |** Proportion of the various directional effects (negative, neutral or positive) of forecasted stock productivity (2021-2100) by FAO major fishing area (Area). Bars in each panel indicate the respective proportion given by the three Earth System Models (ESMs) and Shared Socioeconomic Pathways (SSPs). A significant trend is defined when the 95% confidence interval of the slope did not contain zero. *N* gives the number of stocks (shared by different ESMs) assessed in each Area. Map lines delineate study areas and do not necessarily depict accepted national boundaries.


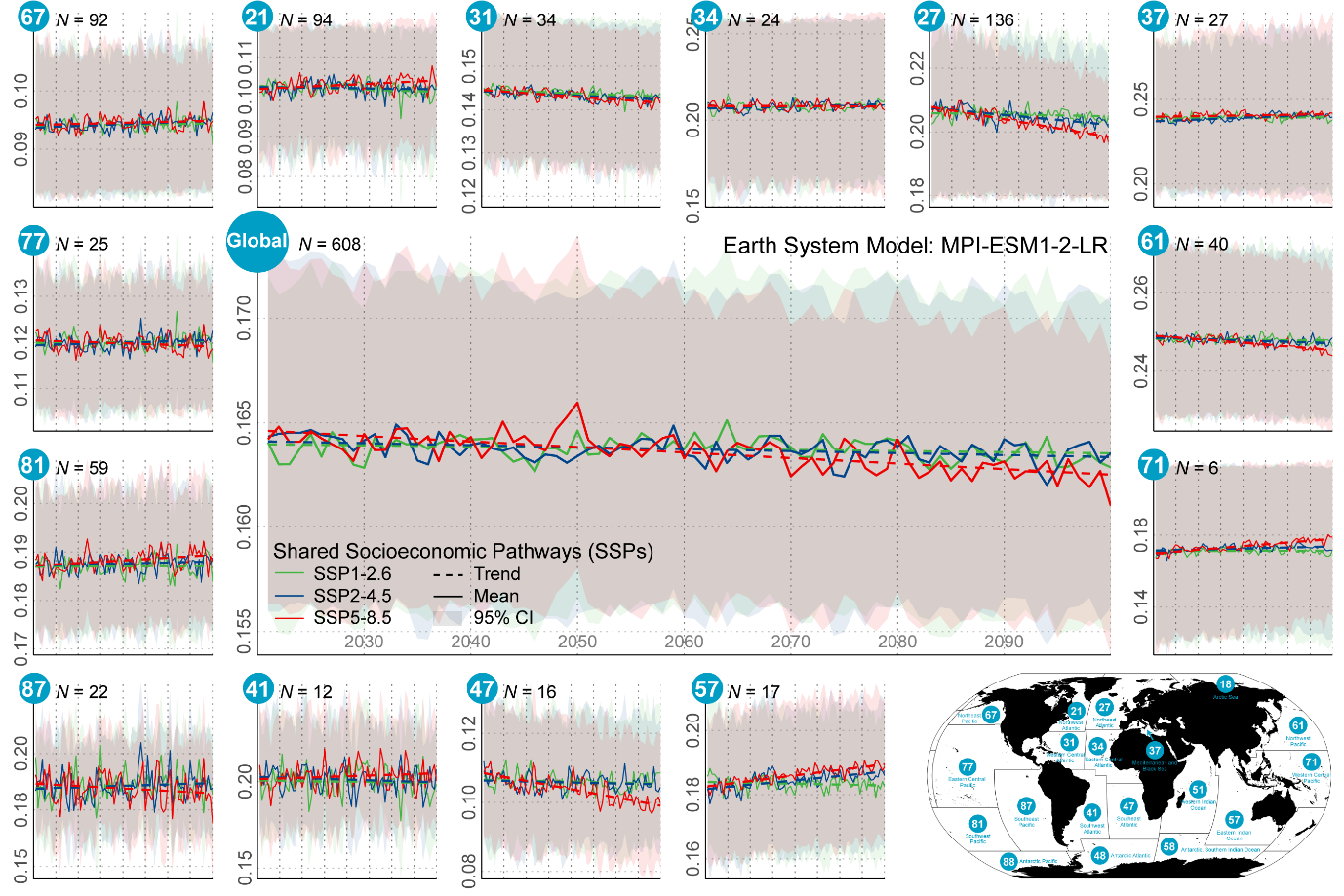


**FIGURE S10** **|** Stock productivity forecasts (2021-2100) by FAO major fishing area under three Shared Socioeconomic Pathways (SSPs) with data from MPI-ESM1-2-LR. Coloured lines show the projected mean stock productivity (solid) and directional effect (dashed) by SSP (*P* < 0.05, two-sided Student’s t-test), grouped globally or split by FAO major fishing area (Area). The shading reflects 95% confidence interval of the projected mean values from 1,000 GLMMs. The total number of stocks analysed is specified (N). The map insert shows the geographical position of each Area. The vertical, dotted lines are separated by 10 years, cf. x-axis annotations in the ‘global panel’. Map lines delineate study areas and do not necessarily depict accepted national boundaries.


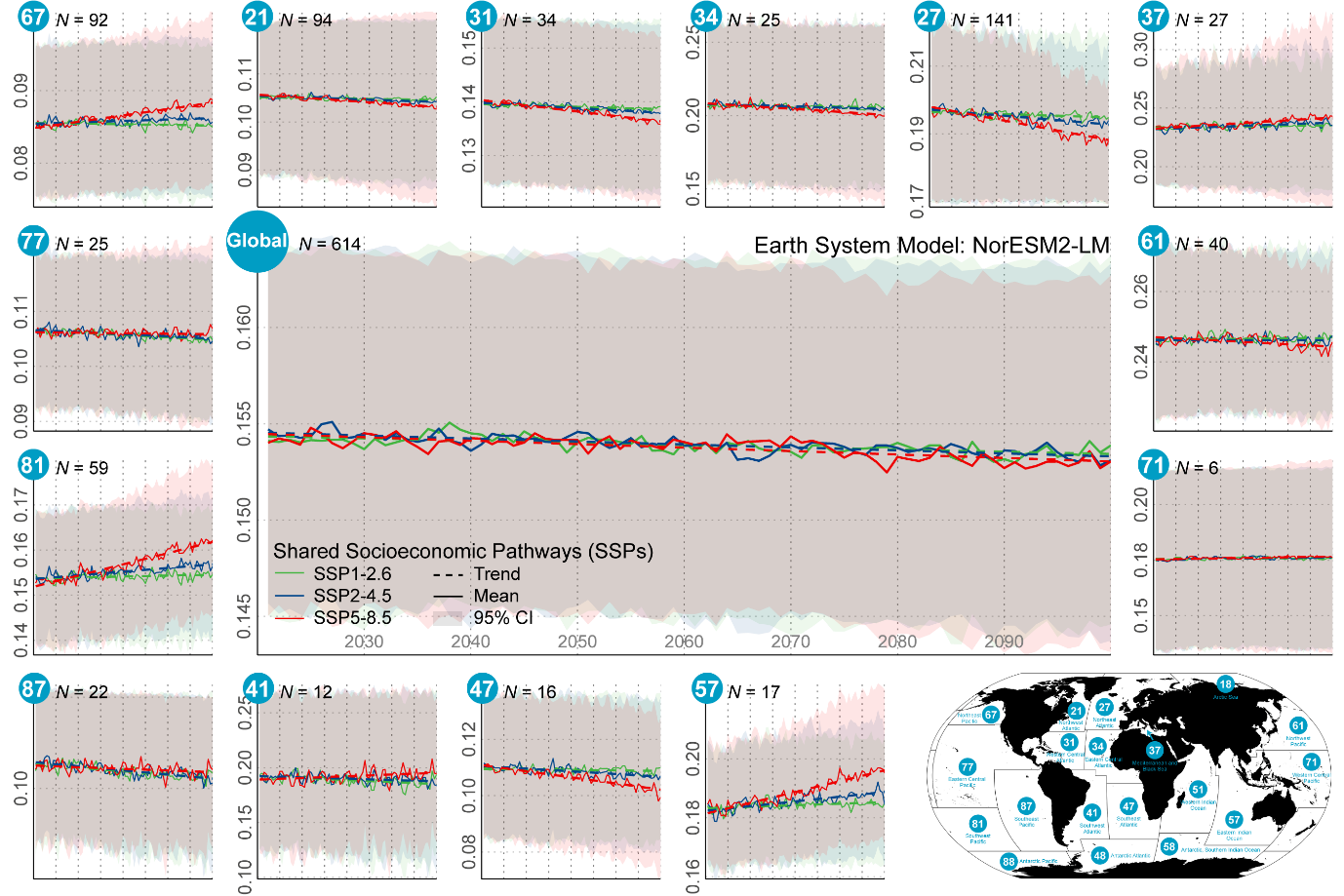


**FIGURE S11** **|** Stock productivity forecasts (2021-2100) by FAO major fishing area under three Shared Socioeconomic Pathways (SSPs) with data from NorESM2-LM. Coloured lines show the projected mean stock productivity (solid) and directional effect (dashed) by SSP (*P* < 0.05, two-sided Student’s t-test), grouped globally or split by FAO major fishing area (Area). The shading reflects 95% confidence interval of the projected mean values from 1,000 GLMMs. The total number of stocks analysed is specified (N). The map insert shows the geographical position of each Area. The vertical, dotted lines are separated by 10 years, cf. x-axis annotations in the ‘global panel’. Map lines delineate study areas and do not necessarily depict accepted national boundaries.


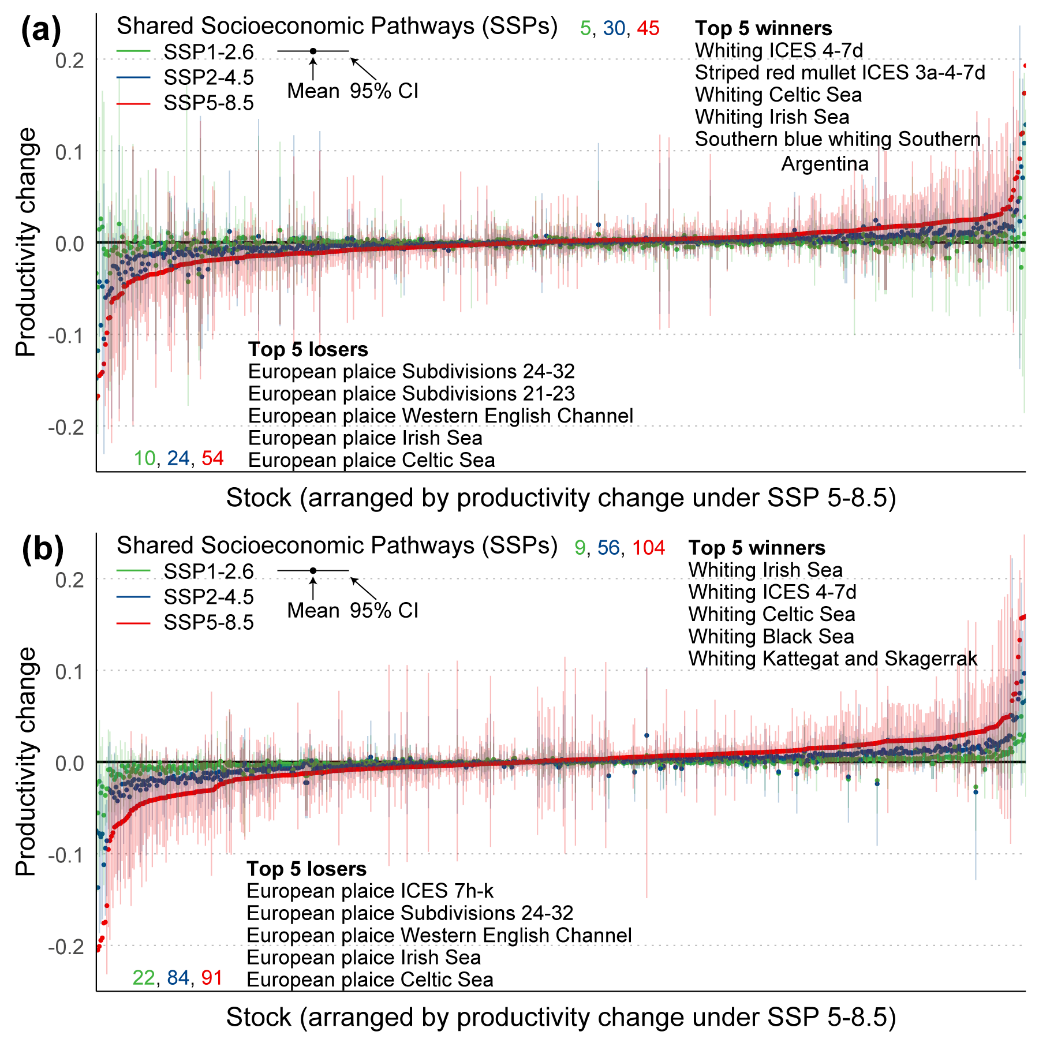


**FIGURE S12** **|** Forecasted stock winners and losers in the 2090s under three Shared Socioeconomic Pathways (SSPs). The results are presented in view of the current situation (the 2020s). Each plotted point indicates the mean productivity change for a given stock projected with data from MPI-ESM1-2-LR (a) and NorESM2-LM (b). The SSP-resolved, stock-specific mean productivity change is supplemented with 95% confidence interval (horizontal line, projected mean values from 1,000 GLMMs). The Top 5 losers and Top 5 winners under SSP5-8.5 are listed by their stock ID. The adjacent series of numbers show the total number of stocks belonging to either the winner or loser category–that is, the 95% confidence interval of projected productivity change not containing zero–split by SSP1-2.6, SSP2-4.5 and SSP5-8.5. The projected productivity changes under emission scenario SSP5-8.5 along the y-axis are sorted by increasing values but hidden for visualization. The applied definition of winner and loser were according to standard practice (see Introduction); the former and latter refer to a stock projected to exhibit increased and decreased productivity, respectively.


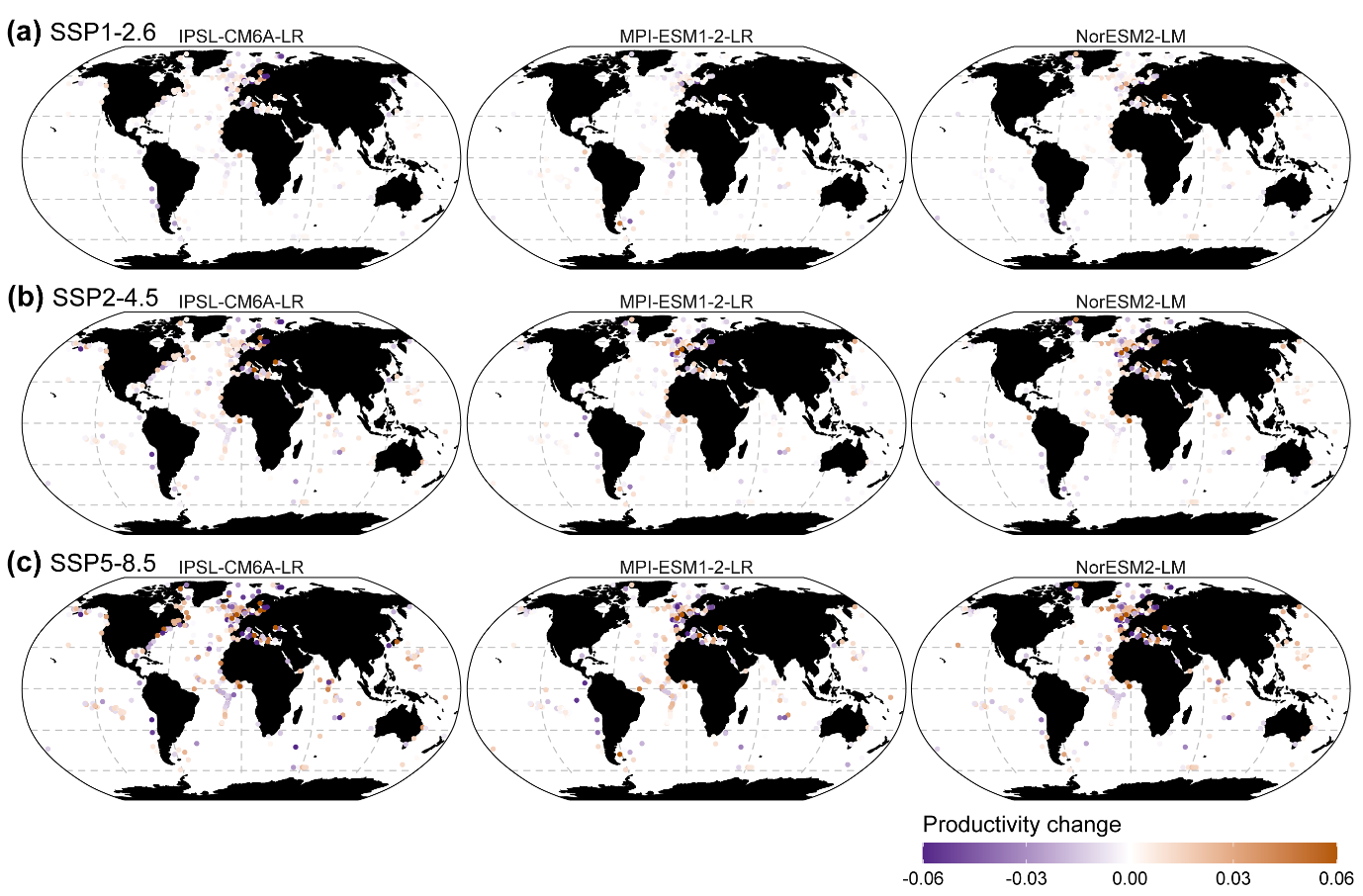


**FIGURE S13 |** Distributions of winners and losers. The colour indicates forecasted stock productivity change in the 2090s with reference to the 2020s, projected under three Shared Socioeconomic Pathways (SSPs) SSP1-2.6 (a), SSP2-4.5 (b) and SSP5-8.5 (c). Map lines delineate study areas and do not necessarily depict accepted national boundaries.

**Supplementary Tables**

**TABLE S1** | Productivity means and 95% confidence intervals of FAO major fishing area (a), major taxa (b) and family (c).

**a** **FAO major fishing area**

| FAO major fishing area | Sample size | Productivity mean (95% confidence interval) |
| --- | --- | --- |
| 37 | 33 | 0.311 (-0.128-0.749) |
| 61 | 42 | 0.285 (-0.065-0.635) |
| 34 | 29 | 0.246 (0.014-0.479) |
| 27 | 146 | 0.210 (-0.076-0.495) |
| 41 | 12 | 0.203 (-0.084-0.490) |
| 87 | 24 | 0.190 (-0.055-0.435) |
| 21 | 136 | 0.138 (-0.074-0.350) |
| 81 | 60 | 0.124 (-0.042-0.289) |
| 47 | 19 | 0.118 (-0.084-0.321) |
| 57 | 17 | 0.108 (-0.079-0.294) |
| 31 | 42 | 0.099 (-0.090-0.288) |
| 71 | 8 | 0.091 (-0.034-0.216) |
| 67 | 110 | 0.075 (-0.073-0.223) |
| 77 | 28 | 0.064 (-0.111-0.238) |

**b Major taxa**

| Major taxa | Sample size | Productivity mean (95% confidence interval) |
| --- | --- | --- |
| Forage Fish | 95 | 0.24 (-0.076-0.557) |
| Gadids | 111 | 0.18 (0.003-0.358) |
| Other Marine | 135 | 0.163 (-0.199-0.526) |
| Invertebrate | 142 | 0.162 (-0.07-0.394) |
| Flatfish | 91 | 0.158 (-0.093-0.408) |
| Tuna and Marlin | 43 | 0.145 (-0.077-0.367) |
| Sharks Rays and Skates | 38 | 0.058 (-0.057-0.174) |
| Rockfish | 55 | 0.033 (-0.048-0.114) |

**c Family**

| Family | Sample size | Productivity mean (95% confidence interval) |
| --- | --- | --- |
| Ommastrephidae | 2 | 0.637 (0.404-0.87) |
| Mullidae | 9 | 0.545 (-0.299-1.389) |
| Dussumieriidae | 1 | 0.524 (NA-NA) |
| Scomberesocidae | 1 | 0.504 (NA-NA) |
| Strongylocentrotidae | 1 | 0.488 (NA-NA) |
| Sciaenidae | 2 | 0.349 (-0.17-0.868) |
| Polynemidae | 2 | 0.34 (-0.034-0.714) |
| Tetraodontidae | 1 | 0.324 (NA-NA) |
| Engraulidae | 18 | 0.32 (-0.102-0.741) |
| Pomatomidae | 1 | 0.296 (NA-NA) |
| Octopodidae | 2 | 0.276 (0.213-0.338) |
| Sparidae | 17 | 0.249 (-0.039-0.537) |
| Portunidae | 1 | 0.247 (NA-NA) |
| Trichodontidae | 1 | 0.244 (NA-NA) |
| Mugilidae | 1 | 0.231 (NA-NA) |
| Centrolophidae | 4 | 0.223 (0.012-0.433) |
| Clupeidae | 46 | 0.212 (-0.048-0.471) |
| Scyliorhinidae | 3 | 0.211 (0.019-0.404) |
| Carangidae | 16 | 0.202 (-0.002-0.407) |
| Lotidae | 9 | 0.194 (0.055-0.334) |
| Rachycentridae | 2 | 0.192 (-0.125-0.509) |
| Gadidae | 68 | 0.188 (0.005-0.372) |
| Scombridae | 36 | 0.187 (-0.121-0.496) |
| Pectinidae | 4 | 0.187 (-0.023-0.396) |
| Penaeidae | 9 | 0.183 (0.03-0.335) |
| Scophthalmidae | 11 | 0.181 (0.013-0.348) |
| Soleidae | 9 | 0.18 (0.028-0.332) |
| Oregoniidae | 12 | 0.179 (-0.023-0.382) |
| Aristeidae | 3 | 0.172 (0.103-0.24) |
| Pandalidae | 28 | 0.17 (-0.074-0.414) |
| Merlucciidae | 25 | 0.169 (-0.014-0.353) |
| Munididae | 4 | 0.165 (0.055-0.275) |
| Haemulidae | 2 | 0.163 (-0.039-0.365) |
| Xiphiidae | 6 | 0.154 (-0.044-0.353) |
| Pleuronectidae | 66 | 0.151 (-0.128-0.431) |
| Haliotidae | 6 | 0.15 (0.033-0.266) |
| Nephropidae | 40 | 0.149 (-0.015-0.312) |
| Paralichthyidae | 5 | 0.147 (0.025-0.269) |
| Serranidae | 9 | 0.146 (-0.082-0.374) |
| Argentinidae | 4 | 0.145 (-0.008-0.297) |
| Moronidae | 3 | 0.143 (0.119-0.168) |
| Phycidae | 7 | 0.142 (-0.02-0.304) |
| Lophiidae | 9 | 0.14 (-0.017-0.297) |
| Stromateidae | 1 | 0.135 (NA-NA) |
| Mactridae | 3 | 0.131 (-0.006-0.269) |
| Macrouridae | 1 | 0.131 (NA-NA) |
| Triglidae | 1 | 0.13 (NA-NA) |
| Loliginidae | 1 | 0.125 (NA-NA) |
| Istiophoridae | 10 | 0.124 (0.006-0.243) |
| Moridae | 1 | 0.121 (NA-NA) |
| Ophidiidae | 12 | 0.118 (-0.043-0.278) |
| Palinuridae | 18 | 0.116 (-0.145-0.376) |
| Platycephalidae | 2 | 0.109 (0.013-0.204) |
| Gempylidae | 2 | 0.107 (-0.023-0.238) |
| Trichiuridae | 1 | 0.101 (NA-NA) |
| Uranoscopidae | 1 | 0.1 (NA-NA) |
| Malacanthidae | 4 | 0.096 (0.045-0.147) |
| Epigonidae | 2 | 0.091 (-0.036-0.219) |
| Arcticidae | 1 | 0.088 (NA-NA) |
| Hexagrammidae | 5 | 0.086 (-0.074-0.246) |
| Osmeridae | 4 | 0.083 (0.025-0.14) |
| Berycidae | 2 | 0.081 (-0.114-0.277) |
| Sillaginidae | 1 | 0.08 (NA-NA) |
| Trachichthyidae | 3 | 0.08 (0.001-0.159) |
| Cottidae | 3 | 0.075 (0.059-0.09) |
| Lithodidae | 5 | 0.074 (0.003-0.146) |
| Arripidae | 1 | 0.067 (NA-NA) |
| Cheilodactylidae | 3 | 0.066 (0.056-0.076) |
| Nototheniidae | 3 | 0.056 (-0.066-0.178) |
| Menippidae | 1 | 0.056 (NA-NA) |
| Arhynchobatidae | 1 | 0.056 (NA-NA) |
| Cyclopteridae | 1 | 0.055 (NA-NA) |
| Triakidae | 1 | 0.055 (NA-NA) |
| Rajidae | 20 | 0.053 (-0.011-0.117) |
| Anoplopomatidae | 3 | 0.053 (-0.011-0.116) |
| Anarhichadidae | 1 | 0.052 (NA-NA) |
| Lutjanidae | 8 | 0.052 (-0.073-0.177) |
| Squalidae | 5 | 0.042 (-0.028-0.112) |
| Scorpaenidae | 3 | 0.042 (0.035-0.048) |
| Sebastidae | 53 | 0.032 (-0.05-0.115) |
| Carcharhinidae | 4 | 0.03 (-0.01-0.069) |
| Lamnidae | 3 | 0.025 (0.007-0.043) |
| Hiatellidae | 1 | 0.024 (NA-NA) |
| Oreosomatidae | 5 | 0.024 (-0.004-0.052) |
| Zoarcidae | 1 | 0.005 (NA-NA) |
| Sphyrnidae | 1 | -0.002 (NA-NA) |
| Balistidae | 1 | -0.018 (NA-NA) |

**TABLE S2** | Stock-specific details about basic information, surplus production model, productivity hindcast, climate change effects and productivity forecast. Forecast results were based on data from IPSL-CM6A-LR.

*See attached file.*

**TABLE S3** | Dynamic Factor Analysis (DFA) model comparisons. logLik: log-likelihood, AICc: sample-size-corrected Akaike Information Criterion. Bold figures show the best DFA model for each FAO major fishing area (Area).

| Area | Number of trends | Variance–covariance matrix structure | logLik | AICc |
| --- | --- | --- | --- | --- |
| 21 | 1 | Same variances & no covariance | -6388.15 | 13058.22 |
|  |  | Different variances & no covariance | -6194.86 | 12965.74 |
|  |  | Same variances & same covariance | -6375.93 | 13035.89 |
|  | **2** | Same variances & no covariance | -5888.99 | 12354.00 |
|  |  | **Different variances & no covariance** | **-5556.94** | **12001.62** |
|  |  | Same variances & same covariance | -5878.24 | 12334.74 |
| 27 | 1 | Same variances & no covariance | -6206.92 | 12717.13 |
|  |  | Different variances & no covariance | -6016.93 | 12655.58 |
|  |  | Same variances & same covariance | -6158.78 | 12622.99 |
|  | **2** | Same variances & no covariance | -5633.82 | 11889.36 |
|  |  | **Different variances & no covariance** | **-5205.76** | **11372.68** |
|  |  | Same variances & same covariance | -5625.44 | 11874.86 |
| 31 | 1 | Same variances & no covariance | -2110.60 | 4309.38 |
|  |  | Different variances & no covariance | -1849.56 | 3875.52 |
|  |  | Same variances & same covariance | -2110.54 | 4311.36 |
|  | **2** | Same variances & no covariance | -1834.22 | 3844.83 |
|  |  | **Different variances & no covariance** | **-1486.63** | **3242.23** |
|  |  | Same variances & same covariance | -1832.36 | 3843.33 |
| 34 | 1 | Same variances & no covariance | -759.45 | 1581.76 |
|  |  | Different variances & no covariance | -697.87 | 1522.74 |
|  |  | Same variances & same covariance | -745.36 | 1555.78 |
|  | **2** | Same variances & no covariance | -604.24 | 1335.49 |
|  |  | **Different variances & no covariance** | **-452.15** | **1101.49** |
|  |  | Same variances & same covariance | -602.61 | 1334.61 |
| 37 | 1 | Same variances & no covariance | -516.45 | 1106.90 |
|  |  | Different variances & no covariance | -479.84 | 1115.92 |
|  |  | Same variances & same covariance | -515.17 | 1106.71 |
|  | **2** | **Same variances & no covariance** | **-444.19** | **1044.62** |
|  |  | Different variances & no covariance | -399.07 | 1052.41 |
|  |  | Same variances & same covariance | -443.87 | 1046.76 |
| 41 | 1 | Same variances & no covariance | -422.74 | 872.54 |
|  |  | Different variances & no covariance | -353.45 | 758.54 |
|  |  | Same variances & same covariance | -422.25 | 873.74 |
|  | **2** | Same variances & no covariance | -393.82 | 839.28 |
|  |  | **Different variances & no covariance** | **-305.73** | **689.35** |
|  |  | Same variances & same covariance | -400.19 | 854.34 |
| 47 | 1 | Same variances & no covariance | -1427.91 | 2896.57 |
|  |  | Different variances & no covariance | -1347.66 | 2774.02 |
|  |  | Same variances & same covariance | -1427.78 | 2898.40 |
|  | **2** | Same variances & no covariance | -1208.68 | 2496.07 |
|  |  | **Different variances & no covariance** | **-1062.91** | **2243.73** |
|  |  | Same variances & same covariance | -1206.06 | 2492.97 |
| 57 | 1 | Same variances & no covariance | -495.54 | 1028.57 |
|  |  | Different variances & no covariance | -432.24 | 937.83 |
|  |  | Same variances & same covariance | -492.65 | 1024.95 |
|  | **2** | Same variances & no covariance | -444.80 | 962.94 |
|  |  | **Different variances & no covariance** | **-334.57** | **781.02** |
|  |  | Same variances & same covariance | -415.65 | 906.98 |
| 61 | 1 | Same variances & no covariance | -1742.42 | 3573.62 |
|  |  | Different variances & no covariance | -1633.85 | 3446.55 |
|  |  | Same variances & same covariance | -1742.31 | 3575.53 |
|  | **2** | Same variances & no covariance | -1562.03 | 3302.90 |
|  |  | **Different variances & no covariance** | **-1365.00** | **3004.68** |
|  |  | Same variances & same covariance | -1561.93 | 3304.98 |
| 67 | 1 | Same variances & no covariance | -4895.33 | 10019.02 |
|  |  | Different variances & no covariance | -4498.72 | 9463.05 |
|  |  | Same variances & same covariance | -4895.32 | 10021.12 |
|  | **2** | Same variances & no covariance | -4222.02 | 8909.65 |
|  |  | **Different variances & no covariance** | **-3616.02** | **7948.92** |
|  |  | Same variances & same covariance | -4221.27 | 8910.39 |
| 71 | 1 | Same variances & no covariance | -417.35 | 853.21 |
|  |  | Different variances & no covariance | -344.77 | 723.12 |
|  |  | Same variances & same covariance | -409.33 | 839.29 |
|  | **2** | Same variances & no covariance | -364.10 | 761.79 |
|  |  | **Different variances & no covariance** | **-265.43** | **580.16** |
|  |  | Same variances & same covariance | -373.37 | 782.52 |
| 77 | 1 | Same variances & no covariance | -1838.99 | 3737.13 |
|  |  | Different variances & no covariance | -1645.69 | 3407.66 |
|  |  | Same variances & same covariance | -1834.09 | 3729.40 |
|  | **2** | Same variances & no covariance | -1450.25 | 3016.78 |
|  |  | **Different variances & no covariance** | **-1292.60** | **2760.70** |
|  |  | Same variances & same covariance | -1426.79 | 2972.01 |
| 81 | 1 | Same variances & no covariance | -2126.55 | 4379.09 |
|  |  | Different variances & no covariance | -1817.83 | 3891.48 |
|  |  | Same variances & same covariance | -2077.92 | 4283.97 |
|  | **2** | Same variances & no covariance | -1683.15 | 3622.13 |
|  |  | **Different variances & no covariance** | **-1182.99** | **2760.26** |
|  |  | Same variances & same covariance | -1644.08 | 3546.26 |
| 87 | 1 | Same variances & no covariance | -945.32 | 1942.41 |
|  |  | Different variances & no covariance | -904.97 | 1912.54 |
|  |  | Same variances & same covariance | -944.07 | 1942.05 |
|  | **2** | Same variances & no covariance | -867.69 | 1837.98 |
|  |  | **Different variances & no covariance** | **-762.63** | **1682.09** |
|  |  | Same variances & same covariance | -864.33 | 1833.54 |

**TABLE S4** | Projected global productivity change in the 2090s compared to the 2020s. SSP: Shared Socioeconomic Pathways. ESM: Earth System Model. Productivity change and percentage were calculated with mean productivity in the 2090s as the focus and in the 2020s as the reference.

| SSP | ESM | Productivity change | Productivity change percentage |
| --- | --- | --- | --- |
| SSP1-2.6 | IPSL-CM6A-LR | -0.0015  (-0.0025 - -0.0005) | -0.99%  (-1.64% - -0.33%) |
|  | MPI-ESM1-2-LR | -0.0004  (-0.0010 - 0.0002) | -0.23%  (-0.59% - 0.11%) |
|  | NorESM2-LM | -0.0007  (-0.0014 - 0.0001) | -0.44%  (-0.91% - 0.04%) |
| SSP2-4.5 | IPSL-CM6A-LR | -0.0017  (-0.0040 - 0.0007) | -1.12%  (-2.61% - 0.47%) |
|  | MPI-ESM1-2-LR | -0.0009  (-0.0025 - 0.0008) | -0.56%  (-1.50% - 0.53%) |
|  | NorESM2-LM | -0.0011  (-0.0029 - 0.0010) | -0.68%  (-1.88% - 0.62%) |
| SSP5-8.5 | IPSL-CM6A-LR | -0.0045  (-0.0095 - 0.0006) | -2.95%  (-6.31% - 0.38%) |
|  | MPI-ESM1-2-LR | -0.0014  (-0.0047 - 0.0020) | -0.85%  (-2.81% - 1.26%) |
|  | NorESM2-LM | -0.0012  (-0.0053 - 0.0031) | -0.79%  (-3.40% - 2.03%) |

**TABLE S5** | Projected FAO major fishing area (Area) productivity change in the 2090s compared to the 2020s. SSP: Shared Socioeconomic Pathways. ESM: Earth System Model. Productivity change and percentage were calculated with mean productivity in the 2090s as the focus and in the 2020s as the reference.

| Area | SSP | ESM | Productivity change | Productivity change percentage |
| --- | --- | --- | --- | --- |
| 21 | SSP1-2.6 | IPSL-CM6A-LR | -0.0003  (-0.0014 - 0.0008) | -0.27%  (-1.21% - 0.63%) |
|  |  | MPI-ESM1-2-LR | -0.0019  (-0.0034 - -0.0007) | -1.94%  (-3.83% - -0.59%) |
|  |  | NorESM2-LM | -0.0004  (-0.0014 - 0.0006) | -0.42%  (-1.53% - 0.50%) |
|  | SSP2-4.5 | IPSL-CM6A-LR | -0.0004  (-0.0034 - 0.0024) | -0.38%  (-2.89% - 2.05%) |
|  |  | MPI-ESM1-2-LR | -0.0003  (-0.0022 - 0.0015) | -0.34%  (-2.22% - 1.48%) |
|  |  | NorESM2-LM | -0.0009  (-0.0035 - 0.0015) | -0.92%  (-3.69% - 1.25%) |
|  | SSP5-8.5 | IPSL-CM6A-LR | -0.0019  (-0.0085 - 0.0042) | -1.64%  (-7.29% - 3.54%) |
|  |  | MPI-ESM1-2-LR | 0.0020  (-0.0028 - 0.0069) | 2.02%  (-2.71% - 6.90%) |
|  |  | NorESM2-LM | -0.0020  (-0.0074 - 0.0031) | -2.04%  (-8.16% - 2.61%) |
| 27 | SSP1-2.6 | IPSL-CM6A-LR | -0.0057 (-0.0089 - -0.0022) | -3.31%  (-5.21% - -1.25%) |
|  |  | MPI-ESM1-2-LR | -0.0011  (-0.0020 - 0.0000) | -0.51%  (-0.93% - -0.02%) |
|  |  | NorESM2-LM | -0.0016  (-0.0033 - 0.0002) | -0.79%  (-1.54% - 0.11%) |
|  | SSP2-4.5 | IPSL-CM6A-LR | -0.0065  (-0.0106 - -0.0023) | -3.83%  (-6.29% - -1.31%) |
|  |  | MPI-ESM1-2-LR | -0.0043  (-0.0090 - 0.0005) | -1.99%  (-3.89% - 0.29%) |
|  |  | NorESM2-LM | -0.0040  (-0.0089 - 0.0008) | -1.98%  (-4.06% - 0.50%) |
|  | SSP5-8.5 | IPSL-CM6A-LR | -0.0171  (-0.0279 - -0.0057) | -9.88%  (-16.07% - -3.19%) |
|  |  | MPI-ESM1-2-LR | -0.0073  (-0.0151 - 0.0007) | -3.45%  (-6.62% - 0.39%) |
|  |  | NorESM2-LM | -0.0085  (-0.0183 - 0.0012) | -4.19%  (-8.37% - 0.68%) |
| 31 | SSP1-2.6 | IPSL-CM6A-LR | 0.0007  (-0.0013 - 0.0027) | 0.54%  (-0.89% - 1.93%) |
|  |  | MPI-ESM1-2-LR | -0.0016  (-0.0031 - -0.0005) | -1.13%  (-2.30% - -0.32%) |
|  |  | NorESM2-LM | -0.0006  (-0.0016 - 0.0004) | -0.46%  (-1.16% - 0.26%) |
|  | SSP2-4.5 | IPSL-CM6A-LR | 0.0004  (-0.0040 - 0.0047) | 0.32%  (-2.77% - 3.37%) |
|  |  | MPI-ESM1-2-LR | -0.0017  (-0.0033 - 0.0000) | -1.16%  (-2.27% - -0.02%) |
|  |  | NorESM2-LM | -0.0013  (-0.0028 - 0.0005) | -0.97%  (-2.19% - 0.30%) |
|  | SSP5-8.5 | IPSL-CM6A-LR | -0.0028  (-0.0084 - 0.0029) | -1.97%  (-5.75% - 2.15%) |
|  |  | MPI-ESM1-2-LR | -0.0026  (-0.0058 - 0.0010) | -1.76%  (-3.85% - 0.73%) |
|  |  | NorESM2-LM | -0.0033  (-0.0062 - -0.0001) | -2.35%  (-4.59% - -0.06%) |
| 34 | SSP1-2.6 | IPSL-CM6A-LR | 0.0009  (-0.0022 - 0.0042) | 0.43%  (-1.01% - 2.18%) |
|  |  | MPI-ESM1-2-LR | 0.0021  (-0.0047 - 0.0090) | 1.10%  (-2.08% - 4.69%) |
|  |  | NorESM2-LM | -0.0012  (-0.0055 - 0.0031) | -0.58%  (-2.68% - 1.48%) |
|  | SSP2-4.5 | IPSL-CM6A-LR | -0.0032  (-0.0090 - 0.0032) | -1.61%  (-4.62% - 1.40%) |
|  |  | MPI-ESM1-2-LR | 0.0013  (-0.0125 - 0.0153) | 0.69%  (-5.72% - 7.67%) |
|  |  | NorESM2-LM | -0.0030  (-0.0100 - 0.0048) | -1.55%  (-5.27% - 2.12%) |
|  | SSP5-8.5 | IPSL-CM6A-LR | -0.0059  (-0.0181 - 0.0078) | -2.99%  (-9.40% - 3.42%) |
|  |  | MPI-ESM1-2-LR | -0.0010  (-0.0161 - 0.0160) | -0.60%  (-8.31% - 7.43%) |
|  |  | NorESM2-LM | -0.0061  (-0.0183 - 0.0071) | -3.21%  (-9.86% - 3.04%) |
| 37 | SSP1-2.6 | IPSL-CM6A-LR | 0.0010  (-0.0051 - 0.0078) | 0.42%  (-2.06% - 3.28%) |
|  |  | MPI-ESM1-2-LR | -0.0001  (-0.0013 - 0.0010) | -0.05%  (-0.51% - 0.44%) |
|  |  | NorESM2-LM | 0.0024  (-0.0035 - 0.0093) | 0.93%  (-1.71% - 3.52%) |
|  | SSP2-4.5 | IPSL-CM6A-LR | 0.0042  (-0.0122 - 0.0237) | 1.75%  (-5.01% - 9.95%) |
|  |  | MPI-ESM1-2-LR | 0.0026  (-0.0040 - 0.0104) | 1.02%  (-1.80% - 4.11%) |
|  |  | NorESM2-LM | 0.0049  (-0.0110 - 0.0232) | 1.87%  (-5.48% - 8.84%) |
|  | SSP5-8.5 | IPSL-CM6A-LR | 0.0072  (-0.0277 - 0.0471) | 2.93%  (-11.79% - 19.33%) |
|  |  | MPI-ESM1-2-LR | 0.0021  (-0.0166 - 0.0227) | 0.76%  (-7.22% - 9.10%) |
|  |  | NorESM2-LM | 0.0084  (-0.0235 - 0.0445) | 3.06%  (-11.46% - 16.80%) |
| 41 | SSP1-2.6 | IPSL-CM6A-LR | -0.0014  (-0.0039 - 0.0012) | -0.62%  (-1.61% - 0.55%) |
|  |  | MPI-ESM1-2-LR | -0.0020  (-0.0094 - 0.0053) | -0.96%  (-4.45% - 2.80%) |
|  |  | NorESM2-LM | -0.0050  (-0.0126 - 0.0029) | -3.00%  (-9.31% - 1.20%) |
|  | SSP2-4.5 | IPSL-CM6A-LR | 0.0028  (-0.0020 - 0.0083) | 1.25%  (-0.79% - 3.85%) |
|  |  | MPI-ESM1-2-LR | -0.0065  (-0.0143 - 0.0008) | -3.09%  (-6.54% - 0.41%) |
|  |  | NorESM2-LM | -0.0025  (-0.0112 - 0.0069) | -1.71%  (-8.03% - 2.90%) |
|  | SSP5-8.5 | IPSL-CM6A-LR | 0.0076  (-0.0009 - 0.0188) | 3.38%  (-0.40% - 8.16%) |
|  |  | MPI-ESM1-2-LR | 0.0031  (-0.0080 - 0.0138) | 1.73%  (-3.70% - 7.77%) |
|  |  | NorESM2-LM | 0.0048  (-0.0004 - 0.0102) | 2.78%  (-0.18% - 7.52%) |
| 47 | SSP1-2.6 | IPSL-CM6A-LR | -0.0024  (-0.0037 - -0.0012) | -2.04%  (-3.06% - -1.05%) |
|  |  | MPI-ESM1-2-LR | 0.0003  (-0.0016 - 0.0022) | 0.32%  (-1.36% - 2.31%) |
|  |  | NorESM2-LM | -0.0011  (-0.0022 - 0.0000) | -1.03%  (-2.12% - 0.01%) |
|  | SSP2-4.5 | IPSL-CM6A-LR | -0.0052  (-0.0083 - -0.0021) | -4.41%  (-7.17% - -1.76%) |
|  |  | MPI-ESM1-2-LR | -0.0023  (-0.0055 - 0.0011) | -2.05%  (-4.73% - 1.20%) |
|  |  | NorESM2-LM | -0.0033  (-0.0056 - -0.0010) | -3.12%  (-6.00% - -0.84%) |
|  | SSP5-8.5 | IPSL-CM6A-LR | -0.0124  (-0.0193 - -0.0060) | -10.52%  (-16.68% - -4.60%) |
|  |  | MPI-ESM1-2-LR | -0.0095  (-0.0158 - -0.0034) | -8.73%  (-14.27% - -3.15%) |
|  |  | NorESM2-LM | -0.0076  (-0.0123 - -0.0031) | -7.11%  (-13.17% - -2.37%) |
| 57 | SSP1-2.6 | IPSL-CM6A-LR | 0.0028  (0.0004 - 0.0053) | 1.43%  (0.24% - 2.68%) |
|  |  | MPI-ESM1-2-LR | -0.0015  (-0.0031 - 0.0000) | -0.79%  (-1.58% - -0.03%) |
|  |  | NorESM2-LM | 0.0023  (0.0010 - 0.0038) | 1.28%  (0.57% - 2.00%) |
|  | SSP2-4.5 | IPSL-CM6A-LR | 0.0040  (-0.0008 - 0.0093) | 2.05%  (-0.42% - 4.60%) |
|  |  | MPI-ESM1-2-LR | 0.0050  (0.0023 - 0.0079) | 2.78%  (1.24% - 4.46%) |
|  |  | NorESM2-LM | 0.0067  (0.0028 - 0.0110) | 3.66%  (1.61% - 5.80%) |
|  | SSP5-8.5 | IPSL-CM6A-LR | 0.0105  (-0.0008 - 0.0224) | 5.40%  (-0.41% - 11.24%) |
|  |  | MPI-ESM1-2-LR | 0.0064  (0.0012 - 0.0115) | 3.52%  (0.69% - 6.57%) |
|  |  | NorESM2-LM | 0.0129  (0.0055 - 0.0211) | 7.03%  (3.14% - 11.01%) |
| 61 | SSP1-2.6 | IPSL-CM6A-LR | -0.0078  (-0.0113 - -0.0044) | -3.19%  (-4.63% - -1.82%) |
|  |  | MPI-ESM1-2-LR | -0.0003  (-0.0017 - 0.0011) | -0.13%  (-0.70% - 0.46%) |
|  |  | NorESM2-LM | 0.0006  (-0.0009 - 0.0021) | 0.26%  (-0.34% - 0.88%) |
|  | SSP2-4.5 | IPSL-CM6A-LR | -0.0149  (-0.0215 - -0.0086) | -6.10%  (-8.64% - -3.46%) |
|  |  | MPI-ESM1-2-LR | -0.0012  (-0.0041 - 0.0017) | -0.46%  (-1.63% - 0.71%) |
|  |  | NorESM2-LM | -0.0003  (-0.0041 - 0.0032) | -0.12%  (-1.56% - 1.41%) |
|  | SSP5-8.5 | IPSL-CM6A-LR | -0.0245  (-0.0359 - -0.0129) | -9.93%  (-14.00% - -5.54%) |
|  |  | MPI-ESM1-2-LR | -0.0030  (-0.0076 - 0.0015) | -1.21%  (-2.90% - 0.57%) |
|  |  | NorESM2-LM | -0.0020  (-0.0112 - 0.0068) | -0.79%  (-4.23% - 2.87%) |
| 67 | SSP1-2.6 | IPSL-CM6A-LR | 0.0013  (-0.0002 - 0.0027) | 1.67%  (-0.30% - 3.58%) |
|  |  | MPI-ESM1-2-LR | 0.0007  (0.0002 - 0.0012) | 0.78%  (0.25% - 1.27%) |
|  |  | NorESM2-LM | -0.0002  (-0.0007 - 0.0003) | -0.22%  (-0.89% - 0.37%) |
|  | SSP2-4.5 | IPSL-CM6A-LR | 0.0024  (-0.0015 - 0.0059) | 3.05%  (-1.84% - 7.95%) |
|  |  | MPI-ESM1-2-LR | 0.0006  (-0.0013 - 0.0025) | 0.71%  (-1.25% - 2.75%) |
|  |  | NorESM2-LM | 0.0006  (-0.0011 - 0.0022) | 0.65%  (-1.36% - 2.52%) |
|  | SSP5-8.5 | IPSL-CM6A-LR | 0.0041  (-0.0021 - 0.0101) | 5.25%  (-2.56% - 13.43%) |
|  |  | MPI-ESM1-2-LR | 0.0008  (-0.0031 - 0.0045) | 0.87%  (-3.01% - 4.91%) |
|  |  | NorESM2-LM | 0.0029  (-0.0011 - 0.0069) | 3.40%  (-1.34% - 8.10%) |
| 71 | SSP1-2.6 | IPSL-CM6A-LR | 0.0002  (-0.0022 - 0.0027) | 0.11%  (-1.08% - 1.30%) |
|  |  | MPI-ESM1-2-LR | -0.0003  (-0.0013 - 0.0006) | -0.18%  (-0.71% - 0.49%) |
|  |  | NorESM2-LM | 0.0006  (-0.0013 - 0.0025) | 0.38%  (-0.69% - 1.66%) |
|  | SSP2-4.5 | IPSL-CM6A-LR | 0.0020  (-0.0061 - 0.0103) | 0.95%  (-3.00% - 5.01%) |
|  |  | MPI-ESM1-2-LR | 0.0022  (-0.0019 - 0.0066) | 1.44%  (-0.96% - 5.00%) |
|  |  | NorESM2-LM | 0.0011  (-0.0034 - 0.0059) | 0.67%  (-2.09% - 3.49%) |
|  | SSP5-8.5 | IPSL-CM6A-LR | 0.0031  (-0.0149 - 0.0217) | 1.51%  (-7.40% - 10.65%) |
|  |  | MPI-ESM1-2-LR | 0.0075  (-0.0037 - 0.0201) | 4.90%  (-1.85% - 15.82%) |
|  |  | NorESM2-LM | 0.0014  (-0.0089 - 0.0118) | 0.81%  (-5.22% - 6.92%) |
| 77 | SSP1-2.6 | IPSL-CM6A-LR | 0.0012  (-0.0006 - 0.0029) | 1.19%  (-0.62% - 2.93%) |
|  |  | MPI-ESM1-2-LR | 0.0012  (0.0005 - 0.0019) | 1.01%  (0.47% - 1.55%) |
|  |  | NorESM2-LM | -0.0015  (-0.0027 - -0.0004) | -1.48%  (-2.72% - -0.33%) |
|  | SSP2-4.5 | IPSL-CM6A-LR | 0.0026  (-0.0018 - 0.0069) | 2.56%  (-1.76% - 6.96%) |
|  |  | MPI-ESM1-2-LR | 0.0003  (-0.0017 - 0.0021) | 0.23%  (-1.36% - 1.90%) |
|  |  | NorESM2-LM | -0.0016  (-0.0037 - 0.0005) | -1.56%  (-3.73% - 0.45%) |
|  | SSP5-8.5 | IPSL-CM6A-LR | 0.0027  (-0.0049 - 0.0101) | 2.71%  (-4.78% - 10.18%) |
|  |  | MPI-ESM1-2-LR | -0.0012  (-0.0055 - 0.0032) | -0.94%  (-4.52% - 2.73%) |
|  |  | NorESM2-LM | -0.0004  (-0.0048 - 0.0037) | -0.47%  (-4.79% - 3.27%) |
| 81 | SSP1-2.6 | IPSL-CM6A-LR | 0.0017  (0.0001 - 0.0035) | 0.98%  (0.09% - 1.98%) |
|  |  | MPI-ESM1-2-LR | -0.0002  (-0.0010 - 0.0006) | -0.12%  (-0.51% - 0.33%) |
|  |  | NorESM2-LM | 0.0009  (-0.0006 - 0.0025) | 0.62%  (-0.37% - 1.58%) |
|  | SSP2-4.5 | IPSL-CM6A-LR | 0.0046  (0.0007 - 0.0091) | 2.73%  (0.44% - 5.29%) |
|  |  | MPI-ESM1-2-LR | 0.0007  (-0.0013 - 0.0031) | 0.38%  (-0.67% - 1.66%) |
|  |  | NorESM2-LM | 0.0028  (0.0003 - 0.0057) | 1.80%  (0.17% - 3.56%) |
|  | SSP5-8.5 | IPSL-CM6A-LR | 0.0091  (0.0012 - 0.0185) | 5.38%  (0.72% - 10.75%) |
|  |  | MPI-ESM1-2-LR | 0.0028  (-0.0015 - 0.0078) | 1.52%  (-0.84% - 4.26%) |
|  |  | NorESM2-LM | 0.0083  (0.0021 - 0.0150) | 5.43%  (1.38% - 9.74%) |
| 87 | SSP1-2.6 | IPSL-CM6A-LR | -0.0073  (-0.0118 - -0.0031) | -3.51%  (-5.46% - -1.63%) |
|  |  | MPI-ESM1-2-LR | 0.0040  (0.0007 - 0.0075) | 2.22%  (0.39% - 4.19%) |
|  |  | NorESM2-LM | -0.0066  (-0.0102 - -0.0029) | -6.34%  (-14.28% - -2.06%) |
|  | SSP2-4.5 | IPSL-CM6A-LR | -0.0074  (-0.0134 - -0.0024) | -3.60%  (-6.29% - -1.25%) |
|  |  | MPI-ESM1-2-LR | -0.0001  (-0.0036 - 0.0033) | -0.06%  (-1.94% - 1.78%) |
|  |  | NorESM2-LM | -0.0079  (-0.0119 - -0.0039) | -7.44%  (-16.06% - -2.68%) |
|  | SSP5-8.5 | IPSL-CM6A-LR | -0.0126  (-0.0241 - -0.0031) | -6.05%  (-11.03% - -1.61%) |
|  |  | MPI-ESM1-2-LR | -0.0019  (-0.0085 - 0.0044) | -1.00%  (-4.51% - 2.40%) |
|  |  | NorESM2-LM | -0.0038  (-0.0098 - 0.0013) | -3.56%  (-10.17% - 1.20%) |
